# Association of postoperative modified Yaotong Tang with early recovery after unilateral biportal endoscopy for lumbar disc herniation: a retrospective comparative cohort study using propensity score weighting

**DOI:** 10.3389/fphar.2026.1852732

**Published:** 2026-07-09

**Authors:** Mingzhong Xie, Xiaorong Huang, Hao Wang, Liangjun Luo, Huacai Jiang, Huan Liu, Guoyou Wang, Chunbao Wu, Guangyou Chen

**Affiliations:** 1 Department of Spinal Surgery, The Affiliated Hospital of Traditional Chinese Medicine of Southwest Medical University, Luzhou, Sichuan, China; 2 The First Affiliated Hospital of Traditional Chinese Medicine of Chengdu Medical College XinDu Hospital of Traditional Chinese Medicine, Chengdu, Sichuan, China; 3 Department of Orthopedics, The Affiliated Hospital of Traditional Chinese Medicine of Southwest Medical University, Luzhou, Sichuan, China; 4 Department of Spinal Surgery, Orthopedic Hospital, Chongqing University of Chinese Medicine, Chongqing, China

**Keywords:** Aconiti Radix Cocta, functional recovery, inflammatory biomarkers, inverse probability of treatment weighting, lumbar disc herniation, modified Yaotong Tang, postoperative pain, unilateral biportal endoscopy

## Abstract

**Introduction:**

Modified Yaotong Tang (MYT) is used as an adjunctive postoperative herbal decoction in traditional Chinese medicine practice for lumbar disorders, but its association with early recovery after unilateral biportal endoscopy (UBE) and the reproducibility of its clinical preparation remain insufficiently characterized.

**Methods:**

This single-center retrospective comparative cohort study screened 235 patients with symptomatic single-level lumbar disc herniation (LDH) who underwent surgical treatment between September 2022 and September 2025. Of these, 182 met the eligibility criteria, including 122 patients in the primary UBE analytic cohort (62 UBE + MYT and 60 UBE alone) and 60 patients in a transforaminal lumbar interbody fusion cohort retained only as a descriptive external benchmark. The primary UBE comparison was adjusted using inverse probability of treatment weighting (IPTW).

**Results:**

Compared with UBE alone, the UBE + MYT cohort showed lower IPTW-adjusted Visual Analog Scale scores at postoperative day (POD) 3 (3.88 vs. 4.67; mean difference, −0.79; 95% CI, −1.06 to −0.53; P < 0.001) and POD7 (2.85 vs. 3.73; mean difference, −0.88; 95% CI, −1.14 to −0.62; P < 0.001). IPTW-adjusted IL-6 and CRP levels were lower during the first postoperative week, and follow-up Japanese Orthopaedic Association scores were higher in the UBE + MYT cohort. MYT administration was verified using de-identified medical, pharmacy/decoction-room, nursing, and follow-up records. No Clavien-Dindo grade III or higher adverse events were observed.

**Discussion:**

Postoperative MYT use was associated with lower early postoperative pain scores, lower inflammatory biomarker levels, and better subsequent functional recovery after UBE for LDH. No severe safety signal was observed, but causality and rare herb-related safety events require prospective validation.

## 1 Introduction

Lumbar disc herniation (LDH) is a major cause of low back pain and radiculopathy and remains an important source of functional impairment and healthcare burden worldwide (Hartvigsen et al., 2018). Surgical treatment is considered when symptoms persist despite adequate conservative management. In recent years, minimally invasive decompression techniques have been increasingly adopted to reduce surgical trauma and facilitate postoperative recovery (Heo et al., 2017; Zhang et al., 2025; Li et al., 2025). Among these approaches, unilateral biportal endoscopy (UBE) has attracted growing clinical interest because it provides independent visualization and working channels, flexible instrument handling, and satisfactory decompression under magnified endoscopic visualization (Heo et al., 2017; Zhang et al., 2025; Li et al., 2025).

However, ‘minimally invasive’ does not mean ‘non-invasive.’ Surgical manipulation, canal reaming, irrigation pressure, and nerve root retraction during UBE may trigger local and systemic postoperative inflammatory responses. Inflammatory biomarkers such as interleukin-6 (IL-6) and C-reactive protein (CRP) are commonly used to characterize early postoperative inflammatory burden after spine surgery (Roch et al., 2024). Early postoperative pain and inflammation are clinically relevant because insufficient early recovery may impair mobilization, prolong hospitalization, increase rescue analgesic requirements, and delay functional rehabilitation (Glare et al., 2019; Chou et al., 2016).

Enhanced Recovery After Surgery (ERAS) protocols advocate multimodal strategies to mitigate surgical stress and improve postoperative recovery (Debono et al., 2021; Bratzler et al., 2013; Berrios-Torres et al., 2017; Kehlet and Wilmore, 2008). Although non-steroidal anti-inflammatory drugs and rescue analgesics remain important components of routine perioperative pain management, adjunctive strategies that may support early recovery while maintaining safety transparency merit further evaluation (Chou et al., 2016; Bratzler et al., 2013; Berrios-Torres et al., 2017).

Within traditional Chinese medicine practice, postoperative lumbar pain and delayed recovery are often interpreted as patterns involving qi stagnation, blood stasis, cold-dampness obstruction, and deficiency-related impaired recovery. MYT is a multi-herb decoction used in this clinical context as an adjunctive postoperative herbal formula. Nevertheless, modern clinical evidence regarding MYT after endoscopic spine surgery remains limited, and rigorous reporting of multi-herb preparation, processing, batch traceability, and safety monitoring is essential for reproducibility and interpretation (Heinrich et al., 2022).

The present study was therefore designed as a single-center retrospective comparative cohort study to evaluate whether documented postoperative MYT use was associated with early postoperative recovery among patients undergoing UBE for LDH. The primary comparison was restricted to the UBE + MYT and UBE-alone cohorts and adjusted using IPTW. A contemporaneous TLIF cohort was retained only as a descriptive external benchmark because of substantial indication-related differences. In response to the need for transparent reporting of herbal interventions, this revised manuscript also provides additional information on MYT pharmacopoeial identity, hospital decoction procedure, treatment-course verification, available batch-level quality-control information, and safety assessment.

## 2 Materials and methods

### 2.1 Study design and ethics

This was a single-center retrospective comparative cohort study conducted at The Affiliated Hospital of Traditional Chinese Medicine of Southwest Medical University between September 2022 and September 2025. Consecutive patients with symptomatic single-level LDH who underwent surgical treatment were identified from the institutional electronic medical record system, operative registry, laboratory database, pharmacy/decoction-room records, nursing administration records, imaging archive, and follow-up database. The study is reported in accordance with the principles of the STROBE statement for observational studies (von Elm et al., 2007).

The primary adjusted comparison was prespecified between patients treated with UBE plus MYT and those treated with UBE alone under standard perioperative management. A contemporaneous cohort treated with TLIF was retained only as a descriptive external benchmark because of substantial baseline and indication-related differences and was not included in the primary IPTW-adjusted comparison.

The study protocol was reviewed and approved by the Ethics Committee of The Affiliated Hospital of Traditional Chinese Medicine of Southwest Medical University (approval No. BY2026014). This retrospective analysis was conducted in accordance with the Declaration of Helsinki and local regulatory requirements. The requirement for individual informed consent was waived by the ethics committee because the study used existing de-identified clinical, laboratory, imaging, pharmacy, nursing, and follow-up data and involved no additional intervention or specimen collection.

### 2.2 Study population

Patients were eligible if they met all of the following criteria: (1) age 16–85 years; (2) radiologically confirmed single-level LDH consistent with clinical symptoms; (3) persistent radicular pain and/or neurological symptoms refractory to standardized conservative treatment; and (4) complete key perioperative clinical, laboratory, imaging, and follow-up data available for analysis.

Exclusion criteria were: (1) multi-level lumbar disease; (2) segmental instability requiring fixation or other conditions unsuitable for the primary UBE comparison; (3) previous lumbar surgery at the index level; (4) spinal infection, tumor, fracture, or severe deformity; (5) severe hepatic, renal, hematologic, autoimmune, or uncontrolled systemic disease; (6) documented allergy or contraindication to any component of MYT, including aconite-related preparations where documented; (7) concomitant use of other herbal decoctions or Chinese patent medicines that could substantially affect postoperative pain, inflammatory biomarkers, coagulation-related biomarkers, or safety assessment; and (8) incomplete key baseline, laboratory, imaging, or follow-up outcome data.

A total of 235 potentially eligible patients were screened. After eligibility review, 53 patients were excluded and 182 patients were included in the final analysis. The final cohort comprised 62 patients in the UBE + MYT cohort, 60 in the UBE-alone cohort, and 60 in the TLIF descriptive benchmark cohort.

### 2.3 Cohort definition and exposure

The main analytic cohort comprised patients undergoing UBE. Exposure status was defined according to documented postoperative MYT administration verified from electronic medical orders, pharmacy/decoction-room records, dispensing records, nursing administration records, and follow-up information. Patients receiving UBE plus MYT were assigned to the UBE + MYT cohort, whereas patients undergoing UBE without MYT and managed with standard perioperative care were assigned to the UBE-alone cohort. The TLIF cohort consisted of patients treated with conventional fusion surgery during the same study period and served as an external descriptive benchmark only.

Postoperative MYT exposure was defined as MYT administration after UBE during the early postoperative recovery period. MYT was not administered preoperatively. The intended course was one daily dose for 7 postoperative days, divided into morning and evening administrations. Actual initiation time, number of completed administrations, omissions, discontinuation, intolerance, and adverse events were verified using de-identified medical, pharmacy/decoction-room, nursing, and follow-up records.

In routine clinical practice, postoperative MYT was prescribed as an adjunct by the treating team after postoperative assessment, evaluation of oral tolerance, and patient acceptance of Chinese herbal treatment. The decision to prescribe MYT was not based on randomization or a prespecified institutional allocation protocol. Because of the retrospective design, the available records could not fully reconstruct the relative contributions of physician preference, patient preference, TCM syndrome assessment, or time-period practice patterns. These potential treatment-selection factors were addressed analytically using propensity-score weighting for measurable baseline covariates and were acknowledged as a possible source of residual confounding.

Before statistical analysis, the dataset underwent structured quality control, including verification of patient identifiers, review of categorical coding consistency, detection of out-of-range or implausible values, and correction of identifiable data-entry errors against source records where available.

### 2.4 Surgical and standard perioperative management

All procedures were performed by the same senior spinal surgical team according to standardized institutional protocols. UBE was carried out using a unilateral biportal endoscopic decompression technique under general anesthesia. TLIF was performed using a conventional posterior decompression and interbody fusion approach according to routine surgical indications.

Details of perioperative co-interventions are summarized in Supplementary Table S4. This standardized pathway was consistent with contemporary perioperative and antimicrobial-prophylaxis principles for surgical care, while the present study-specific comparisons relied on institutional source records rather than guideline assumptions (Debono et al., 2021; Bratzler et al., 2013; Berrios-Torres et al., 2017; Kehlet and Wilmore, 2008).

### 2.5 MYT composition, pharmacopoeial identity, and ConPhyMP-Based reporting

MYT consisted of seven Chinese herbal decoction pieces: Dipsaci Radix 24 g, Poria 15 g, Zingiberis Rhizoma 15 g, Atractylodis Macrocephalae Rhizoma 20 g, Glycyrrhizae Radix et Rhizoma Praeparata cum Melle 12 g, Citri Reticulatae Pericarpium 20 g, and Aconiti Radix Cocta 9 g. The total crude herbal dose was 115 g per daily prescription. Pharmacopoeial names, botanical sources, families, medicinal parts, processing status, daily doses, manufacturer and batch information, and certificate-of-analysis or inspection-report information for the clinically used batches are summarized in Supplementary Tables S1-S1C. Patient-level traceability was specifically established for Aconiti Radix Cocta, and batch-level COA/inspection-report coverage was collated for all seven MYT components used in the UBE + MYT cohort. Source COA/inspection reports, manufacturer qualification documents, and pharmacy traceability records are provided in Supplementary Datasheets S2-S4.

The pharmacopoeial identity of the seven herbal components was checked against the 2020 edition of the Chinese Pharmacopoeia (Chinese Pharmacopoeia Commission, 2020). In addition to the ConPhyMP recommendations (Heinrich et al., 2022), the reporting strategy was informed by broader best-practice principles for phytopharmacological research, which emphasize botanical identity, processing, quality control, chemical characterization, and transparent limitations (Heinrich et al., 2020).

### 2.6 Decoction preparation, dispensing, and administration

MYT was dispensed and decocted by the hospital pharmacy/decoction room under a unified institutional procedure. Before decoction, pharmacists verified the prescription, patient information, herbal names, daily doses, processing status, batch information, expiry dates, and available quality-control documents. Aconiti Radix Cocta was verified as processed Zhi Chuan Wu and was not substituted by raw Chuan Wu. It was weighed, labeled, and placed separately before decoction.

Except for Aconiti Radix Cocta, the remaining herbal components were soaked before decoction. Aconiti Radix Cocta 9 g was pre-decocted separately for approximately 60 min and then combined with the remaining six herbs for routine two-step decoction. The combined filtrates were concentrated to approximately 400 mL per daily dose and divided into two sealed portions of approximately 200 mL each for morning and evening administration. Sealed decoctions not taken immediately were stored according to pharmacy requirements. Decoction, packaging, dispensing, and administration records were retained by the hospital. De-identified summary information was used for the present retrospective analysis. The institutional decoction and traceability procedure is summarized in Supplementary Table S2.

### 2.7 Outcome measures

The main outcomes were early postoperative pain recovery and systemic inflammatory response. Pain was assessed using the VAS before surgery and on POD1, POD3, and POD7. Systemic inflammatory response was assessed using serum IL-6 at the same time points. The main early recovery window was POD3, with POD1 and POD7 used to characterize the early postoperative trajectory.

Secondary outcomes included CRP and D-dimer during the first postoperative week, JOA scores preoperatively and at 1, 3, 6, and 12 months postoperatively, and postoperative adverse events. Exploratory outcomes included perioperative metrics, Macnab outcome, TCM syndrome score, radiographic disc height, spinal canal volume, length of hospital stay, surgical cost, and total hospitalization cost. The TLIF cohort was analyzed descriptively only.

### 2.8 Safety assessment

Adverse events were ascertained from inpatient records, nursing notes, laboratory tests, discharge summaries, and follow-up records. Events were graded using the Clavien-Dindo classification (Dindo et al., 2004). Because MYT contained Aconiti Radix Cocta, predefined adverse events of special interest were selected based on the known toxicological profile of aconite-containing preparations and included cardiovascular symptoms, arrhythmia, hypotension, perioral or limb numbness, dizziness, tremor, gastrointestinal intolerance, allergic reactions, hepatic or renal dysfunction, readmission, and reoperation (Chan, 2009; Singhuber et al., 2009; Zhou et al., 2015). Temporary interruption or discontinuation of MYT was considered when clinically significant intolerance, suspected herb-related adverse reactions, severe postoperative complications, or physician judgment indicated that continued administration was inappropriate.

### 2.9 Covariates for propensity weighting

To reduce measured confounding in the primary UBE-based comparison, the propensity score model included variables available before MYT exposure and plausibly related to both postoperative MYT use and postoperative outcomes. These covariates were age, sex, herniation level, baseline VAS score, baseline JOA score, baseline TCM syndrome score, baseline CRP, baseline D-dimer, baseline IL-6, preoperative disc height, and preoperative spinal canal volume. Perioperative variables such as operative time, blood loss, length of stay, and cost were not included in the propensity score model because they may lie on the causal pathway and were analyzed as outcomes.

### 2.10 Statistical analysis

All statistical analyses were performed using SPSS 26.0 and R 4.1.2. Normality of raw continuous variables was assessed using the Shapiro-Wilk test together with visual inspection of distribution plots. Raw continuous variables with approximately normal distributions are presented as mean +/- standard deviation, whereas non-normally distributed variables are summarized as median with interquartile range. Categorical variables are presented as counts and percentages.

Because treatment allocation was not randomized, stabilized IPTW based on propensity scores was used for the primary comparison between the two UBE cohorts (Austin and Stuart, 2015; Austin, 2011; Austin, 2009; Li et al., 2018; Rosenbaum and Rubin, 1983; Cole and Hernan, 2008; Hernan and Robins, 2020).

For repeated postoperative outcomes, IPTW-weighted generalized estimating equations with robust standard errors were used to evaluate between-group differences over time. For single-time-point continuous outcomes, weighted linear regression models with robust standard errors were applied. Adjusted continuous outcomes are reported as IPTW-adjusted model-estimated marginal means with robust standard errors or 95% confidence intervals, as appropriate.

Given the multiple postoperative outcomes and time points, the primary emphasis was placed on the main early recovery outcomes, whereas secondary and exploratory analyses were interpreted with caution. Sensitivity analyses using alternative adjustment strategies, including weight trimming at the 1st and 99th percentiles, overlap weighting, 1:1 propensity-score matching, and multivariable regression adjustment, were performed where feasible and are summarized in Supplementary Table S8. All statistical tests were two-sided. Statistical significance was assessed at P < 0.05, but clinical interpretation also considered effect size, temporal consistency, minimal clinically important difference thresholds (Ostelo et al., 2008; Copay et al., 2007), and the exploratory nature of secondary outcomes.

## 3 Results

### 3.1 Study flow and baseline characteristics

**FIGURE 1:**
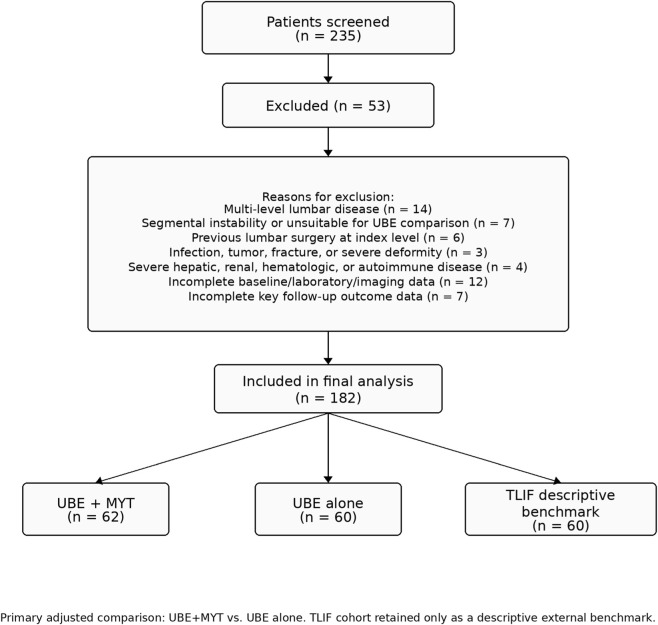
Patient selection and cohort assignment.

During the study period, 235 potentially eligible patients with symptomatic single-level LDH scheduled for surgical treatment were screened. Fifty-three patients were excluded after eligibility review. The reasons for exclusion were multi-level lumbar disease (n = 14), segmental instability requiring fixation or conditions unsuitable for the primary UBE comparison (n = 7), previous lumbar surgery at the index level (n = 6), spinal infection, tumor, fracture, or severe deformity (n = 3), severe hepatic, renal, hematologic, or autoimmune disease (n = 4), incomplete key baseline, laboratory, or imaging data (n = 12), and incomplete key follow-up outcome data (n = 7). Finally, 182 patients were included in the analysis: 62 in the UBE + MYT cohort, 60 in the UBE-alone cohort, and 60 in the TLIF descriptive benchmark cohort. The patient-selection flow and cohort assignment are shown in Figure 1.

Baseline and perioperative characteristics are summarized in Table 1. In the unweighted full cohort, the two UBE groups were broadly comparable with respect to age, sex distribution, herniation level, and baseline clinical severity, including preoperative VAS, JOA, TCM syndrome score, CRP, D-dimer, IL-6, disc height, and spinal canal volume. Across the full three-group comparison, the most substantial differences were driven by the TLIF cohort, which showed poorer baseline functional and structural status. Perioperatively, both UBE cohorts were associated descriptively with shorter operative time, lower blood loss, shorter hospital stay, and lower costs than the TLIF cohort. These between-procedure differences were interpreted descriptively because of likely indication-related baseline differences.

**TABLE 1:** Baseline characteristics of the UBE analytic cohort before and after inverse probability of treatment weighting (IPTW).

Variable	UBE alone before IPTW	UBE + MYT before IPTW	Absolute SMD before	UBE alone after IPTW	UBE + MYT after IPTW	Absolute SMD after
Age (years)	58.97 ± 14.85	59.89 ± 15.08	0.061	59.31 ± 14.77	59.35 ± 15.64	0.002
Male sex, n (%)	33 (55.0%)	30 (48.4%)	0.133	51.7%	51.0%	0.014
Preoperative VAS	7.53 ± 0.77	7.44 ± 0.76	0.128	7.49 ± 0.76	7.49 ± 0.75	0.003
Preoperative JOA	13.05 ± 1.90	12.85 ± 1.57	0.112	12.94 ± 2.22	12.92 ± 1.32	0.011
Preoperative TCM syndrome score	21.93 ± 1.31	22.06 ± 1.62	0.089	21.97 ± 1.26	21.96 ± 1.60	0.005
Preoperative CRP (mg/L)	4.32 ± 2.11	4.10 ± 1.82	0.113	4.17 ± 2.04	4.15 ± 1.80	0.007
Preoperative D-dimer (μg/mL)	0.37 ± 0.12	0.36 ± 0.13	0.015	0.37 ± 0.12	0.37 ± 0.12	0.011
Preoperative IL-6 (pg/mL)	27.00 ± 4.28	26.98 ± 2.13	0.005	26.95 ± 4.06	26.94 ± 2.08	0.004
Preoperative disc height (mm)	8.66 ± 0.58	8.86 ± 0.70	0.315	8.74 ± 0.58	8.75 ± 0.69	0.008
Preoperative spinal canal volume	96.72 ± 10.56	95.06 ± 10.38	0.158	95.90 ± 10.48	96.05 ± 10.48	0.014
Herniation level: Upper lumbar, n (%)	6 (10.0%)	5 (8.1%)	0.068	9.7%	9.5%	0.006
Herniation level: L4/5, n (%)	38 (63.3%)	35 (56.5%)	0.141	58.3%	59.0%	0.014
Herniation level: L5/S1, n (%)	16 (26.7%)	22 (35.5%)	0.191	32.0%	31.5%	0.012

**FIGURE 2:**
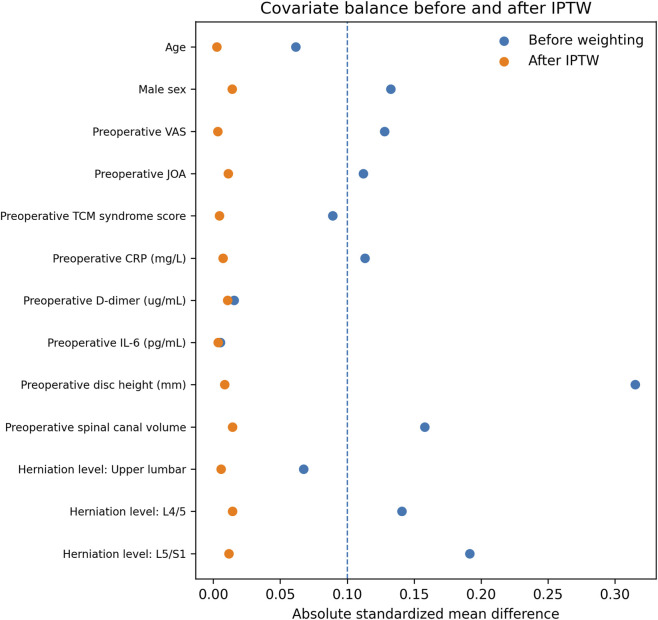
Covariate balance before and after stabilized IPTW in the UBE analytic cohort. Absolute standardized mean differences below 0.10 indicate acceptable balance.

In the primary UBE analytic cohort, IPTW substantially improved baseline comparability. Before weighting, the largest imbalance was observed for preoperative disc height. After weighting, all prespecified baseline covariates achieved acceptable balance, with all absolute SMDs below 0.10 (Table 1; Figure 2).

### 3.2 MYT administration and treatment-course verification

In the UBE + MYT cohort, MYT administration was verified using de-identified medical orders, pharmacy/decoction-room records, dispensing records, nursing administration records, and follow-up information. All MYT exposure occurred after surgery, and no patient received preoperative MYT. The intended course was one daily dose for 7 postoperative days, divided into morning and evening administrations. MYT was initiated on POD0 evening in 38 patients, POD1 morning in 21 patients, POD1 evening in 2 patients, and POD2 morning in 1 patient. Fifty-two patients completed all 14 planned administrations, whereas the remaining patients had minor omissions or early discontinuation documented in the source records. No patient was reclassified between cohorts after verification of MYT exposure. Details of MYT initiation timing and treatment-course adherence are provided in Supplementary Table S3.

### 3.3 IPTW diagnostics and sensitivity analyses

IPTW diagnostics showed adequate covariate balance and no evidence of problematic extreme weights. The maximum absolute SMD decreased from 0.315 before weighting to 0.014 after stabilized IPTW. The maximum stabilized weight was 2.064, and no weight exceeded 10. The effective sample size after weighting was 113.9 overall, 58.0 in the UBE + MYT cohort, and 55.9 in the UBE-alone cohort. Propensity-score overlap was acceptable, with common support from 0.276 to 0.762; four observations were outside the strict common-support interval but did not produce extreme weights. Sensitivity analyses using trimmed IPTW, overlap weighting, 1:1 propensity-score matching, and multivariable regression adjustment yielded directionally consistent estimates for the main early recovery outcomes (Supplementary Tables S7, S8; Supplementary Figures S2, S3).

### 3.4 IPTW-weighted clinical and biochemical outcomes in the primary UBE analytic cohort

In the primary UBE analytic cohort, IPTW-adjusted outcome comparisons showed lower early postoperative pain scores and inflammatory biomarker levels in the UBE + MYT cohort than in the UBE-alone cohort (Table 2). Adjusted postoperative outcomes are reported as IPTW-adjusted model-estimated marginal means rather than simple raw arithmetic means.

**TABLE 2:** IPTW-weighted clinical and biochemical outcomes in the UBE analytic cohort.

Outcome	Time point	UBE alone	UBE + MYT	Mean difference (MYT - control)	95% CI	P
VAS	Preoperative	7.49 ± 0.10	7.49 ± 0.10	0.00	−0.27 to 0.28	0.986
VAS	POD1	5.99 ± 0.10	5.69 ± 0.11	−0.30	−0.60 to 0.00	0.053
VAS	POD3	4.67 ± 0.09	3.88 ± 0.10	−0.79	−1.06 to −0.53	<0.001
VAS	POD7	3.73 ± 0.08	2.85 ± 0.11	−0.88	−1.14 to −0.62	<0.001
IL-6 (pg/mL)	Preoperative	26.95 ± 0.51	26.94 ± 0.27	−0.01	−1.14 to 1.11	0.984
IL-6 (pg/mL)	POD1	66.73 ± 0.55	56.27 ± 0.63	−10.46	−12.10 to −8.82	<0.001
IL-6 (pg/mL)	POD3	31.54 ± 0.69	20.03 ± 0.41	−11.52	−13.09 to −9.94	<0.001
IL-6 (pg/mL)	POD7	14.02 ± 0.33	8.40 ± 0.23	−5.62	−6.40 to −4.84	<0.001
CRP (mg/L)	Preoperative	4.17 ± 0.27	4.15 ± 0.24	−0.01	−0.71 to 0.69	0.968
CRP (mg/L)	POD1	32.93 ± 0.32	30.08 ± 0.23	−2.86	−3.63 to −2.08	<0.001
CRP (mg/L)	POD3	26.24 ± 0.55	20.91 ± 0.78	−5.33	−7.20 to −3.46	<0.001
CRP (mg/L)	POD7	13.87 ± 0.24	7.72 ± 0.21	−6.14	−6.76 to −5.53	<0.001
D-dimer (μg/mL)	Preoperative	0.37 ± 0.02	0.37 ± 0.02	−0.00	−0.04 to 0.04	0.953
D-dimer (μg/mL)	POD1	1.46 ± 0.06	1.43 ± 0.06	−0.03	−0.19 to 0.12	0.660
D-dimer (μg/mL)	POD3	3.66 ± 0.08	2.77 ± 0.08	−0.89	−1.11 to −0.67	<0.001
D-dimer (μg/mL)	POD7	2.00 ± 0.09	1.57 ± 0.05	−0.43	−0.63 to −0.22	<0.001
JOA	Preoperative	12.94 ± 0.35	12.92 ± 0.15	−0.02	−0.76 to 0.72	0.957
JOA	1 month	21.17 ± 0.14	22.77 ± 0.10	1.60	1.25 to 1.94	<0.001
JOA	3 months	23.70 ± 0.08	24.36 ± 0.10	0.65	0.40 to 0.91	<0.001
JOA	6 months	25.62 ± 0.08	26.67 ± 0.13	1.05	0.75 to 1.35	<0.001
JOA	12 months	27.27 ± 0.11	28.21 ± 0.09	0.94	0.65 to 1.23	<0.001

For pain recovery, the IPTW-adjusted model-estimated preoperative VAS score was identical between the two groups. Postoperatively, VAS scores decreased in both cohorts. At POD1, the adjusted VAS score was numerically lower in the UBE + MYT cohort than in the UBE-alone cohort, although the pointwise difference remained modest. Clear between-group separation was observed at POD3 and POD7.

A similar temporal pattern was observed for IL-6. Baseline IL-6 levels were nearly identical after weighting. Both groups showed a postoperative rise in IL-6 on POD1 followed by progressive decline over time, but IL-6 levels were consistently lower in the UBE + MYT cohort during the first postoperative week. CRP showed a directionally concordant pattern, with lower postoperative levels in the UBE + MYT cohort at POD1, POD3, and POD7. Baseline D-dimer levels were also similar between groups. No significant weighted difference was observed at POD1, whereas lower D-dimer levels were observed in the UBE + MYT cohort at POD3 and POD7.

**FIGURE 3:**
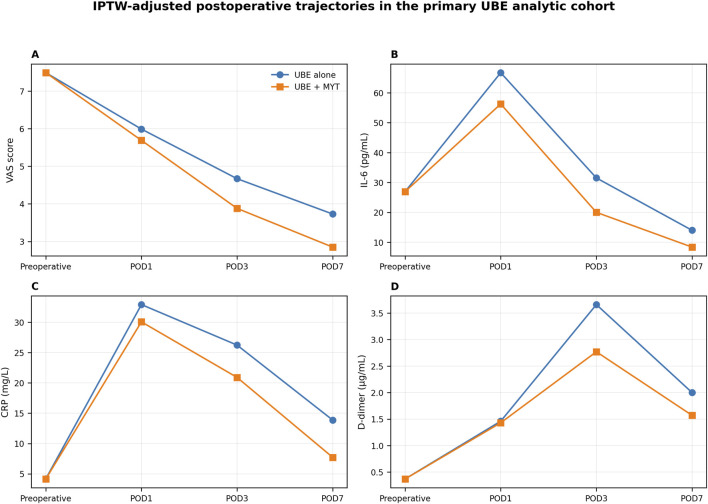
IPTW-adjusted postoperative trajectories of clinical and biochemical outcomes in the primary UBE analytic cohort. **(A)** VAS. **(B)** IL-6. **(C)** CRP. **(D)** D-dimer.

Functional recovery assessed by JOA scores also favored the UBE + MYT cohort over follow-up. After weighting, baseline JOA scores were essentially identical between groups, whereas follow-up JOA scores were higher in the UBE + MYT cohort at 1, 3, 6, and 12 months. These findings indicate that the early postoperative differences in pain and inflammatory biomarker trajectories were accompanied by better functional recovery during follow-up (Figure 3).

### 3.5 Perioperative management, laboratory sampling, and safety verification

Verification of perioperative co-interventions showed that both UBE cohorts followed the same institutional perioperative pathway except for MYT administration. Both cohorts were treated by the same senior spinal surgery team under general anesthesia and received the same surgical procedure, antibiotic prophylaxis, routine COX-2-based analgesic strategy, rescue analgesia criteria, rehabilitation guidance, discharge criteria, and scheduled laboratory monitoring (Supplementary Table S4; Supplementary Figure S4).

Routine early analgesia consisted of parecoxib sodium 40 mg intravenously every 12 h during POD0-POD2 or until oral transition, followed by celecoxib 200 mg orally twice daily when tolerated. Rescue analgesia with tramadol 50 mg orally or intramuscularly every 8 h as needed was available according to identical criteria. Rescue analgesia was used in 8 of 62 patients (12.9%) in the UBE + MYT cohort and 17 of 60 patients (28.3%) in the UBE-alone cohort. This rescue-analgesia finding was reported descriptively because rescue medication use may be influenced by postoperative pain severity, patient behavior, physician judgment, and nursing practice, and was not interpreted as independent evidence of an analgesic effect. Perioperative antibiotic prophylaxis and rehabilitation/discharge criteria were applied according to the same institutional pathway in both UBE cohorts.

Laboratory sampling for IL-6, CRP, and D-dimer followed a prespecified schedule before surgery and on POD1, POD3, and POD7, with morning blood sampling performed within a standardized window. Sampling completeness was 100% at all planned time points in both UBE cohorts (Supplementary Table S5).

### 3.6 Herbal preparation, traceability, and phytochemical characterization

To improve the reproducibility and transparency of the herbal intervention, MYT composition, pharmacopoeial identity, decoction procedure, and quality-control documentation were collated according to the ConPhyMP reporting framework. Patient-level administration records and pharmacy/decoction-room records were used to summarize clinical exposure. Batch-level manufacturer, inventory, certificate-of-analysis or inspection-report information for the clinically used batches is summarized in Supplementary Tables S1-S1C. In the UBE + MYT cohort, the clinically used batches of all seven MYT components were matched to available COA/inspection reports and pharmacy traceability records. For Aconiti Radix Cocta, the safety-critical component of the formula, all 62 patients were matched to documented batches covered by corresponding inspection reports. The interpretation of chemical profiling and marker selection followed the principle that quality-control and safety-related markers support reproducibility and exposure documentation, not direct proof of clinical pharmacodynamic activity (Heinrich et al., 2022; Heinrich et al., 2020; Li et al., 2008). Source COA/inspection reports, manufacturer license documents, and pharmacy traceability records are provided in Supplementary Data Sheets 2-4.

UHPLC-Q-Orbitrap HRMS-based phytochemical characterization was performed to support chemical profiling of the MYT preparation and putative *in vivo* exposure documentation. Annotated constituents in the MYT sample and putative MYT-related constituents or metabolites detected in medicated serum are provided in Supplementary Tables S9, S10, and representative base peak ion chromatograms are shown in Supplementary Figure S1. These analyses were used to support chemical characterization and exposure documentation and were not interpreted as direct proof of pharmacodynamic mechanisms.

### 3.7 Safety outcomes

Postoperative adverse events were less frequent descriptively in the UBE + MYT cohort than in the UBE-alone cohort. In the UBE + MYT cohort, 2 of 62 patients experienced Clavien-Dindo grade I events, and no grade II or grade III or higher adverse events were observed. No readmission or reoperation was recorded in the UBE + MYT cohort.

Dedicated review of adverse events of special interest related to Aconiti Radix Cocta did not identify clear cardiovascular or neurologic toxicity signals, including arrhythmia, hypotension, perioral numbness, limb numbness, tremor, or syncope. No clinically significant hepatic or renal dysfunction considered related to MYT was identified in the available records. Because of the retrospective design and limited sample size, these findings should be interpreted as absence of an observed severe safety signal rather than definitive evidence of safety. Patient-level adverse-event adjudication and the safety summary are provided in Supplementary Table S6.

### 3.8 Descriptive external benchmark analysis of the TLIF cohort

The TLIF cohort was retained as an external benchmark to contextualize the UBE-based strategies, but it was not entered into the primary IPTW-adjusted comparison because of substantial baseline and indication-related differences. Patients in the TLIF cohort presented with poorer baseline functional and structural status than patients in either UBE cohort, consistent with a distinct and more severe surgical indication profile. The descriptive external benchmark comparison among the three cohorts is summarized in Table 3.

**TABLE 3:** Descriptive external benchmark comparison among the three cohorts.

Variable	UBE + MYT (n = 62)	UBE alone (n = 60)	TLIF (n = 60)
Operative time, min	117.50 (80.00, 158.75)	190.00 (160.00, 220.00)	220.00 (172.50, 261.25)
Intraoperative blood loss, mL	41.61 ± 13.54	43.53 ± 15.38	253.70 ± 19.22
Length of hospital stay, days	7.00 (6.00, 9.75)	10.00 (8.00, 12.50)	13.00 (10.00, 15.00)
Surgical cost	6129.70 (5700.82, 7774.25)	8057.45 (6815.57, 8381.92)	9037.45 (7852.40, 10961.15)
Total hospitalization cost	41980.46 (35941.79, 47445.94)	44834.88 (40935.29, 50097.15)	51510.02 (46429.32, 58553.88)
Preoperative JOA score	13.00 (12.00, 14.00)	13.00 (12.00, 14.00)	9.50 (9.00, 10.00)
JOA score at 12 months	28.00 (28.00, 29.00)	27.00 (27.00, 28.00)	26.00 (26.00, 26.25)
VAS score at POD7	3.00 (2.00, 3.00)	4.00 (3.00, 4.00)	5.00 (4.00, 5.00)
TCM syndrome score at 4 weeks	8.00 (8.00, 9.00)	12.00 (11.00, 12.00)	15.00 (15.00, 16.00)
Disc height at 12 months, mm	7.30 (7.10, 7.88)	7.40 (7.10, 7.62)	8.70 (8.50, 9.50)
Spinal canal volume at 3 months	180.00 (172.25, 185.00)	160.00 (153.00, 165.25)	168.50 (162.75, 174.25)
Macnab excellent/good at 1 month, n/N (%)	58/62 (93.5%)	52/60 (86.7%)	25/60 (41.7%)
Macnab excellent/good at 6 months, n/N (%)	62/62 (100.0%)	60/60 (100.0%)	51/60 (85.0%)
Macnab excellent/good at 12 months, n/N (%)	62/62 (100.0%)	60/60 (100.0%)	59/60 (98.3%)
Postoperative adverse events, n/N (%)	2/62 (3.2%)	9/60 (15.0%)	10/60 (16.7%)
- Clavien-Dindo grade I	2/62 (3.2%)	6/60 (10.0%)	6/60 (10.0%)
- Clavien-Dindo grade II	0/62 (0.0%)	3/60 (5.0%)	4/60 (6.7%)
- Clavien-Dindo grade ≥ III	0/62 (0.0%)	0/60 (0.0%)	0/60 (0.0%)

Perioperatively, the TLIF cohort showed a greater procedural burden than either UBE cohort, including longer operative time, greater blood loss, longer hospital stay, and higher treatment costs. Descriptive postoperative comparisons showed slower early recovery in the TLIF cohort. However, these findings should not be interpreted as evidence of comparative effectiveness because the TLIF cohort represented a clinically distinct population with different operative indications and surgical objectives. The external benchmark analysis is therefore contextual rather than causal.

## 4 Discussion

### 4.1 Principal findings

In this single-center retrospective comparative cohort study, postoperative MYT use was associated with a more favorable early postoperative recovery profile among patients undergoing UBE for LDH after adjustment for measured baseline differences using IPTW. Compared with UBE alone, the UBE + MYT cohort showed lower early postoperative pain scores, lower IL-6 and CRP levels during the first postoperative week, and better subsequent functional recovery. These associations were most apparent during the early postoperative phase.

Importantly, MYT was prepared through a unified hospital pharmacy/decoction-room procedure, and this study provides additional information on composition, pharmacopoeial identity, decoction process, treatment-course verification, available quality-control documentation, and safety monitoring. These additions improve the reproducibility and safety transparency of the herbal intervention.

### 4.2 Interpretation of pain and inflammatory outcomes

The parallel direction of early pain and inflammatory biomarker changes is a coherent finding of the present study. After weighting, baseline VAS and IL-6 values were essentially comparable between the two UBE cohorts, whereas postoperative separation emerged during the first week after surgery. However, causal mediation cannot be established in this retrospective analysis. Therefore, the concordant changes in pain and inflammatory biomarkers should be interpreted as a clinically coherent observational pattern rather than proof of a specific anti-inflammatory mechanism.

Although the adjusted between-group VAS differences reached statistical significance at POD3 and POD7, their absolute magnitude was modest and may be below commonly cited thresholds for clinically meaningful individual-level improvement in low back pain outcomes (Ostelo et al., 2008; Copay et al., 2007). Therefore, the pain findings should be interpreted as group-level evidence of faster early symptom recovery rather than definitive evidence of clinically meaningful analgesic superiority for every individual patient.

### 4.3 Functional recovery over follow-up

The early postoperative differences in pain and inflammatory biomarker levels were accompanied by higher JOA scores during follow-up in the UBE + MYT cohort. This pattern suggests that earlier postoperative recovery may be associated with smoother functional improvement. Nevertheless, late recovery after decompression is influenced by multiple factors beyond postoperative herbal administration, including preoperative disease burden, decompression adequacy, rehabilitation adherence, and the natural course of neural recovery. The follow-up functional results should therefore be interpreted as supportive observational evidence rather than proof that MYT alone determines long-term outcome.

### 4.4 Perioperative co-interventions and residual confounding

The additional perioperative verification reduces, but does not eliminate, concerns that analgesic, antibiotic, rehabilitation, or laboratory-sampling differences could explain the observed associations. Both UBE cohorts followed the same institutional pathway except for MYT, including the same surgical team, anesthesia approach, antibiotic prophylaxis, routine analgesic strategy, rescue analgesia criteria, rehabilitation guidance, discharge criteria, and laboratory sampling schedule. Rescue analgesia was used less frequently in the UBE + MYT cohort. This finding is directionally consistent with the observed lower early pain scores, but it should be interpreted descriptively because rescue analgesia may also be influenced by postoperative pain severity, patient behavior, physician judgment, nursing practice, and local analgesic protocols. Therefore, it was not interpreted as independent evidence of an analgesic effect of MYT.

### 4.5 MYT reproducibility, ConPhyMP reporting, and Aconiti Radix Cocta safety

This study provides expanded reporting of MYT preparation and traceability. The formula was administered as an institutional decoction prepared by the hospital pharmacy/decoction room rather than as a patient self-prepared herbal product. Pharmacopoeial identity, processing status, decoction procedure, and available batch-level quality-control documents were reviewed and summarized according to the ConPhyMP reporting framework (Heinrich et al., 2022).

Aconiti Radix Cocta was specifically verified as processed Zhi Chuan Wu and was not substituted by raw Chuan Wu (Chan, 2009; Singhuber et al., 2009; Zhou et al., 2015).

No grade III or higher adverse events and no clear cardiovascular or neurologic toxicity signal suggestive of aconite-related toxicity were observed in the UBE + MYT cohort. Nevertheless, the retrospective design and sample size limit the ability to evaluate rare herb-related adverse events. Therefore, the safety results should be regarded as reassuring but preliminary, and prospective studies with predefined safety monitoring are needed.

### 4.6 External benchmark interpretation of the TLIF cohort

A strength of the present study is the explicit restriction of the primary adjusted comparison to the two UBE cohorts, with the TLIF cohort retained only for descriptive contextualization. The TLIF cohort differed substantially from the UBE cohorts in baseline function, structural status, and operative indication. These differences strongly suggest confounding by indication and justify excluding TLIF from the primary IPTW-adjusted comparison.

Within this descriptive framework, the TLIF cohort provides clinical context regarding perioperative burden and recovery trajectory in a more invasive surgical population. However, the descriptive differences between TLIF and UBE-based strategies should not be interpreted as causal evidence that one operative approach is superior to another, because the procedures serve different surgical objectives and are applied to clinically different patients.

### 4.7 Strengths and limitations

This study has several strengths, including use of a primary UBE analytic cohort, adjustment for measured baseline confounding using IPTW, transparent separation of the TLIF cohort as a descriptive benchmark only, verification of MYT exposure using clinical and pharmacy records, standardized perioperative pathway verification, expanded reporting of MYT preparation according to ConPhyMP principles, and sensitivity analyses supporting the robustness of the main early recovery findings.

Several limitations should also be acknowledged. First, the retrospective single-center design precludes causal inference and leaves the possibility of residual confounding despite IPTW adjustment. Second, MYT use was not randomized, and the retrospective data could not fully reconstruct the clinical decision process by which patients received postoperative MYT in routine practice. Treatment selection may have been influenced by treating physician practice, patient preference, postoperative TCM assessment, oral tolerance, or time-period practice patterns. Therefore, residual confounding by indication and preference may remain despite IPTW adjustment. Third, multiple postoperative outcomes and time points were evaluated; therefore, secondary and exploratory findings should be interpreted cautiously. Fourth, the absolute VAS differences were modest and should be interpreted in relation to minimal clinically important difference thresholds. Fifth, although batch-level COA/inspection reports and hospital pharmacy traceability records were collated for the clinically used MYT components, the retrospective design did not include prospective batch-standardization, direct reference-standard chromatogram overlay, or chromatographic similarity scoring across multiple decoction batches. Rescue analgesia use was analyzed descriptively because it may be influenced by postoperative pain severity, patient behavior, physician judgment, and nursing practice. Therefore, the herbal quality-control evidence should be interpreted as clinical traceability and inspection-report documentation rather than full prospective pharmaceutical standardization. Sixth, the study was not powered to exclude rare herb-related adverse events, particularly those related to Aconiti Radix Cocta. Prospective multicenter studies with predefined herbal quality control, safety monitoring, and randomized or otherwise rigorously controlled designs are warranted.

## 5 Conclusion

In this retrospective comparative cohort study, postoperative MYT use was associated with lower early postoperative pain scores, lower inflammatory biomarker levels, and better subsequent functional recovery after UBE for lumbar disc herniation. MYT was prepared through a unified institutional decoction procedure, and no severe safety signal was observed in the UBE + MYT cohort. However, MYT exposure was not randomly assigned, and treatment-selection factors could not be fully reconstructed from retrospective records. These findings should therefore be interpreted as observational associations rather than causal evidence. Prospective validation with predefined herbal quality control, safety monitoring, and rigorous study design is required before broader clinical generalization.

## Data Availability

The original contributions presented in the study are included in the article and Supplementary Material. The de-identified patient-level clinical dataset is not publicly available because it contains sensitive clinical information and is subject to institutional privacy and ethics restrictions. Further inquiries can be directed to the corresponding authors.
