# Supplementary material for: Association of postoperative modified Yaotong Tang with early recovery after unilateral biportal endoscopy for lumbar disc herniation: a retrospective comparative cohort study using propensity score weighting
Source: Front Pharmacol. 2026 Jul 9;17:1852732. doi: 10.3389/fphar.2026.1852732 (PMC13391915; doi:10.3389/fphar.2026.1852732)
Supplement: Supplementary file 1 [file DataSheet2.pdf]

Supplementary Data Sheet 2. English index for COA and inspection reports

This English index maps the submitted Chinese batch inspection reports to the herbal components and patient-coverage ranges summarized in Supplementary Tables S1-S1C. The original Chinese source pages follow this index. Combined PDF page numbers include this one-page index.

Source page	Combined PDF page	Herb	Pharmacopoeial name	Batch No.	Manufacturer	COA/inspection report No.	Patient coverage	Conclusion/notes
20	21	Xu Duan / Chuan Duan	Dipsaci Radix	220112-1	Luzhou Baicaotang Chinese Herbal Decoction Pieces Co., Ltd.	C-22-011216	MYT-001-MYT-062	Compliant; source Chinese inspection report.
19	20	Fu Ling	Poria	220117-1	Luzhou Baicaotang Chinese Herbal Decoction Pieces Co., Ltd.	C-22-011713	MYT-001-MYT-062	Compliant; source Chinese inspection report.
18	19	Gan Jiang	Zingiberis Rhizoma	220310-1	Luzhou Baicaotang Chinese Herbal Decoction Pieces Co., Ltd.	C-22-031014	MYT-001-MYT-062	Compliant; source Chinese inspection report.
16	17	Bai Zhu	Atractylodis Macrocephalae Rhizoma	220124-2	Luzhou Baicaotang Chinese Herbal Decoction Pieces Co., Ltd.	C-22-012413	MYT-001-MYT-062	Compliant; source Chinese inspection report.
14	15	Zhi Gan Cao	Glycyrrhizae Radix et Rhizoma Praeparata cum Melle	220112-1	Luzhou Baicaotang Chinese Herbal Decoction Pieces Co., Ltd.	C-22-011216	MYT-001-MYT-062	Compliant; source Chinese inspection report.
17	18	Chen Pi	Citri Reticulatae Pericarpium	220119-1	Luzhou Baicaotang Chinese Herbal Decoction Pieces Co., Ltd.	C-22-011910	MYT-001-MYT-062	Compliant; source Chinese inspection report.
12	13	Zhi Chuan Wu	Aconiti Radix Cocta	211101	Sichuan Shengshang Da Health Pharmaceutical Co., Ltd.	JBCA06708211101	MYT-001-MYT-030	Compliant; includes diester-type alkaloid and content determination items.
10	11	Zhi Chuan Wu	Aconiti Radix Cocta	221026-1	Luzhou Baicaotang Chinese Herbal Decoction Pieces Co., Ltd.	C-22-102624	MYT-031-MYT-062	Compliant; includes diester-type alkaloid and content determination items.

泸州百草堂中药饮片有限公司
成品检验报告书

报告书编号:C-25-031514

品　名:	续断片	规　格:	片
产品批号:	250309-1	装　量:	kg/袋
物料代码:	C-125B	药材产地:	云南大理
代 表 量:	49.5kg	取样日期:	2025 年 03 月 13 日
检验日期:	2025 年 03 月 13 日	报告日期:	2025 年 03 月 15 日

检验依据:《中华人民共和国药典》2020 年版

检验项目	标准规定	检验结果
【性状】	应符合规定	符合规定
【鉴别】		
显微鉴别	应符合规定	符合规定
薄层鉴别 1	应检出续断对照药材斑点	检出续断对照药材斑点
薄层鉴别 2	应检出川续断皂苷VI对照品斑点	检出川续断皂苷VI对照品斑点
【检查】		
水分	不得过 10.0%	为 8.55%
总灰分	不得过 12.0%	为 6.59%
酸不溶性灰分	不得过 3.0%	为 1.2%
装量差异	应符合规定	符合规定
杂质	不得过 3.0%	为 1.1%
二氧化硫	不得过 150mg/kg	为 32.6mg/kg
【浸出物】	不得少于 45.0%	为 48.0%
【含量测定】	川续断皂苷VI($C_{47}H_{76}O_{18}$)不得少于 1.5%	为 7.1%

结论: 本品根据《中华人民共和国药典》2020 年版检验,结果符合规定。

质量受权人: 沈[illegible]　日　期: 2025 年 03 月 15 日

生产质量管理文件

泸州百草堂中药饮片有限公司
成品检验报告书

报告书编号:C-24-041217

品　名:	续断片	规　格:	片
批　号:	240412-1	包装规格:	/
物料代码:	C-125B	产　地:	云南
代 表 量:	49.9kg	取样日期:	2024 年 04 月 22 日
检验日期:	2024 年 04 月 22 日	报告日期:	2024 年 04 月 29 日

检验依据:《中华人民共和国药典》2020 年版

检验项目	标准规定	检验结果
【性状】	应符合规定	符合规定
【鉴别】		
显微鉴别	应符合规定	符合规定
薄层鉴别 1	应检出续断对照药材斑点	检出续断对照药材斑点
薄层鉴别 2	应检出川续断皂苷VI对照品斑点	检出川续断皂苷VI对照品斑点
【检查】		
水分	不得过 10.0%	为 6.31%
总灰分	不得过 12.0%	为 8.78%
酸不溶性灰分	不得过 3.0%	为 1.9%
装量差异	应符合规定	符合规定
杂质	不得过 3.0%	为 1.0%
二氧化硫	不得过 150mg/kg	为 36.2mg/kg
【浸出物】	不得少于 45.0%	为 45.8%
【含量测定】	川续断皂苷VI($C_{47}H_{76}O_{18}$)不得少于 1.5%	为 7.9%

结论: 本品根据《中华人民共和国药典》2020 年版检验,符合规定。

质量受权人: 梁英　日　期: 2024 年 04 月 29 日

生产质量管理文件

泸州百草堂中药饮片有限公司
成品检验报告书

报告书编号:C-23-042216

品　　名:	续断片	规　　格:	片
批　　号:	230422-1	包装规格:	kg/袋
物料代码:	C-125B	产　　地:	四川
代 表 量:	79.5kg	取样日期:	2023 年 04 月 27 日
检验日期:	2023 年 04 月 27 日	报告日期:	2023 年 05 月 02 日

检验依据:《中华人民共和国药典》2020 年版

检验项目	标准规定	检验结果
【性状】	应符合规定	符合规定
【鉴别】		
显微鉴别	应符合规定	符合规定
薄层鉴别 1	应检出续断对照药材斑点	检出续断对照药材斑点
薄层鉴别 2	应检出川续断皂苷Ⅵ对照品斑点	检出川续断皂苷Ⅵ对照品斑点
【检查】		
水分	不得过 10.0%	为 6.93%
总灰分	不得过 12.0%	为 7.25%
酸不溶性灰分	不得过 3.0%	为 1.9%
装量差异	应符合规定	符合规定
杂质	不得过 3.0%	为 1.3%
二氧化硫	不得过 150mg/kg	为 33.3mg/kg
【浸出物】	不得少于 45.0%	为 46.0%
【含量测定】	川续断皂苷Ⅵ($C_{47}H_{76}O_{18}$)不得少于 1.5%	为 8.9%

结论：本品根据《中华人民共和国药典》2020 年版检验,符合规定。

质量受权人：梁英　日期：2023 年 05 月 02 日

泸州百草堂中药饮片有限公司
成品检验报告书

报告书编号:C-23-011121

品　　名:	干姜	规　　格:	片
批　　号:	230111-1	包装规格:	kg/袋
物料代码:	C-319A	产　　地:	四川
代 表 量:	300kg	取样日期:	2023 年 01 月 14 日
检验日期:	2023 年 01 月 14 日	报告日期:	2023 年 01 月 16 日

检验依据:《中华人民共和国药典》2020 年版

检验项目	标准规定	检验结果
【性状】	本品呈不规则片状，气香，特异，味辛辣。	符合规定
【鉴别】		
显微鉴别	应符合规定	符合规定
薄层鉴别	应检出干姜对照药材、6-姜辣素对照品斑点	检出干姜对照药材、6-姜辣素对照品斑点
【检查】		
水分	不得过 19.0%	为 11.0%
总灰分	不得过 6.0%	为 4.2%
二氧化硫	不得过 150mg/kg	为 40.7mg/kg
杂质	不得过 3.0%	为 1.1%
装量差异	应符合规定	符合规定
【浸出物】	不得少于 22.0%	为 22.7%
【含量测定】	挥发油不得少于 0.8%(ml / g)	为 1.1%
	含 6-姜辣素($C_{17}H_{26}O_4$)不得少于 0.60%	为 0.96%

结论：本品根据《中华人民共和国药典》2020 年版检验，结果符合规定。

质量受权人：梁英　日期：2023 年 01 月 16 日

泸州百草堂中药饮片有限公司
成品检验报告书

报告书编号:C-24-011514

品　　名:	干姜	规　　格:	片
批　　号:	240115-1	包装规格:	kg/袋
物料代码:	C-319A	产　　地:	四川
代 表 量 :	149.25kg	取样日期:	2024 年 01 月 21 日
检验日期:	2024 年 01 月 21 日	报告日期:	2024 年 01 月 26 日

检验依据:《中华人民共和国药典》2020 年版

检验项目	标准规定	检验结果
【性状】	本品呈不规则片状，气香，特异，味辛辣。	符合规定
【鉴别】		
显微鉴别	应符合规定	符合规定
薄层鉴别	应检出干姜对照药材、6-姜辣素对照品斑点	检出干姜对照药材、6-姜辣素对照品斑点
【检查】		
水分	不得过 19.0%	为 11.4%
总灰分	不得过 6.0%	为 4.8%
二氧化硫	不得过 150mg/kg	为 29.9mg/kg
杂质	不得过 3.0%	为 0.9%
装量差异	应符合规定	符合规定
【浸出物】	不得少于 22.0%	为 22.4%
【含量测定】	挥发油不得少于 0.8%(ml / g)	为 1.1%
	含 6-姜辣素 ($C_{17}H_{26}O_4$) 不得少于 0.60%	为 0.62%

结论　本品根据《中华人民共和国药典》2020 年版检验，结果符合规定。

质量受权人：梁英　　日　期：2024 年 01 月 26 日

泸州百草堂中药饮片有限公司
成品检验报告书

报告书编号:C-25-012112

品　　名:	干姜	规　　格:	片
产品批号:	250114-1	装　　量:	kg/袋
物料代码:	C-319A	药材产地:	云南曲靖
代 表 量 :	236.5kg	取样日期:	2025 年 01 月 19 日
检验日期:	2025 年 01 月 19 日	报告日期:	2025 年 01 月 21 日

检验依据:《中华人民共和国药典》2020 年版

检验项目	标准规定	检验结果
【性状】	本品呈不规则片状，气香，特异，味辛辣。	符合规定
【鉴别】		
显微鉴别	应符合规定	符合规定
薄层鉴别	应检出干姜对照药材、6-姜辣素对照品斑点	检出干姜对照药材、6-姜辣素对照品斑点
【检查】		
水分	不得过 19.0%	为 9.14%
总灰分	不得过 6.0%	为 3.9%
二氧化硫	不得过 150mg/kg	为 42.7mg/kg
杂质	不得过 3.0%	为 1.2%
装量差异	应符合规定	符合规定
【浸出物】	不得少于 22.0%	为 25.1%
【含量测定】	挥发油不得少于 0.8%(ml / g)	为 1.88%
	含 6-姜辣素 ($C_{17}H_{26}O_4$) 不得少于 0.60%	为 0.86%

结论　本品根据《中华人民共和国药典》2020 年版检验，结果符合规定。

质量受权人：[illegible]　　日　期：2025 年 01 月 21 日

泸州百草堂中药饮片有限公司
成品检验报告书

报告书编号:C-23-010216

品　　名:	白术	规　　格:	片
批　　号:	230102-1	包装规格:	/
物料代码:	C-014B	产　　地:	安徽
代 表 量:	538.85kg	取样日期:	2023 年 01 月 08 日
检验日期:	2023 年 01 月 08 日	报告日期:	2023 年 01 月 12 日

检验依据:《中华人民共和国药典》2020 年版

检验项目	标准规定	检验结果
【性状】	应符合规定	符合规定
【鉴别】		
薄层鉴别	应检出白术对照药材斑点	检出白术对照药材斑点
【检查】		
水分	不得过 15.0%	为 8.53%
总灰分	不得过 5.0%	为 3.6%
色度	应符合规定	符合规定
杂质	不得过 3.0%	为 1.2%
二氧化硫	不得过 400mg/kg	为 124mg/kg
装量差异	应符合规定	符合规定
禁用农药多残留	不得检出（不得过定量限）	符合规定
【浸出物】	不得少于 35.0%	为 35.9%

结论: 本品根据《中华人民共和国药典》2020 年版检验,符合规定。

质量受权人: 梁英　　日 期: 2023 年 01 月 12 日

生产质量管理文件

泸州百草堂中药饮片有限公司
成品检验报告书

报告书编号:C-24-031116

品　　名:	白术	规　　格:	片
批　　号:	240311-1	包装规格:	kg/袋
物料代码:	C-014B	产　　地:	安徽
代 表 量:	99.5kg	取样日期:	2024 年 03 月 14 日
检验日期:	2024 年 03 月 14 日	报告日期:	2024 年 03 月 18 日

检验依据:《中华人民共和国药典》2020 年版

检验项目	标准规定	检验结果
【性状】	应符合规定	符合规定
【鉴别】		
薄层鉴别	应检出白术对照药材斑点	检出白术对照药材斑点
【检查】		
水分	不得过 15.0%	为 8.25%
总灰分	不得过 5.0%	为 3.1%
色度	应符合规定	符合规定
杂质	不得过 3.0%	为 1.1%
二氧化硫	不得过 400mg/kg	为 117mg/kg
装量差异	应符合规定	符合规定
禁用农药多残留	不得检出（不得过定量限）	符合规定
【浸出物】	不得少于 35.0%	为 36.3%

结论: 本品根据《中华人民共和国药典》2020 年版检验,符合规定。

质量受权人: 梁英　　日 期: 2024 年 03 月 18 日

生产质量管理文件

泸州百草堂中药饮片有限公司
成品检验报告书

报告书编号:C-25-081812

品 名:	白术	规 格:	片
产品批号:	250720-2	装 量:	kg/袋
物料代码:	C-014B	药材产地:	安徽亳州
代表量:	49.5kg	取样日期:	2025年08月14日
检验日期:	2025年08月14日	报告日期:	2025年08月18日

检验依据:《中华人民共和国药典》2020年版

检验项目	标准规定	检验结果
【性状】	应符合规定	符合规定
【鉴别】		
薄层鉴别	应检出白术对照药材斑点	检出白术对照药材斑点
【检查】		
水分	不得过15.0%	为12.3%
总灰分	不得过5.0%	为3.7%
色度	应符合规定	符合规定
杂质	不得过3.0%	为0.9%
二氧化硫	不得过400mg/kg	为48.7mg/kg
装量差异	应符合规定	符合规定
禁用农药多残留	不得检出（不得过定量限）	符合规定
【浸出物】	不得少于35.0%	为38.6%

结论：本品根据《中华人民共和国药典》2020年版检验，结果符合规定。

质量受权人：[illegible] 日 期：2025年08月18日

泸州百草堂中药饮片有限公司
成品检验报告书

报告书编号:C-23-011121

品 名:	茯苓	规 格:	块
批 号:	230111-1	包装规格:	kg/袋
物料代码:	C-182A	产 地:	云南
代表量:	491.25kg	取样日期:	2023年01月14日
检验日期:	2023年01月14日	报告日期:	2023年01月17日

检验依据:《中华人民共和国药典》2020年版

检验项目	标准规定	检验结果
【性状】	应符合规定	符合规定
【鉴别】		
显微鉴别	应符合规定	符合规定
理化鉴别	应符合规定	符合规定
薄层鉴别	应检出茯苓对照药材斑点	检出茯苓对照药材斑点
【检查】		
水分	不得过18.0%	为10.1%
总灰分	不得过2.0%	为1.2%
二氧化硫	不得过150mg/kg	为38.4mg/kg
杂质	不得过3.0%	为1.2%
装量差异	应符合规定	符合规定
【浸出物】		
醇溶性浸出物	不得少于2.5%	为3.2%

结论：本品根据《中华人民共和国药典》2020年版检验,符合规定。

质量受权人：梁英 日期：2023年01月17日

泸州百草堂中药饮片有限公司
成品检验报告书

报告书编号:C-24-030319

品　　名:	茯苓	规　　格:	块
批　　号:	240303-1	包装规格:	kg/袋
物料代码:	C-182A	产　　地:	云南
代 表 量:	1013kg	取样日期:	2024 年 03 月 13 日
检验日期:	2024 年 03 月 13 日	报告日期:	2024 年 03 月 18 日

检验依据:《中华人民共和国药典》2020 年版

检验项目	标准规定	检验结果
【性状】	应符合规定	符合规定
【鉴别】		
显微鉴别	应符合规定	符合规定
理化鉴别	应符合规定	符合规定
薄层鉴别	应检出茯苓对照药材斑点	检出茯苓对照药材斑点
【检查】		
水分	不得过 18.0%	为 10.5%
总灰分	不得过 2.0%	为 1.3%
二氧化硫	不得过 150mg/kg	为 45.6mg/kg
杂质	不得过 3.0%	为 1.0%
装量差异	应符合规定	符合规定
【浸出物】		
醇溶性浸出物	不得少于 2.5%	为 3.0%

结论: 本品根据《中华人民共和国药典》2020 年版检验,符合规定。

泸州百草堂中药饮片有限公司 质检专用章

质量受权人: 梁英　日　期: 2024 年 03 月 18 日

泸州百草堂中药饮片有限公司
成品检验报告书

报告书编号:C-25-041906

品　　名:	茯苓	规　　格:	块
产品批号:	250225-3	装　　量:	kg/袋
物料代码:	C-182A	药材产地:	云南普洱
代 表 量:	199.5kg	取样日期:	2025 年 04 月 14 日
检验日期:	2025 年 04 月 14 日	报告日期:	2025 年 04 月 19 日

检验依据:《中华人民共和国药典》2020 年版及第一增补本

检验项目	标准规定	检验结果
【性状】	应符合规定	符合规定
【鉴别】		
显微鉴别	应符合规定	符合规定
理化鉴别	应符合规定	符合规定
薄层鉴别	应检出茯苓对照药材斑点	检出茯苓对照药材斑点
【检查】		
水分	不得过 15.0%	为 14.9%
总灰分	不得过 2.0%	为 0.8%
二氧化硫	不得过 150mg/kg	为 43.6mg/kg
杂质	不得过 3.0%	为 0.9%
装量差异	应符合规定	符合规定
【浸出物】		
醇溶性浸出物	不得少于 2.5%	为 3.4%

结论: 本品根据《中华人民共和国药典》2020 年版及第一增补本检验，结果符合规定。

泸州百草堂中药饮片有限公司 质检专用章

质量受权人: [illegible]　日　期: 2025 年 04 月 19 日

泸州百草堂中药饮片有限公司
成品检验报告书

报告书编号:C-25-041906

品　　名:	茯苓	规　　格:	块
产品批号:	250225-3	装　　量:	kg/袋
物料代码:	C-182A	药材产地:	云南普洱
代 表 量:	199.5kg	取样日期:	2025 年 04 月 14 日
检验日期:	2025 年 04 月 14 日	报告日期:	2025 年 04 月 19 日

检验依据:《中华人民共和国药典》2020 年版及第一增补本

检验项目	标准规定	检验结果
【性状】	应符合规定	符合规定
【鉴别】		
显微鉴别	应符合规定	符合规定
理化鉴别	应符合规定	符合规定
薄层鉴别	应检出茯苓对照药材斑点	检出茯苓对照药材斑点
【检查】		
水分	不得过 15.0%	为 14.9%
总灰分	不得过 2.0%	为 0.8%
二氧化硫	不得过 150mg/kg	为 43.6mg/kg
杂质	不得过 3.0%	为 0.9%
装量差异	应符合规定	符合规定
【浸出物】		
醇溶性浸出物	不得少于 2.5%	为 3.4%

结论: 本品根据《中华人民共和国药典》2020 年版及第一增补本检验，结果符合规定。

质量受权人: [illegible] 日期: 2025 年 04 月 19 日

泸州百草堂中药饮片有限公司 质检专用章

泸州百草堂中药饮片有限公司
成品检验报告书

报告书编号:C-23-032519

品　　名:	炙甘草	规　　格:	蜜炙
批　　号:	230325-1	包装规格:	/
物料代码:	C-144R	产　　地:	甘肃
代 表 量:	534.2kg	取样日期:	2023 年 04 月 03 日
检验日期:	2023 年 04 月 03 日	报告日期:	2023 年 04 月 05 日

检验依据:《中华人民共和国药典》2020 年版

检验项目	标准规定	检验结果
【性状】	应符合规定	符合规定
【鉴别】		
薄层鉴别	应检出甘草对照药材、甘草酸单铵盐对照品斑点	检出甘草对照药材、甘草酸单铵盐对照斑点
【检查】		
水分	不得过 10.0%	为 6.11%
总灰分	不得过 5.0%	为 4.7%
装量差异	应符合规定	符合规定
二氧化硫	不得过 150mg/kg	为 45.7mg/kg
杂质	不得过 3.0%	为 1.0%
禁用农药多残留	不得检出（不得过定量限）	符合规定
【含量测定】		
	含甘草苷（C21H22O9）不得少于 0.50%	为 0.58%
	甘草酸（C42H62O16）不得少于 1.0%	为 2.3%

结论: 本品根据《中华人民共和国药典》2020 年版检验，结果符合规定。

质量受权人: 梁英 日期: 2023 年 04 月 05 日

泸州百草堂中药饮片有限公司 质检专用章

泸州百草堂中药饮片有限公司
成品检验报告书

报告书编号:C-24-011804

品　名:	炙甘草	规　格:	蜜炙
批　号:	240118-1	包装规格:	/
物料代码:	C-144R	产　地:	甘肃
代 表 量:	780.15kg	取样日期:	2024 年 01 月 23 日
检验日期:	2024 年 01 月 23 日	报告日期:	2024 年 01 月 25 日

检验依据:《中华人民共和国药典》2020 年版

检验项目	标准规定	检验结果
【性状】	应符合规定	符合规定
【鉴别】		
薄层鉴别	应检出甘草对照药材、甘草酸单铵盐对照品斑点	检出甘草对照药材、甘草酸单铵盐对照斑点
【检查】		
水分	不得过 10.0%	为 5.12%
总灰分	不得过 5.0%	为 3.6%
装量差异	应符合规定	符合规定
二氧化硫	不得过 150mg/kg	为 43.1mg/kg
杂质	不得过 3.0%	为 1.0%
禁用农药多残留	不得检出（不得过定量限）	符合规定
【含量测定】		
	含甘草苷（C21H22O9）不得少于 0.50%	为 0.77%
	甘草酸（C42H62O16）不得少于 1.0%	为 2.1%

结论: 本品根据《中华人民共和国药典》2020 年版检验，结果符合规定。

泸州百草堂中药饮片有限公司 质检专用章

质量受权人: 梁英　日 期: 2024 年 01 月 25 日

泸州百草堂中药饮片有限公司
成品检验报告书

报告书编号:C-25-071518

品　名:	炙甘草	规　格:	蜜炙
产品批号:	250407-1	装　量:	/
物料代码:	C-144R	药材产地:	甘肃定西
代 表 量:	51.6kg	取样日期:	2025 年 07 月 12 日
检验日期:	2025 年 07 月 12 日	报告日期:	2025 年 07 月 15 日

检验依据:《中华人民共和国药典》2020 年版

检验项目	标准规定	检验结果
【性状】	应符合规定	符合规定
【鉴别】		
薄层鉴别	应检出甘草对照药材、甘草酸单铵盐对照品斑点	检出甘草对照药材、甘草酸单铵盐对照斑点
【检查】		
水分	不得过 10.0%	为 7.55%
总灰分	不得过 5.0%	为 3.3%
装量差异	应符合规定	符合规定
二氧化硫	不得过 150mg/kg	为 44.2mg/kg
杂质	不得过 3.0%	为 0.6%
禁用农药多残留	不得检出（不得过定量限）	符合规定
【含量测定】		
	含甘草苷（C21H22O9）不得少于 0.50%	为 0.65%
	甘草酸（C42H62O16）不得少于 1.0%	为 2.0%

结论: 本品根据《中华人民共和国药典》2020 年版检验，结果符合规定。

泸州百草堂中药饮片有限公司 质检专用章

质量受权人: [illegible]　日 期: 2025 年 07 月 15 日

泸州百草堂中药饮片有限公司
成品检验报告书

报告书编号:C-23-111917

品　名:	陈皮	规　格:	丝
批　号:	231119-1	包装规格:	kg/袋
物料代码:	C-300B	产　地:	四川
代表量:	101.5kg	取样日期:	2023 年 11 月 24 日
检验日期:	2023 年 11 月 24 日	报告日期:	2023 年 11 月 27 日

检验依据:《中华人民共和国药典》2020 年版

检验项目	标准规定	检验结果
【性状】	应符合规定	符合规定
【鉴别】		
显微鉴别	应符合规定	符合规定
薄层鉴别	应显橙皮苷对照品斑点	显橙皮苷对照品斑点
【检查】		
水分	不得过 13.0%	为 6.28%
二氧化硫	不得过 150mg/kg	为 43.4mg/kg
杂质	不得过 3.0%	为 1.1%
黄曲霉毒素	应符合规定	符合规定
禁用农药多残留	不得检出（不得过定量限）	符合规定
装量差异	应符合规定	符合规定
【含量测定】		
橙皮苷($C_{28}H_{34}O_{15}$)	不得少于 2.5%	为 5.4%

结论 本品根据《中华人民共和国药典》2020 年版检验，结果符合规定。

质量受权人： 梁英　日 期：2023 年 11 月 27 日

泸州百草堂中药饮片有限公司
成品检验报告书

报告书编号:C-24-032107

品　名:	陈皮	规　格:	丝
批　号:	240321-1	包装规格:	kg/袋
物料代码:	C-300B	产　地:	四川
代表量:	346kg	取样日期:	2024 年 03 月 27 日
检验日期:	2024 年 03 月 27 日	报告日期:	2024 年 03 月 30 日

检验依据:《中华人民共和国药典》2020 年版

检验项目	标准规定	检验结果
【性状】	应符合规定	符合规定
【鉴别】		
显微鉴别	应符合规定	符合规定
薄层鉴别	应显橙皮苷对照品斑点	显橙皮苷对照品斑点
【检查】		
水分	不得过 13.0%	为 8.19%
二氧化硫	不得过 150mg/kg	为 57.6mg/kg
杂质	不得过 3.0%	为 0.9%
黄曲霉毒素	应符合规定	符合规定
禁用农药多残留	不得检出（不得过定量限）	符合规定
装量差异	应符合规定	符合规定
【含量测定】		
橙皮苷($C_{28}H_{34}O_{15}$)	不得少于 2.5%	为 3.6%

结论 本品根据《中华人民共和国药典》2020 年版检验，结果符合规定。

质量受权人： 梁英　日 期：2024 年 03 月 30 日

泸州百草堂中药饮片有限公司
成品检验报告书

报告书编号:C-25-052711

品　名:	陈皮	规　格:	丝
产品批号:	250515-1	装　量:	kg/袋
物料代码:	C-300B	药材产地:	四川自贡
代 表 量:	249kg	取样日期:	2025 年 05 月 24 日
检验日期:	2025 年 05 月 24 日	报告日期:	2025 年 05 月 27 日

检验依据:《中华人民共和国药典》2020 年版

检验项目	标准规定	检验结果
【性状】	应符合规定	符合规定
【鉴别】		
显微鉴别	应符合规定	符合规定
薄层鉴别	应显橙皮苷对照品斑点	显橙皮苷对照品斑点
【检查】		
水分	不得过 13.0%	为 11.3%
二氧化硫	不得过 150mg/kg	为 48.2mg/kg
杂质	不得过 3.0%	为 0.6%
黄曲霉毒素	应符合规定	符合规定
禁用农药多残留	不得检出（不得过定量限）	符合规定
装量差异	应符合规定	符合规定
【含量测定】		
	橙皮苷($C_{28}H_{34}O_{15}$)不得少于 2.5%	为 3.9%

结论　本品根据《中华人民共和国药典》2020 年版检验，结果符合规定。

质量受权人: [illegible]　日　期: 2025 年 05 月 27 日

泸州百草堂中药饮片有限公司
成品检验报告书

报告书编号:C-22-102624

品　名:	制川乌	规　格:	煮制
批　号:	221026-1	包装规格:	kg/袋
物料代码:	C-142J	产　地:	四川
代 表 量:	38.5kg	取样日期:	2023 年 06 月 17 日
检验日期:	2023 年 06 月 17 日	报告日期:	2023 年 06 月 21 日

检验依据:《中华人民共和国药典》2020 年版

检验项目	标准规定	检验结果
【性状】	应符合规定	符合规定
【鉴别】		
薄层鉴别	应检查苯甲酰乌头原碱对照品、苯甲酰次乌头原碱对照品及苯甲酰新乌头原碱对照品斑点	检查苯甲酰乌头原碱对照品、苯甲酰次乌头原碱对照品及苯甲酰新乌头原碱对照品斑点
【检查】		
水分	不得过 11.0%	为 7.08%
二氧化硫	不得过 150mg/kg	为 43.4mg/kg
杂质	不得过 3.0%	为 1.2%
装量差异	应符合规定	符合规定
禁用农药多残留	不得检出（不得过限量）	符合规定
双酯型生物碱	含双酯型生物碱以乌头碱（C34H47NO11）、次乌头碱（C33H45NO10）及新乌头碱（C33H45NO11）的总量计，不得过 0.040%	为 0.002%
【含量测定】	含苯甲酰乌头原碱（C32H45NO10），苯甲酰次乌头原碱（C31H43NO9）及苯甲酰新乌头原碱（C31H43NO10）的总量应为 0.070%～0.15%	为 0.082%

结论　本品根据《中华人民共和国药典》2020 年版检验符合规定。

质量受权人: 梁英　日　期: 2023 年 06 月 21 日

泸州百草堂中药饮片有限公司
成品检验报告书

报告书编号:C-24-022815

品 名:	制川乌	规 格:	煮制
批 号:	240228-1	包装规格:	kg/袋
物料代码:	C-142J	产 地:	四川
代 表 量:	29.5kg	取样日期:	2024 年 03 月 06 日
检验日期:	2024 年 03 月 06 日	报告日期:	2024 年 03 月 08 日

检验依据:《中华人民共和国药典》2020 年版

检验项目	标准规定	检验结果
【性状】	应符合规定	符合规定
【鉴别】		
薄层鉴别	应检查苯甲酰乌头原碱对照品、苯甲酰次乌头原碱对照品及苯甲酰新乌头原碱对照品斑点	检查苯甲酰乌头原碱对照品、苯甲酰次乌头原碱对照品及苯甲酰新乌头原碱对照品斑点
【检查】		
水分	不得过 11.0%	为 6.98%
二氧化硫	不得过 150mg/kg	为 41.3mg/kg
杂质	不得过 3.0%	为 0.9%
装量差异	应符合规定	符合规定
禁用农药多残留	不得检出（不得过定量限）	符合规定
双酯型生物碱	含双酯型生物碱以乌头碱（C34H47NO11）、次乌头碱（C33H45NO10）及新乌头碱（C33H45NO11）的总量计，不得过 0.040%	为 0.005%
【含量测定】	含苯甲酰乌头原碱（C32H45NO10），苯甲酰次乌头原碱（C31H43NO9）及苯甲酰新乌头原碱（C31H43NO10）的总量应为 0.070%～0.15%	为 0.072%

结论：本品根据《中华人民共和国药典》2020 年版检验符合规定。

质量受权人： 梁英 日 期：2024 年 03 月 08 日

生产质量管理文件

泸州百草堂中药饮片有限公司
成品检验报告书

报告书编号:C-25-030809

品 名:	制川乌	规 格:	煮制
产品批号:	250113-1	装 量:	kg/袋
物料代码:	C-142J	药材产地:	四川绵阳
代 表 量:	19.5kg	取样日期:	2025 年 03 月 04 日
检验日期:	2025 年 03 月 04 日	报告日期:	2025 年 03 月 08 日

检验依据:《中华人民共和国药典》2020 年版

检验项目	标准规定	检验结果
【性状】	应符合规定	符合规定
【鉴别】		
薄层鉴别	应检查苯甲酰乌头原碱对照品、苯甲酰次乌头原碱对照品及苯甲酰新乌头原碱对照品斑点	检查苯甲酰乌头原碱对照品、苯甲酰次乌头原碱对照品及苯甲酰新乌头原碱对照品斑点
【检查】		
水分	不得过 11.0%	为 7.09%
二氧化硫	不得过 150mg/kg	为 46.6mg/kg
杂质	不得过 3.0%	为 0.6%
装量差异	应符合规定	符合规定
禁用农药多残留	不得检出（不得过定量限）	符合规定
双酯型生物碱	含双酯型生物碱以乌头碱（C34H47NO11）、次乌头碱（C33H45NO10）及新乌头碱（C33H45NO11）的总量计，不得过 0.040%	为 0.002%
【含量测定】	含苯甲酰乌头原碱（C32H45NO10），苯甲酰次乌头原碱（C31H43NO9）及苯甲酰新乌头原碱（C31H43NO10）的总量应为 0.070%～0.15%	为 0.090%

结论：本品根据《中华人民共和国药典》2020 年版检验，结果符合规定。

质量受权人： 日 期：2025 年 03 月 08 日

生产质量管理文件

RC-9045-01

四川圣上大健康药业有限公司

成品检验报告书

报告书编号：JBCA06708211101

品　　名	制川乌	批　　号	211101
规　　格	煮制	物料编码	CA06708
来　　源	生产车间	产　　地	四川
销 售 量	50.0kg	取样时间	20211129
包装规格	0.5kg	报告日期	20211211
检验依据	《中国药典》2020年版		

检验项目	标准规定	检验结果
装量差异	应符合规定	符合规定
包装外观	应完好无损	符合规定
【性　　状】	本品为不规则或长三角形的片。表面黑褐色或黄褐色，有灰棕色形成层环纹。体轻，质脆，断面有光泽。气微，微有麻舌感。	符合规定
【鉴　　别】	供试品色谱中，在与对照品色谱相应的位置上，应显相同颜色的斑点。	符合规定
【检　　查】		
杂　　质	应不得过3.0%	0.2%
水　　分	应不得过11.0%	9.8%
双酯型生物碱	本品按干燥品计算，含乌头碱、次乌头碱和新乌头碱的总量计，不得过0.040%。	0.007%
二氧化硫残留量	应不得过150mg/kg	符合规定
33种禁用农药	应符合规定	符合规定
【含量测定】	本品按干燥品计算，含苯甲酰乌头原碱，苯甲酰次乌头原碱及苯甲酰新乌头原碱的总量应为0.070%～0.15%。	0.13%

四川圣上大健康药业有限公司 质检专用章

检验结论：本品按《中国药典》2020年版检验，结果符合规定。

泸州百草堂中药饮片有限公司
成品检验报告书

报告书编号:C-22-011216

品　名:	甘草片	规　格:	片
批　号:	220112-1	包装规格:	kg/袋
物料代码:	C-0144	产　地:	甘肃
代表量:	148kg	取样日期:	2022 年 01 月 17 日
检验日期:	2022 年 01 月 17 日	报告日期:	2022 年 01 月 20 日

检验依据:《中华人民共和国药典》2020 年版

检验项目	标准规定	检验结果
【性状】	应符合规定	符合规定
【鉴别】		
显微鉴别	应符合规定	符合规定
薄层鉴别	应检出甘草对照药材、甘草酸单铵盐对照品斑点	检出甘草对照药材、甘草酸单铵盐对照品斑点
【检查】		
水分	不得过 12.0%	为 7.63%
总灰分	不得过 5.0%	为 2.9%
二氧化硫	不得过 150mg/kg	为 55.7mg/kg
杂质	不得过 3.0%	为 1.2%
装量差异	应符合规定	符合规定
重金属及有害元素	应符合规定	符合规定
禁用农药多残留	不得检出（不得过限量）	符合规定
【含量测定】	含甘草苷(C21H22O9)不得少于 0.45%	为 0.53%
	甘草酸(C42H62O16)不得少于 1.8%	为 2.1%

结论：本品根据《中华人民共和国药典》2020 年版检验，结果符合规定。

质量受权人：梁英　日　期：2022 年 01 月 20 日

泸州百草堂中药饮片有限公司 质检专用章

生产质量管理文件

CS 扫描全能王
3亿人都在用的扫描App

泸州百草堂中药饮片有限公司
成品检验报告书

报告书编号:C-22-011216

品　名:	甘草片	规　格:	片
批　号:	220112-1	包装规格:	kg/袋
物料代码:	C-0144	产　地:	甘肃
代表量:	148kg	取样日期:	2022 年 01 月 17 日
检验日期:	2022 年 01 月 17 日	报告日期:	2022 年 01 月 20 日

检验依据:《中华人民共和国药典》2020 年版

检验项目	标准规定	检验结果
【性状】	应符合规定	符合规定
【鉴别】		
显微鉴别	应符合规定	符合规定
薄层鉴别	应检出甘草对照药材、甘草酸单铵盐对照品斑点	检出甘草对照药材、甘草酸单铵盐对照品斑点
【检查】		
水分	不得过 12.0%	为 7.63%
总灰分	不得过 5.0%	为 2.9%
二氧化硫	不得过 150mg/kg	为 55.7mg/kg
杂质	不得过 3.0%	为 1.2%
装量差异	应符合规定	符合规定
重金属及有害元素	应符合规定	符合规定
禁用农药多残留	不得检出（不得过限量）	符合规定
【含量测定】	含甘草苷(C21H2209)不得少于 0.45%	为 0.53%
	甘草酸(C42H62016)不得少于 1.8%	为 2.1%

结论: 本品根据《中华人民共和国药典》2020 年版检验，结果符合规定。

质量受权人: 梁英　日 期: 2022 年 01 月 20 日

泸州百草堂中药饮片有限公司 质检专用章

生产质量管理文件

泸州百草堂中药饮片有限公司
成品检验报告书

报告书编号:C-22-011216

品　名:	甘草片	规　格:	片
批　号:	220112-1	包装规格:	kg/袋
物料代码:	C-0144	产　地:	甘肃
代表量:	148kg	取样日期:	2022 年 01 月 17 日
检验日期:	2022 年 01 月 17 日	报告日期:	2022 年 01 月 20 日

检验依据:《中华人民共和国药典》2020 年版

检验项目	标准规定	检验结果
【性状】	应符合规定	符合规定
【鉴别】		
显微鉴别	应符合规定	符合规定
薄层鉴别	应检出甘草对照药材、甘草酸单铵盐对照品斑点	检出甘草对照药材、甘草酸单铵盐对照品斑点
【检查】		
水分	不得过 12.0%	为 7.63%
总灰分	不得过 5.0%	为 2.9%
二氧化硫	不得过 150mg/kg	为 55.7mg/kg
杂质	不得过 3.0%	为 1.2%
装量差异	应符合规定	符合规定
重金属及有害元素	应符合规定	符合规定
禁用农药多残留	不得检出（不得过限量）	符合规定
【含量测定】	含甘草苷($C_{21}H_{22}O_{9}$)不得少于 0.45%	为 0.53%
	甘草酸($C_{42}H_{62}O_{16}$)不得少于 1.8%	为 2.1%

结论：本品根据《中华人民共和国药典》2020 年版检验，结果符合规定。

质量受权人：梁英　日　期：2022 年 01 月 20 日

质检专用章

扫描全能王
3亿人都在用的扫描App

泸州百草堂中药饮片有限公司
成品检验报告书

报告书编号:C-22-012413

品　　名:	白术	规　　格:	片
批　　号:	220124-2	包装规格:	10g/袋
物料代码:	C-0014	产　　地:	浙江
代表量:	95.4kg	取样日期:	2022 年 01 月 27 日
检验日期:	2022 年 01 月 27 日	报告日期:	2022 年 02 月 07 日

检验依据:《中华人民共和国药典》2020 年版

检验项目	标准规定	检验结果
【性状】	应符合规定	符合规定
【鉴别】		
薄层鉴别	应检出白术对照药材斑点	检出白术对照药材斑点
【检查】		
水分	不得过 15.0%	为 7.04%
总灰分	不得过 5.0%	为 2.3%
色度	应符合规定	符合规定
杂质	不得过 3.0%	为 1.4%
二氧化硫	不得过 400mg/kg	为 108mg/kg
禁用农药多残留	不得检出（不得过限量）	符合规定
装量差异	应符合规定	符合规定
【浸出物】	不得少于 35.0%	为 36.9%

结论: 本品根据《中华人民共和国药典》2020 年版检验,符合规定。

质量受权人: 梁英　　日　期: 2022 年 02 月 07 日

泸州百草堂中药饮片有限公司 质检专用章

泸州百草堂中药饮片有限公司
成品检验报告书

报告书编号:C-22-011910

品　名:	陈皮	规　格:	净制
批　号:	220119-1	包装规格:	kg/袋
物料代码:	C-0300	产　地:	四川
代表量:	288kg	取样日期:	2022年01月22日
检验日期:	2022年01月22日	报告日期:	2022年01月26日

检验依据:《中华人民共和国药典》2020年版

检验项目	标准规定	检验结果
【性状】	应符合规定	符合规定
【鉴别】		
显微鉴别	应符合规定	符合规定
薄层鉴别	应显橙皮苷对照品斑点	显橙皮苷对照品斑点
【检查】		
水分	不得过13.0%	为7.39%
二氧化硫	不得过150mg/kg	为37.7mg/kg
杂质	不得过3.0%	为1.2%
黄曲霉毒素	应符合规定	符合规定
禁用农药多残留	不得检出（不得过限量）	符合规定
装量差异	应符合规定	符合规定
【含量测定】		
	橙皮苷($C_{28}H_{34}O_{15}$)不得少于3.5%	为6.2%

结论：本品根据《中华人民共和国药典》2020年版检验，结果符合规定。

泸州百草堂中药饮片有限公司 质检专用章

质量受权人：梁英　日期：2022年01月26日

CS 扫描全能王
3亿人都在用的扫描App

泸州百草堂中药饮片有限公司
成品检验报告书

报告书编号:C-22-031014

品　　名:	干姜	规　　格:	片
批　　号:	220310-1	包装规格:	/
物料代码:	C-0319	产　　地:	四川
代 表 量:	45kg	取样日期:	2022 年 03 月 17 日
检验日期:	2022 年 03 月 17 日	报告日期:	2022 年 03 月 21 日

检验依据:《中华人民共和国药典》2020 年版

检验项目	标准规定	检验结果
【性状】	本品呈不规则片状，气香，特异，味辛辣。	符合规定
【鉴别】		
显微鉴别	应符合规定	符合规定
薄层鉴别	应检出干姜对照药材、6-姜辣素对照品斑点	检出干姜对照药材、6-姜辣素对照品斑点
【检查】		
水分	不得过 19.0%	为 10.0%
总灰分	不得过 6.0%	为 4.2%
二氧化硫	不得过 150mg/kg	为 40.3mg/kg
杂质	不得过 3.0%	为 1.0%
装量差异	应符合规定	符合规定
【浸出物】	不得少于 22.0%	为 22.8%
【含量测定】	挥发油不得少于 0.8%(ml / g)	为 1.2%
	含 6-姜辣素($C_{17}H_{26}O_4$)不得少于 0.60%	为 0.73%

结论: 本品根据《中华人民共和国药典》2020 年版检验，结果符合规定。

质量受权人: 梁英　　日期: 2022 年 03 月 21 日

泸州百草堂中药饮片有限公司 质检专用章

生产质量管理文件

扫描全能王 3亿人都在用的扫描App

泸州百草堂中药饮片有限公司
成品检验报告书

报告书编号:C-22-011713

品　　名:	茯苓	规　　格:	块
批　　号:	220117-1	包装规格:	kg/袋
物料代码:	C-0182	产　　地:	云南
代表量:	1995.5kg	取样日期:	2022年01月21日
检验日期:	2022年01月21日	报告日期:	2022年01月26日

检验依据:《中华人民共和国药典》2020年版

检验项目	标准规定	检验结果
【性状】	应符合规定	符合规定
【鉴别】		
显微鉴别	应符合规定	符合规定
理化鉴别	应符合规定	符合规定
薄层鉴别	应检出茯苓对照药材斑点	检出茯苓对照药材斑点
【检查】		
水分	不得过18.0%	为9.43%
总灰分	不得过2.0%	为1.2%
二氧化硫	不得过150mg/kg	为46.3mg/kg
杂质	不得过3.0%	为1.2%
装量差异	应符合规定	符合规定
【浸出物】		
醇溶性浸出物	不得少于2.5%	为3.7%

结论: 本品根据《中华人民共和国药典》2020年版检验,符合规定。

泸州百草堂中药饮片有限公司 质检专用章

质量受权人: 梁英 2022年01月26日

生产质量管理文件

CS 扫描全能王
3亿人都在用的扫描App

泸州百草堂中药饮片有限公司
成品检验报告书

报告书编号:C-22-063009

品　　名:	续断片	规　　格:	片
批　　号:	220630-1	包装规格:	/
物料代码:	C-125B	产　　地:	四川
代 表 量 :	327.2kg	取样日期:	2022 年 07 月 06 日
检验日期:	2022 年 07 月 06 日	报告日期:	2022 年 07 月 08 日

检验依据:《中华人民共和国药典》2020 年版

检验项目	标准规定	检验结果
【性状】	应符合规定	符合规定
【鉴别】		
显微鉴别	应符合规定	符合规定
薄层鉴别 1	应检出续断对照药材斑点	检出续断对照药材斑点
薄层鉴别 2	应检出川续断皂苷Ⅵ对照品斑点	检出川续断皂苷Ⅵ对照品斑点
【检查】		
水分	不得过 10.0%	为 6.79%
总灰分	不得过 12.0%	为 7.31%
酸不溶性灰分	不得过 3.0%	为 1.6%
装量差异	应符合规定	符合规定
杂质	不得过 3.0%	为 1.3%
二氧化硫	不得过 150mg/kg	为 33.3mg/kg
【浸出物】	不得少于 45.0%	为 45.8%
【含量测定】	川续断皂苷Ⅵ($C_{47}H_{76}O_{18}$)不得少于 1.5%	为 8.9%

结论: 本品根据《中华人民共和国药典》2020 年版检验,符合规定。

质量受权人: [illegible]　日 期: 202[illegible]年 07 月 08 日

生产质量管理文件

CS 扫描全能王
3亿人都在用的扫描App
