# Supplementary material for: Association of postoperative modified Yaotong Tang with early recovery after unilateral biportal endoscopy for lumbar disc herniation: a retrospective comparative cohort study using propensity score weighting
Source: Front Pharmacol. 2026 Jul 9;17:1852732. doi: 10.3389/fphar.2026.1852732 (PMC13391915; doi:10.3389/fphar.2026.1852732)
Supplement: Supplementary file 2 [file DataSheet4.pdf]

Supplementary Data Sheet 4. English index for hospital pharmacy traceability records

This English index summarizes the submitted Chinese hospital pharmacy inbound acceptance records. These pages support supply-chain traceability for the clinically used herbal decoction-piece batches. Identifiable patient information is not included in this index.

Source page	Combined PDF page	Record type	Primary visible item	Supplier/manufacturer shown in source	Purpose / notes
1	2	Hospital inbound acceptance sheet	Chuan Duan / Dipsaci Radix-related inbound record	Luzhou Baicaotang / hospital pharmacy source record	Supports pharmacy inventory traceability; source fields are in Chinese and should be verified by the pharmacy if used for item-level batch matching.
2	3	Hospital inbound acceptance sheet	Fu Ling / Poria-related inbound record	Sichuan / Luzhou pharmacy supply chain source record	Supports pharmacy inventory traceability.
3	4	Hospital inbound acceptance sheet	Gan Jiang / Zingiberis Rhizoma-related inbound record	Sichuan / Luzhou pharmacy supply chain source record	Supports pharmacy inventory traceability.
4	5	Hospital inbound acceptance sheet	Chen Pi / Citri Reticulatae Pericarpium-related inbound record	Sichuan / Luzhou pharmacy supply chain source record	Supports pharmacy inventory traceability.
5	6	Hospital inbound acceptance sheet	Bai Zhu / Atractylodis Macrocephalae Rhizoma-related inbound record	Sichuan / Luzhou pharmacy supply chain source record	Supports pharmacy inventory traceability.
6	7	Hospital inbound acceptance sheet	Herbal decoction-piece inbound record	Sichuan / Luzhou pharmacy supply chain source record	Supports pharmacy inventory traceability; author/pharmacy confirmation recommended for item-level details.

西南医科大学附属中医医院外购入库验收单

库房：中药库　　供货单位：泸州百草堂药品营销有限公司　　入库单号：RK2022000905

序号	药名	规格	单位	数量	采购价	采购金额	零售价	零售金额	生产企业	验收结论	批准文号	批号	有效期
1	续断	片	kg	1	80.0000	80.00	100.0000	100.00	泸州百草堂	合格		211203-3	20261202
合计						80.00		100.00					

摘要：水中

质量情况：合格　　验收结论：合格

填制人：宋德丽　　入库时间：2022.03.30 16:37　　验收人：韩才珍　　审核日期：2022.03.30 16:37

药品会计审核：　　审核日期　　药房主任审核：　　审核日期

采购员审核：　　审核日期　　保管员：黄江　　打印日期:2026-06-09 16:03:03

1、白色联附随货同行交审核付款　　2、红色联交药品会计

西南医科大学附属中医医院外购入库验收单

库房：中药库　　供货单位：四川禾一天然药业有限公司　　入库单号：RK2022000262

序号	药名	规格	单位	数量	采购价	采购金额	零售价	零售金额	生产企业	验收结论	批准文号	批号	有效期
1	茯苓	丁	kg	400	102.0000	40,800.00	127.5000	51,000.00	四川禾一天	合格		220124	20270123
合计						40,800.00		51,000.00					

摘要：该批饮片为精选中药饮片

质量情况：合格　　验收结论：合格

填制人：黄江　　入库时间：2022.01.26 10:57　　验收人：韩才珍　　审核日期：2022.01.27 10:57

药品会计审核：　　审核日期　　药房主任审核：　　审核日期

采购员审核：　　审核日期　　保管员：黄江　　打印日期:2026-06-09 16:03:28

1、白色联附随货同行交审核付款　　2、红色联交药品会计

西南医科大学附属中医医院外购入库验收单

库房：中药库　　供货单位：泸州宝光医药有限公司　　入库单号：RK2022000103

序号	药名	规格	单位	数量	采购价	采购金额	零售价	零售金额	生产企业	验收结论	批准文号	批号	有效期
1	干姜	片	kg	20	68.0000	1,360.00	85.0000	1,700.00	四川鑫仁泰	合格		211201	20261224
合计						1,360.00		1,700.00					

摘要：城北1

质量情况：合格　　验收结论：合格

填制人：黄江　　入库时间：2022.01.12 17:44　　验收人：韩才珍　　审核日期：2022.01.12 17:44

药品会计审核：　　审核日期　　药房主任审核：　　审核日期

采购员审核：　　审核日期　　保管员：黄江　　打印日期:2026-06-09 16:03:47

1、白色联附随货同行交审核付款　　2、红色联交药品会计

西南医科大学附属中医医院外购入库验收单

库房：中药库　　供货单位：四川明荷中药科技有限公司　　入库单号：RK2022000163

序号	药名	规格	单位	数量	采购价	采购金额	零售价	零售金额	生产企业	验收结论	批准文号	批号	有效期
1	陈皮	丝	kg	200	47.0000	9,400.00	58.7500	11,750.00	四川明荷中	合格		211116-03	20261115
合计						9,400.00		11,750.00					

摘要：该批饮片为精选中药饮片

质量情况：合格　　验收结论：合格

填制人：黄江　　入库时间：2022.01.19 10:27　　验收人：韩才珍　　审核日期：2022.01.19 15:08

药品会计审核：　　审核日期　　药房主任审核：　　审核日期

采购员审核：　　审核日期　　保管员：黄江　　打印日期:2026-06-09 16:04:05

1、白色联附随货同行交审核付款　　2、红色联交药品会计

西南医科大学附属中医医院外购入库验收单

库房：中药库　　供货单位：四川明荷中药科技有限公司　　入库单号：RK2022000231

序号	药名	规格	单位	数量	采购价	采购金额	零售价	零售金额	生产企业	验收结论	批准文号	批号	有效期
1	白术	生片	kg	192	89.0000	17,088.00	111.2500	21,360.00	四川明荷中	合格		211218-01	20261217
合计						17,088.00		21,360.00					

摘要：该批饮片为精选中药饮片

质量情况：合格　　验收结论：合格

填制人：黄江　　入库时间：2022.01.24 10:09　　验收人：韩才珍　　审核日期：2022.01.24 11:54

药品会计审核：　　审核日期　　药房主任审核：　　审核日期

采购员审核：　　审核日期　　保管员：黄江　　打印日期:2026-06-09 16:04:21

1、白色联附随货同行交审核付款　　2、红色联交药品会计

西南医科大学附属中医医院外购入库验收单

库房：中药库　　供货单位：四川麒源药业有限责任公司　　入库单号：RK2022000014

序号	药名	规格	单位	数量	采购价	采购金额	零售价	零售金额	生产企业	验收结论	批准文号	批号	有效期
1	甘草	中片	kg	200	70.0000	14,000.00	87.5000	17,500.00	四川麒源药	合格		211202	20261225
合计						14,000.00		17,500.00					

摘要：该批饮片为精选中药饮片

质量情况：合格　　验收结论：合格

填制人：黄江　　入库时间：2022.01.05 09:56　　验收人：韩才珍　　审核日期：2022.01.05 09:57

药品会计审核：　　审核日期　　药房主任审核：　　审核日期

采购员审核：　　审核日期　　保管员：黄江　　打印日期:2026-06-09 16:04:45

1、白色联附随货同行交审核付款　　2、红色联交药品会计
