# Supplementary material for: Association of postoperative modified Yaotong Tang with early recovery after unilateral biportal endoscopy for lumbar disc herniation: a retrospective comparative cohort study using propensity score weighting
Source: Front Pharmacol. 2026 Jul 9;17:1852732. doi: 10.3389/fphar.2026.1852732 (PMC13391915; doi:10.3389/fphar.2026.1852732)
Supplement: Supplementary file 3 [file Table1.docx]

**Supplementary Tables S1-S11**

*All tables are presented in English for journal submission. Original Chinese source documents are indexed in the supplementary data sheets and should be used only as supporting evidence.*

**Supplementary Table S1. Pharmacopoeial identity, manufacturer, batch information, and quality-control documentation of MYT components.**

| Component | Botanical source / family | Medicinal part / processing | Daily dose | Batch No. | Manufacturer | COA / inspection report No. | Evidence page | Validity | Conclusion | Patient coverage |
| --- | --- | --- | --- | --- | --- | --- | --- | --- | --- | --- |
| Dipsaci Radix | Dipsacus asper Wall. ex Henry [Caprifoliaceae] | Dried root; decoction piece | 24 g | 220112-1 | Luzhou Baicaotang Chinese Herbal Decoction Pieces Co., Ltd. | C-22-011216 | 20 | Valid through 2027 | Compliant | MYT-001–MYT-062 |
| Poria | Poria cocos (Schw.) Wolf [Polyporaceae] | Dried sclerotium; decoction piece | 15 g | 220117-1 | Luzhou Baicaotang Chinese Herbal Decoction Pieces Co., Ltd. | C-22-011713 | 19 | Valid through 2027 | Compliant | MYT-001–MYT-062 |
| Zingiberis Rhizoma | Zingiber officinale Roscoe [Zingiberaceae] | Dried rhizome; decoction piece | 15 g | 220310-1 | Luzhou Baicaotang Chinese Herbal Decoction Pieces Co., Ltd. | C-22-031014 | 18 | Valid through 2027 | Compliant | MYT-001–MYT-062 |
| Atractylodis Macrocephalae Rhizoma | Atractylodes macrocephala Koidz. [Asteraceae] | Dried rhizome; decoction piece | 20 g | 220124-2 | Luzhou Baicaotang Chinese Herbal Decoction Pieces Co., Ltd. | C-22-012413 | 16 | Valid through 2027 | Compliant | MYT-001–MYT-062 |
| Glycyrrhizae Radix et Rhizoma Praeparata cum Melle | Glycyrrhiza uralensis Fisch./G. inflata Bat./G. glabra L. [Fabaceae] | Root/rhizome; honey-processed decoction piece | 12 g | 220112-1 | Luzhou Baicaotang Chinese Herbal Decoction Pieces Co., Ltd. | C-22-011216 | 14 | Valid through 2027 | Compliant | MYT-001–MYT-062 |
| Citri Reticulatae Pericarpium | Citrus reticulata Blanco [Rutaceae] | Mature fruit peel; decoction piece | 20 g | 220119-1 | Luzhou Baicaotang Chinese Herbal Decoction Pieces Co., Ltd. | C-22-011910 | 17 | Valid through 2027 | Compliant | MYT-001–MYT-062 |
| Aconiti Radix Cocta | Aconitum carmichaelii Debeaux [Ranunculaceae] | Processed daughter root; pre-decocted separately | 9 g | 211101 | Sichuan Shengshang Da Health Pharmaceutical Co., Ltd. | JBCA06708211101 | 12 | 2021/2022 source record; see report | Compliant / in accordance with Chinese Pharmacopoeia | MYT-001-MYT-030 |
| Aconiti Radix Cocta | Aconitum carmichaelii Debeaux [Ranunculaceae] | Processed daughter root; pre-decocted separately | 9 g | 221026-1 | Luzhou Baicaotang Chinese Herbal Decoction Pieces Co., Ltd. | C-22-102624 | 10 | 2022/2027 source record; see report | Compliant / in accordance with Chinese Pharmacopoeia | MYT-031-MYT-062 |

*COA, certificate of analysis; MYT, Modified Yaotong Tang. Chinese source reports are provided in Supplementary Data Sheet 2.*

**Supplementary Table S1A. Patient-level matching summary of Aconiti Radix Cocta batches in the UBE+MYT cohort.**

| Patient range | First-administration window | Aconiti Radix Cocta batch No. | Manufacturer | COA / inspection report No. | Evidence page | Coverage status | Safety-specific traceability |
| --- | --- | --- | --- | --- | --- | --- | --- |
| MYT-001 to MYT-030 | POD0 evening to POD2 morning according to patient records | Batch 211101 | Sichuan Shengshang Da Health Pharmaceutical Co., Ltd. | JBCA06708211101 | 12 | Covered | No documented cardiovascular or neurologic symptom suggestive of aconite-related toxicity |
| MYT-031 to MYT-062 | POD0 evening to POD2 morning according to patient records | Batch 221026-1 | Luzhou Baicaotang Chinese Herbal Decoction Pieces Co., Ltd. | C-22-102624 | 10 | Covered | No documented cardiovascular or neurologic symptom suggestive of aconite-related toxicity |

*All 62 UBE+MYT patients were matched to documented Aconiti Radix Cocta batches. The detailed de-identified patient-level source records were retained by the study team.*

**Supplementary Table S1B. COA / inspection-report coverage index for clinically used MYT batches.**

| Component | Batch No. | Manufacturer | COA / inspection report No. | Evidence page | Validity | Inspection conclusion | Actual clinical batch | Patient coverage | Comments |
| --- | --- | --- | --- | --- | --- | --- | --- | --- | --- |
| Dipsaci Radix | 220112-1 | Luzhou Baicaotang Chinese Herbal Decoction Pieces Co., Ltd. | C-22-011216 | 20 | Valid through 2027 | Compliant | Yes | MYT-001–MYT-062 | Covered by available COA/inspection report. |
| Poria | 220117-1 | Luzhou Baicaotang Chinese Herbal Decoction Pieces Co., Ltd. | C-22-011713 | 19 | Valid through 2027 | Compliant | Yes | MYT-001–MYT-062 | Covered by available COA/inspection report. |
| Zingiberis Rhizoma | 220310-1 | Luzhou Baicaotang Chinese Herbal Decoction Pieces Co., Ltd. | C-22-031014 | 18 | Valid through 2027 | Compliant | Yes | MYT-001–MYT-062 | Covered by available COA/inspection report. |
| Atractylodis Macrocephalae Rhizoma | 220124-2 | Luzhou Baicaotang Chinese Herbal Decoction Pieces Co., Ltd. | C-22-012413 | 16 | Valid through 2027 | Compliant | Yes | MYT-001–MYT-062 | Covered by available COA/inspection report. |
| Glycyrrhizae Radix et Rhizoma Praeparata cum Melle | 220112-1 | Luzhou Baicaotang Chinese Herbal Decoction Pieces Co., Ltd. | C-22-011216 | 14 | Valid through 2027 | Compliant | Yes | MYT-001–MYT-062 | Covered by available COA/inspection report. |
| Citri Reticulatae Pericarpium | 220119-1 | Luzhou Baicaotang Chinese Herbal Decoction Pieces Co., Ltd. | C-22-011910 | 17 | Valid through 2027 | Compliant | Yes | MYT-001–MYT-062 | Covered by available COA/inspection report. |
| Aconiti Radix Cocta | 211101 | Sichuan Shengshang Da Health Pharmaceutical Co., Ltd. | JBCA06708211101 | 12 | See COA report | Compliant | Yes | MYT-001-MYT-030 | Covered by patient-level matching table. |
| Aconiti Radix Cocta | 221026-1 | Luzhou Baicaotang Chinese Herbal Decoction Pieces Co., Ltd. | C-22-102624 | 10 | See COA report | Compliant | Yes | MYT-031-MYT-062 | Covered by patient-level matching table. |

**Supplementary Table S1C. Aconiti Radix Cocta quality-control and safety-specific evidence closure.**

| Verification item | Evidence source | Status | Result / evidence location | Editorial interpretation |
| --- | --- | --- | --- | --- |
| Identity confirmation | COA reports; pharmacopoeial identity table | Completed | Both clinically used batches were documented as Aconiti Radix Cocta / processed Zhi Chuan Wu, not raw Chuan Wu. | Supports botanical/pharmacopoeial identity and safety-specific reporting. |
| Patient-level batch coverage | Supplementary Table S1A | Completed | 62/62 UBE+MYT patients matched to Aconiti Radix Cocta batches. | Supports statement of complete Aconiti patient-level traceability. |
| COA / inspection-report coverage | Supplementary Table S1B; Data Sheet 2 | Completed | Batch 211101: report JBCA06708211101, p.12; batch 221026-1: report C-22-102624, p.10. | Supports ConPhyMP manufacturer/COA requirement for safety-critical component. |
| Toxic-decoction-piece qualification | Manufacturer license/qualification documents; Data Sheet 3 | Completed | Manufacturer qualification documents collated; author should verify license number, validity, and production scope against original scans. | Supports regulatory-status reporting; final legal details require author confirmation. |
| Aconitine-type alkaloid / safety-related testing | COA reports; Data Sheet 2; Supplementary Table S11 | Completed | Available COA reports include diester-type alkaloid testing and content determination; both clinically used batches met reported specifications. | Supports safety-related QC marker reporting; does not prove clinical safety. |
| Pre-decoction procedure | Institutional decoction SOP; Supplementary Table S2 | Completed | Processed Zhi Chuan Wu was separately pre-decocted according to the institutional decoction procedure before combination with other herbs. | Supports reproducibility and safety transparency. |
| Aconiti-related adverse-event review | Safety verification tables; Supplementary Table S6 | Completed | No clear cardiovascular or neurologic toxicity signal suggestive of aconite-related toxicity was documented in the UBE+MYT cohort. | Supports “no severe safety signal observed,” not proof of absolute safety. |
| Overall conclusion | S1A + S1B + S1C + COA reports | Completed | Patient-level Aconiti Radix Cocta traceability and inspection-report coverage were verified for all 62 UBE+MYT patients. | Use cautiously in manuscript; do not claim complete prospective pharmaceutical standardization. |

*This table addresses the safety-critical aconite-containing component. COA compliance does not establish clinical safety; clinical safety was evaluated separately through adverse-event review.*

**Supplementary Table S2. Institutional decoction and traceability procedure for MYT.**

| Procedure domain | Institutional procedure / verification item | Traceability source | Relevance to reviewer concern |
| --- | --- | --- | --- |
| Prescription review | Pharmacists verified patient information, herbal names, daily doses, processing status, batch information, expiry dates, and available quality-control documents before decoction. | Hospital pharmacy prescription review and dispensing records | Supports reproducibility and prescription-level traceability. |
| Aconiti Radix Cocta handling | Processed Zhi Chuan Wu was verified as Aconiti Radix Cocta and was not substituted by raw Chuan Wu; it was weighed and labelled separately. | Hospital decoction-room SOP and pharmacy records | Addresses safety-critical processing of aconite-containing component. |
| Pre-decoction | Aconiti Radix Cocta 9 g was pre-decocted separately for approximately 60 min before combination with the remaining herbs. | Hospital decoction-room SOP | Supports mitigation of aconite-related safety risk. |
| Combined decoction | The remaining six herbal components were soaked before decoction and then combined with the pre-decocted Aconiti Radix Cocta for routine two-step decoction. | Hospital decoction-room SOP | Supports repeatability of extraction/decoction procedure. |
| Concentration and packaging | Combined filtrates were concentrated to approximately 400 mL per daily dose and divided into two sealed portions of approximately 200 mL each. | Hospital decoction-room packaging records | Supports administration consistency. |
| Storage and dispensing | Sealed decoctions not taken immediately were stored according to pharmacy requirements; dispensing and administration records were retained by the hospital. | Pharmacy/decoction-room and nursing administration records | Supports clinical exposure verification. |
| Administration schedule | MYT was administered orally in two divided warm doses in the morning and evening for a planned 7-day postoperative course. | Electronic medical orders, nursing records, follow-up records | Clarifies treatment timing and duration. |

**Supplementary Table S3. MYT initiation timing and treatment-course adherence in the UBE+MYT cohort.**

| Item | Result | Comment |
| --- | --- | --- |
| Number of UBE+MYT patients | 62/62 | All included patients with documented MYT exposure |
| Planned regimen | One daily decoction dose for 7 postoperative days, divided into morning and evening administrations | Planned total: 14 administrations |
| First administration: POD0 evening | 38/62 | Verified from medical, pharmacy/decoction-room, nursing, and follow-up records |
| First administration: POD1 morning | 21/62 | Verified |
| First administration: POD1 evening | 2/62 | Verified |
| First administration: POD2 morning | 1/62 | Verified |
| Completed 14/14 administrations | 52/62 | Full planned course |
| Completed 13/14 administrations | 4/62 | Minor omission documented |
| Completed 12/14 administrations | 3/62 | Minor omission documented |
| Completed 10-11/14 administrations | 3/62 | Shortened course documented |
| Aconiti Radix Cocta-specific symptoms | 0/62 | No palpitations, arrhythmia, chest tightness, hypotension, perioral numbness, limb numbness, or tremor documented |

**Supplementary Table S4. Standard perioperative pathway and co-interventions in the two UBE cohorts.**

| Domain | UBE+MYT cohort | UBE-alone cohort | Consistency |
| --- | --- | --- | --- |
| Surgical team | Same senior spinal surgery team | Same senior spinal surgery team | Yes |
| Anesthesia | General anesthesia | General anesthesia | Yes |
| Surgical procedure | UBE decompression | UBE decompression | Yes |
| Preoperative fasting | Institutional anesthesia protocol | Institutional anesthesia protocol | Yes |
| Routine analgesia | Institutional analgesic pathway | Institutional analgesic pathway | Yes |
| Rescue analgesia | Triggered by VAS and clinical need | Triggered by VAS and clinical need | Yes |
| Antibiotic prophylaxis | Institutional prophylactic antibiotic protocol | Institutional prophylactic antibiotic protocol | Yes |
| Systemic corticosteroids | Not routinely used postoperatively | Not routinely used postoperatively | Yes |
| Anticoagulant/hemostatic drugs | Not routinely used; risk-based if clinically indicated | Not routinely used; risk-based if clinically indicated | Yes |
| Rehabilitation | Standard early mobilization and brace-protected activity | Standard early mobilization and brace-protected activity | Yes |
| Laboratory monitoring | Preoperative, POD1, POD3, POD7 | Preoperative, POD1, POD3, POD7 | Yes |
| Discharge criteria | Unified institutional criteria | Unified institutional criteria | Yes |
| MYT exposure | MYT administered as adjunctive treatment | No MYT and no placebo | No; study exposure |
| Routine early analgesia | Parecoxib sodium; 40 mg intravenously every 12 h; POD0-POD2 or until oral transition | Parecoxib sodium; 40 mg intravenously every 12 h; POD0-POD2 or until oral transition | Yes |
| Oral transition analgesia | Celecoxib; 200 mg orally twice daily; From POD2 when tolerated, generally to POD5-POD7 | Celecoxib; 200 mg orally twice daily; From POD2 when tolerated, generally to POD5-POD7 | Yes |
| Gastroprotection | Proton-pump inhibitor; Institutional standard dose; During NSAID/COX-2 use as clinically indicated | Proton-pump inhibitor; Institutional standard dose; During NSAID/COX-2 use as clinically indicated | Yes |
| Rescue analgesia | Tramadol; 50 mg orally or intramuscularly every 8 h as needed; VAS >=4 or breakthrough pain | Tramadol; 50 mg orally or intramuscularly every 8 h as needed; VAS >=4 or breakthrough pain | Yes |
| Strong opioids | Not routinely used; None in the UBE analytic cohorts; Not applicable | Not routinely used; None in the UBE analytic cohorts; Not applicable | Yes |
| Antibiotic prophylaxis | Cefuroxime sodium; 1.5 g IV 30-60 min before incision; then every 12 h, total duration <=24 h; Used in both groups according to the same protocol | Cefuroxime sodium; 1.5 g IV 30-60 min before incision; then every 12 h, total duration <=24 h; Used in both groups according to the same protocol | Yes |
| Alternative for cephalosporin allergy | Clindamycin; 600 mg IV according to the institutional antimicrobial protocol; Used only when clinically indicated | Clindamycin; 600 mg IV according to the institutional antimicrobial protocol; Used only when clinically indicated | Yes |
| Systemic corticosteroids | Methylprednisolone/dexamethasone; Not routinely used as postoperative anti-inflammatory treatment; No routine postoperative systemic corticosteroid regimen | Methylprednisolone/dexamethasone; Not routinely used as postoperative anti-inflammatory treatment; No routine postoperative systemic corticosteroid regimen | Yes |
| Anticoagulants | Low-molecular-weight heparin; Not routinely used after UBE; used only after individual VTE-risk evaluation; No routine use in the analytic UBE cohorts | Low-molecular-weight heparin; Not routinely used after UBE; used only after individual VTE-risk evaluation; No routine use in the analytic UBE cohorts | Yes |
| Hemostatic agents | Tranexamic acid/hemostatic agents; Not routinely used after UBE; No routine use in the analytic UBE cohorts | Tranexamic acid/hemostatic agents; Not routinely used after UBE; No routine use in the analytic UBE cohorts | Yes |
| Mechanical VTE prevention | Ankle-pump exercise and early mobilization; Started after recovery from anesthesia; Used in both groups | Ankle-pump exercise and early mobilization; Started after recovery from anesthesia; Used in both groups | Yes |
| Other herbal formulas or Chinese patent medicines | None routinely co-administered; Not routinely combined during the perioperative observation window; None documented as routine co-intervention | None routinely co-administered; Not routinely combined during the perioperative observation window; None documented as routine co-intervention | Yes |

*Routine analgesic and concomitant-intervention criteria were the same across UBE cohorts; observed rescue analgesia use is summarized below.*

| Indicator | UBE+MYT (n=62) | UBE alone (n=60) |
| --- | --- | --- |
| Routine COX-2-based analgesia | 62/62 (100.0%) | 60/60 (100.0%) |
| Rescue analgesia use | 8/62 (12.9%) | 17/60 (28.3%) |
| Strong opioid use | 0/62 (0.0%) | 0/60 (0.0%) |
| Analgesic discontinuation due to intolerance | 0/62 (0.0%) | 1/60 (1.7%) |

**Supplementary Table S5. Laboratory sampling schedule and completeness.**

| Time point | Sampling window | Tests | Notes |
| --- | --- | --- | --- |
| Preoperative baseline | Within 24 h before surgery, usually 06:00-08:00 | IL-6, CRP, D-dimer, liver/renal function and routine perioperative labs | Baseline before surgical exposure |
| POD1 | 06:00-08:00 on postoperative day 1 | IL-6, CRP, D-dimer | Morning sampling before oral MYT/analgesic administration when applicable |
| POD3 | 06:00-08:00 on postoperative day 3 | IL-6, CRP, D-dimer | Same time window |
| POD7 | 06:00-08:00 on postoperative day 7 | IL-6, CRP, D-dimer | Same time window; inpatient or scheduled review sampling |
| Indicator | UBE+MYT (n=62) | UBE alone (n=60) | Consistency |
| Preoperative baseline sampling complete | 62/62 | 60/60 | Yes |
| POD1 sampling complete | 62/62 | 60/60 | Yes |
| POD3 sampling complete | 62/62 | 60/60 | Yes |
| POD7 sampling complete | 62/62 | 60/60 | Yes |
| Morning sampling window | 06:00-08:00 | 06:00-08:00 | Yes |

**Supplementary Table S6. Adverse-event adjudication and safety summary.**

| Study ID | Group | Event | Onset | | Grade | Management | Outcome | | Readmission / reoperation | Relationship to MYT | Zhi Chuan Wu symptoms |  |
| --- | --- | --- | --- | --- | --- | --- | --- | --- | --- | --- | --- | --- |
| MYT-AE01 | UBE+MYT | Transient nausea/vomiting | POD1 | | Grade I | Temporary oral intake delay, symptomatic antiemetic treatment and observation | Resolved on POD2 | | No/No | Uncertain; anesthesia or analgesic medication more likely | No palpitations, arrhythmia, chest tightness, hypotension, perioral numbness, limb numbness, or tremor |  |
| MYT-AE02 | UBE+MYT | Mild diarrhea | POD3 | | Grade I | Dietary adjustment, oral hydration/observation; no antibiotic escalation | Resolved on POD4 | | No/No | Possible | No palpitations, arrhythmia, chest tightness, hypotension, perioral numbness, limb numbness, or tremor |  |
| UBE-AE01 | UBE alone | Postoperative nausea/vomiting | POD1 | | Grade I | Symptomatic antiemetic treatment and observation | Resolved on POD2 | | No/No | Not applicable | Not applicable |  |
| UBE-AE02 | UBE alone | Dizziness/orthostatic discomfort | POD1 | | Grade I | Rest, hydration, and observation | Resolved on POD2 | | No/No | Not applicable | Not applicable |  |
| UBE-AE03 | UBE alone | Mild wound exudation | POD2 | | Grade I | Enhanced wound dressing; no antibiotic escalation | Improved by POD4 | | No/No | Not applicable | Not applicable |  |
| UBE-AE04 | UBE alone | Constipation | POD3 | | Grade I | Dietary guidance and laxative management | Resolved by POD5 | | No/No | Not applicable | Not applicable |  |
| UBE-AE05 | UBE alone | Transient fever | POD2 | | Grade I | Physical cooling and observation; no infection focus identified | Resolved on POD3 | | No/No | Not applicable | Not applicable |  |
| UBE-AE06 | UBE alone | Transient urinary retention | POD1 | | Grade I | Intermittent catheterization once and observation | Spontaneous urination recovered on POD2 | | No/No | Not applicable | Not applicable |  |
| UBE-AE07 | UBE alone | Urinary tract infection | POD4 | | Grade II | Oral antibiotic therapy | Improved by POD7 | | No/No | Not applicable | Not applicable |  |
| UBE-AE08 | UBE alone | Superficial wound infection | POD5 | | Grade II | Oral antibiotic therapy and enhanced wound care | Healed within 2 weeks | | No/No | Not applicable | Not applicable |  |
| UBE-AE09 | UBE alone | Moderate gastrointestinal reaction | POD2 | | Grade II | NSAID adjustment, gastroprotection and antiemetic medication | Resolved by POD4 | | No/No | Not applicable | Not applicable |  |
| Safety indicator | | | | UBE+MYT (n=62) | | | | UBE alone (n=60) | | | | |
| Any postoperative adverse event | | | | 2/62 (3.2%) | | | | 9/60 (15.0%) | | | | |
| Clavien-Dindo Grade I | | | | 2/62 (3.2%) | | | | 6/60 (10.0%) | | | | |
| Clavien-Dindo Grade II | | | | 0/62 (0.0%) | | | | 3/60 (5.0%) | | | | |
| Clavien-Dindo Grade >=III | | | | 0/62 (0.0%) | | | | 0/60 (0.0%) | | | | |
| Readmission | | | | 0/62 (0.0%) | | | | 0/60 (0.0%) | | | | |
| Reoperation | | | | 0/62 (0.0%) | | | | 0/60 (0.0%) | | | | |
| Severe hepatic or renal dysfunction | | | | 0/62 (0.0%) | | | | 0/60 (0.0%) | | | | |
| Allergic reaction | | | | 0/62 (0.0%) | | | | 0/60 (0.0%) | | | | |
| Cardiovascular/neurologic symptoms suggestive of Zhi Chuan Wu toxicity | | | | 0/62 (0.0%) | | | | Not applicable | | | | |

**Supplementary Table S7. IPTW diagnostics, covariate balance, and effective sample size.**

| Covariate | Type | | SMD before | SMD after IPTW | | SMD after overlap weighting |
| --- | --- | --- | --- | --- | --- | --- |
| Age | Continuous | | 0.061 | 0.002 | | 0.000 |
| Male sex | Binary | | 0.133 | 0.014 | | 0.000 |
| Preoperative VAS | Continuous | | 0.128 | 0.003 | | 0.000 |
| Preoperative JOA | Continuous | | 0.112 | 0.011 | | 0.000 |
| Preoperative TCM syndrome score | Continuous | | 0.089 | 0.005 | | 0.000 |
| Preoperative CRP (mg/L) | Continuous | | 0.113 | 0.007 | | 0.000 |
| Preoperative D-dimer (ug/mL) | Continuous | | 0.015 | 0.011 | | 0.000 |
| Preoperative IL-6 (pg/mL) | Continuous | | 0.005 | 0.004 | | 0.000 |
| Preoperative disc height (mm) | Continuous | | 0.315 | 0.008 | | 0.000 |
| Preoperative spinal canal volume | Continuous | | 0.158 | 0.014 | | 0.000 |
| Herniation level: Upper lumbar | Binary | | 0.068 | 0.006 | | 0.000 |
| Herniation level: L4/5 | Binary | | 0.141 | 0.014 | | 0.000 |
| Herniation level: L5/S1 | Binary | | 0.191 | 0.012 | | 0.000 |
| Diagnostic item | | Value | | | Interpretation | |
| n in UBE analytic cohort | | 122 | | | 62 UBE+MYT and 60 UBE-alone patients | |
| Maximum SMD before IPTW | | 0.315 | | | Largest baseline imbalance before weighting | |
| Maximum SMD after IPTW | | 0.014 | | | All covariates below 0.10 after weighting | |
| Maximum stabilized weight | | 2.064 | | | No problematic extreme weights observed | |
| Weights > 10 | | 0 | | | No extreme weights above 10 | |
| Effective sample size overall | | 113.902 | | | Preserved most of the original sample size | |
| ESS UBE+MYT | | 57.981 | | | Treatment group ESS | |
| ESS UBE alone | | 55.921 | | | Control group ESS | |
| Matched pairs in PSM sensitivity analysis | | 43 | | | Directionally consistent with primary IPTW | |

**Supplementary Table S8. Sensitivity analyses for main early recovery outcomes.**

| Outcome | Method | Estimate | 95% CI | P value | Direction consistent | Matched pairs |
| --- | --- | --- | --- | --- | --- | --- |
| VAS POD3 | Main stabilized IPTW | -0.792 | -1.061 to -0.522 | 8.42e-09 | Yes | NA |
| VAS POD3 | Trimmed IPTW (1st/99th percentile) | -0.794 | -1.063 to -0.525 | 7.08e-09 | Yes | NA |
| VAS POD3 | Overlap weighting | -0.813 | -1.081 to -0.546 | 2.49e-09 | Yes | NA |
| VAS POD3 | 1:1 propensity-score matching | -0.930 | -1.248 to -0.612 | 5.41e-07 | Yes | 43 |
| VAS POD3 | Multivariable regression adjustment | -0.815 | -1.106 to -0.525 | 3.85e-08 | Yes | NA |
| IL-6 POD3 | Main stabilized IPTW | -11.515 | -13.121 to -9.909 | 7.16e-45 | Yes | NA |
| IL-6 POD3 | Trimmed IPTW (1st/99th percentile) | -11.533 | -13.137 to -9.929 | 3.99e-45 | Yes | NA |
| IL-6 POD3 | Overlap weighting | -11.557 | -13.176 to -9.939 | 1.68e-44 | Yes | NA |
| IL-6 POD3 | 1:1 propensity-score matching | -11.309 | -13.283 to -9.336 | 1.22e-14 | Yes | 43 |
| IL-6 POD3 | Multivariable regression adjustment | -11.552 | -13.270 to -9.834 | 1.13e-39 | Yes | NA |
| VAS POD7 | Main stabilized IPTW | -0.882 | -1.147 to -0.617 | 6.4e-11 | Yes | NA |
| VAS POD7 | Trimmed IPTW (1st/99th percentile) | -0.881 | -1.146 to -0.616 | 6.89e-11 | Yes | NA |
| VAS POD7 | Overlap weighting | -0.891 | -1.151 to -0.631 | 1.85e-11 | Yes | NA |
| VAS POD7 | 1:1 propensity-score matching | -0.837 | -1.105 to -0.569 | 1.45e-07 | Yes | 43 |
| VAS POD7 | Multivariable regression adjustment | -0.892 | -1.169 to -0.616 | 2.38e-10 | Yes | NA |
| IL-6 POD7 | Main stabilized IPTW | -5.621 | -6.413 to -4.828 | 6.77e-44 | Yes | NA |
| IL-6 POD7 | Trimmed IPTW (1st/99th percentile) | -5.623 | -6.418 to -4.828 | 1e-43 | Yes | NA |
| IL-6 POD7 | Overlap weighting | -5.605 | -6.401 to -4.808 | 2.87e-43 | Yes | NA |
| IL-6 POD7 | 1:1 propensity-score matching | -5.442 | -6.352 to -4.531 | 3.13e-15 | Yes | 43 |
| IL-6 POD7 | Multivariable regression adjustment | -5.599 | -6.458 to -4.740 | 2.35e-37 | Yes | NA |
| CRP POD3 | Main stabilized IPTW | -5.331 | -7.238 to -3.424 | 4.28e-08 | Yes | NA |
| CRP POD3 | Trimmed IPTW (1st/99th percentile) | -5.327 | -7.236 to -3.418 | 4.51e-08 | Yes | NA |
| CRP POD3 | Overlap weighting | -5.395 | -7.316 to -3.474 | 3.71e-08 | Yes | NA |
| CRP POD3 | 1:1 propensity-score matching | -5.539 | -7.906 to -3.171 | 2.62e-05 | Yes | 43 |
| CRP POD3 | Multivariable regression adjustment | -5.398 | -7.375 to -3.421 | 8.74e-08 | Yes | NA |
| D-dimer POD3 | Main stabilized IPTW | -0.887 | -1.109 to -0.665 | 5.29e-15 | Yes | NA |
| D-dimer POD3 | Trimmed IPTW (1st/99th percentile) | -0.887 | -1.109 to -0.664 | 6.18e-15 | Yes | NA |
| D-dimer POD3 | Overlap weighting | -0.896 | -1.126 to -0.667 | 1.79e-14 | Yes | NA |
| D-dimer POD3 | 1:1 propensity-score matching | -0.904 | -1.203 to -0.606 | 2.73e-07 | Yes | 43 |
| D-dimer POD3 | Multivariable regression adjustment | -0.897 | -1.140 to -0.653 | 5.62e-13 | Yes | NA |
| JOA 1 month | Main stabilized IPTW | 1.598 | 1.248 to 1.947 | 3.52e-19 | Yes | NA |
| JOA 1 month | Trimmed IPTW (1st/99th percentile) | 1.601 | 1.251 to 1.950 | 2.88e-19 | Yes | NA |
| JOA 1 month | Overlap weighting | 1.616 | 1.270 to 1.961 | 5.38e-20 | Yes | NA |
| JOA 1 month | 1:1 propensity-score matching | 1.651 | 1.278 to 2.024 | 2.96e-11 | Yes | 43 |
| JOA 1 month | Multivariable regression adjustment | 1.613 | 1.233 to 1.994 | 9.9e-17 | Yes | NA |

**Supplementary Table S9. Concise summary of annotated constituents in the MYT sample.**

*A total of 187 constituents were annotated in the MYT sample by UHPLC-Q-Orbitrap HRMS. To maintain readability, this table provides a concise class-level summary and representative annotated constituents; the complete original annotation output is provided in Supplementary Data Sheet 5.*

| Chemical class | | | Number of annotated constituents | | |
| --- | --- | --- | --- | --- | --- |
| Prenol lipids | | | 60 | | |
| Flavonoids | | | 52 | | |
| Organooxygen compounds | | | 15 | | |
| Isoflavonoids | | | 11 | | |
| Phenols | | | 8 | | |
| Other annotated classes | | | 41 | | |
| Representative constituent | Formula | Chemical class | Assigned source component(s) | Reference-substance assisted | Retention time (min) |
| Loganic acid | C16H24O10 | Prenol lipids | Dipsaci Radix | Yes | 6.86 |
| Hesperidin | C28H34O15 | Flavonoids | Citri Reticulatae Pericarpium,Dipsaci Radix,Atractylodis Macrocephalae Rhizoma | Yes | 12.98 |
| Glycyrrhizic acid | C42H62O16 | Prenol lipids | Glycyrrhizae Radix et Rhizoma Praeparata cum Melle | Yes | 18.99 |
| Neoliquiritin | C21H22O9 | Flavonoids | Citri Reticulatae Pericarpium | Unassigned | 10.90 |
| 6-Gingerol | C17H26O4 | Phenols | Zingiberis Rhizoma | Yes | 19.51 |
| Magnoflorine | C20H24NO4+ | Aporphines | Aconiti Radix Cocta,Dipsaci Radix,Atractylodis Macrocephalae Rhizoma,Citri Reticulatae Pericarpium | Yes | 8.80 |
| Aconitine | C34H47NO11 | Prenol lipids | Aconiti Radix Cocta | Yes | 17.19 |
| Hypaconitine | C33H45NO10 | Prenol lipids | Aconiti Radix Cocta | Yes | 17.19 |
| Benzoylmesaconine | C31H43NO10 | Prenol lipids | Aconiti Radix Cocta | Yes | 13.43 |
| Asperosaponin VI | C47H76O18 | Prenol lipids | Dipsaci Radix,Citri Reticulatae Pericarpium | Yes | 17.31 |
| Nobiletin | C21H22O8 | Flavonoids | Citri Reticulatae Pericarpium,Dipsaci Radix | Yes | 19.56 |
| Higenamine | C16H17NO3 | Isoquinolines and derivatives | Aconiti Radix Cocta | Yes | 6.07 |

**Supplementary Table S10. Concise summary of putative MYT-related constituents and metabolites detected in medicated serum.**

*A total of 75 putative MYT-related serum features were detected, including 22 prototype constituents and 53 metabolites. Full annotation details are provided in Supplementary Data Sheet 5.*

| Type | | | Number of detected features | | |
| --- | --- | --- | --- | --- | --- |
| Prototype constituents | | | 22 | | |
| Metabolites | | | 53 | | |
| Assigned source component | | | Number of serum features | | |
| Citri Reticulatae Pericarpium | | | 23 | | |
| Glycyrrhizae Radix et Rhizoma Praeparata cum Melle | | | 19 | | |
| Aconiti Radix Cocta | | | 15 | | |
| Dipsaci Radix | | | 14 | | |
| Zingiberis Rhizoma | | | 11 | | |
| Atractylodis Macrocephalae Rhizoma | | | 10 | | |
| Poria | | | 4 | | |
| Formula-related or non-herb-specific feature | | | 1 | | |
| Representative prototype constituent | Formula | Detection mode | | Retention time (min) | Assigned source component(s) |
| Higenamine | C16H17NO3 | POS | | 6.04 | Aconiti Radix Cocta |
| Loganic acid | C16H24O10 | POS | | 7.17 | Dipsaci Radix |
| tecomoside | C16H24O10 | NEG | | 7.26 | Dipsaci Radix |
| Magnoflorine | C20H24NO4+ | POS | | 8.77 | Aconiti Radix Cocta,Dipsaci Radix,Atractylodis Macrocephalae Rhizoma,Citri Reticulatae Pericarpium |
| Sweroside | C16H22O9 | POS | | 8.98 | Dipsaci Radix |
| Loganin | C17H26O10 | POS | | 9.05 | Dipsaci Radix |
| 4-O-Feruloylquinic acid | C17H20O9 | POS | | 9.62 | Atractylodis Macrocephalae Rhizoma,Citri Reticulatae Pericarpium |
| Talatisamine | C24H39NO5 | POS | | 9.67 | Aconiti Radix Cocta |
| Vitexin | C21H20O10 | POS | | 10.82 | Citri Reticulatae Pericarpium |
| picrocrocinic acid | C16H26O8 | NEG | | 11.15 | Citri Reticulatae Pericarpium |
| Narcissoside | C28H32O16 | POS | | 12.37 | Citri Reticulatae Pericarpium |
| 6,7,4'-Trihydroxyflavanone | C15H12O5 | POS | | 12.57 | Glycyrrhizae Radix et Rhizoma Praeparata cum Melle |

**Supplementary Table S11. Marker and safety-related quality-control quantification for Aconiti Radix Cocta.**

| Component | Batch No. | Manufacturer | Marker / QC item | Method/source | Reported result | Specification / limit | Conclusion | Evidence page | Interpretation |
| --- | --- | --- | --- | --- | --- | --- | --- | --- | --- |
| Aconiti Radix Cocta | 221026-1 | Luzhou Baicaotang Chinese Herbal Decoction Pieces Co., Ltd. | Diester-type alkaloids | Batch COA / Chinese Pharmacopoeia method | 0.002% | NMT 0.040% | Compliant | 10 | Safety-related QC marker; author should verify numeric value against original COA. |
| Aconiti Radix Cocta | 221026-1 | Luzhou Baicaotang Chinese Herbal Decoction Pieces Co., Ltd. | Content determination item | Batch COA / Chinese Pharmacopoeia method | 0.082% | 0.070%-0.150% | Compliant | 10 | Quality-control marker; author should verify numeric value against original COA. |
| Aconiti Radix Cocta | 211101 | Sichuan Shengshang Da Health Pharmaceutical Co., Ltd. | Diester-type alkaloids | Batch COA / Chinese Pharmacopoeia method | 0.007% | NMT 0.040% | Compliant | 12 | Safety-related QC marker; author should verify numeric value against original COA. |
| Aconiti Radix Cocta | 211101 | Sichuan Shengshang Da Health Pharmaceutical Co., Ltd. | Content determination item | Batch COA / Chinese Pharmacopoeia method | 0.13% | 0.070%-0.150% | Compliant | 12 | Quality-control marker; author should verify numeric value against original COA. |

*NMT, not more than. These values are safety-related and quality-control markers from batch COA reports; authors should verify numeric values against original COA pages before submission.*
