# Supplementary material for: Association of postoperative modified Yaotong Tang with early recovery after unilateral biportal endoscopy for lumbar disc herniation: a retrospective comparative cohort study using propensity score weighting
Source: Front Pharmacol. 2026 Jul 9;17:1852732. doi: 10.3389/fphar.2026.1852732 (PMC13391915; doi:10.3389/fphar.2026.1852732)
Supplement: Supplementary file 4 [file DataSheet3.pdf]

# Supplementary Data Sheet 3. Manufacturer Licenses and Qualification Documents

Purpose: This file provides English-indexed manufacturer license and qualification documents supporting the regulatory status and traceability of the herbal decoction pieces used in Modified Yaotong Tang (MYT). Original documents are in Chinese; key fields are summarized in English below. Small text in scanned certificates should be finally verified by the authors against the original documents before submission.

| Evidence pages in this data sheet | Manufacturer | Document type | Key information indexed | Related MYT component / batch | Author confirmation |
|---|---|---|---|---|---|
| 2-3 | Luzhou Baicaotang Chinese Herbal Decoction Pieces Co., Ltd. | Business license | Business registration and scope (Chinese scan). | Non-aconite MYT components and Aconiti Radix Cocta batch [illegible] where applicable. | Verify Chinese legal name, registration number and business sco[illegible] |
| 4-7 | Luzhou Baicaotang Chinese Herbal Decoction Pieces Co., Ltd. | Drug manufacturing license and pages showing [illegible] | Licensing information and [illegible] scope for Chinese herbal decoction pieces [illegible] | MYT batches supplied by Luzhou Baicaotang, including [illegible] | Verify license No., validity period and [illegible] |
| 8-9 | Luzhou Baicaotang Chinese Herbal Decoction Pieces Co., Ltd. | Supplier qualification / archive records | Supplier qualification information retained for pharmacy procurement/traceability. | Luzhou Baicaotang-supplied MYT components. | Verify against hospital procurement archive. |
| 10-11 | Luzhou Baicaotang Chinese Herbal Decoction Pieces Co., Ltd. | Public/archival qualification pages | Additional qualification/context pages included where relevant. | Luzhou Baicaotang-supplied MYT components. | Verify whether these pages should be uploaded publicly or retain[illegible] |
| 12 | Sichuan Shengshang Da Health Pharmaceutical Co., Ltd. | Drug manufacturing license | License scan for manufacturer of Aconiti Radix Cocta batch 211101. | Aconiti Radix Cocta batch 211101. | Verify license No., validity period and production scope. |
| 13 | Sichuan Shengshang Da Health Pharmaceutical Co., Ltd. | Business license | Business license scan for manufacturer of Aconiti Radix Cocta batch 211101. | Aconiti Radix Cocta batch 211101. | Verify legal name and registration details. |

Note: This English index does not replace the original Chinese certificates. It is intended to help editors and reviewers locate the relevant manufacturer/license evidence. Documents unrelated to manufacturer licensing or qualification, such as bank-account certificates, stamp specimen sheets, and invoice templates, are not included in this final upload version unless explicitly required by the editor.

# 营业执照

统一社会信用代码
91510502771664 93XY

扫描经营主体信息码，了解更多登记、备案、许可、监管信息。

名　　称　泸州百草堂中药饮片有限公司

类　　型　其他有限责任公司

法定代表人　高代军

经营范围　生产、销售：中药饮片(净制、切制、炒制、烫制、煅制、制炭、蒸制、煮制、炖制、燀制、酒制、醋制、盐制、姜汁炙、蜜炙、油炙、制霜、水飞、煨制）、毒性饮片（净制、切制、炒制、炙制、蒸制、煮制）、直接口服饮片（以上经营项目凭许可证经营）。销售：化妆品、机械设备、化工产品（不含危险化学品）、植物药提取、中药材种植、销售；技术培训及信息咨询（不得从事非法集资、吸收公众资金等金融活动）。（依法须经批准的项目，经相关部门批准后方可开展经营活动）

注册资本　壹仟肆佰柒拾壹万壹仟零玖拾叁元整

成立日期　2005年01月24日

住　　所　泸州市江阳区泸州轻工业园区拥军路一段52号1-2层

仅供存档 复印无效

登记机关　泸州市江阳区市场监督管理局

2025 年 9 月 28 日

# 营 业 执 照

（副 本）

统一社会信用代码

91510502771664 93XY

扫描经营主体信息码、了解更多登记、备案、许可、监管信息。

| | |
|---|---|
| 名　　称 | 泸州百草堂中药饮片有限公司 |
| 类　　型 | 其他有限责任公司 |
| 法定代表人 | 高代军 |
| 经营范围 | 生产、销售：中药饮片(净制、切制、炒制、烫制、煅制、制炭、蒸制、煮制、炖制、燀制、酒制、醋制、盐制、姜汁炙、蜜炙、油炙、制霜、水飞、煨制）、毒性饮片（净制、切制、炒制、炙制、蒸制、煮制）、直接口服饮片（以上经营项目凭许可证经营）；销售：化妆品、机械设备、化工产品（不含危险化学品）；植物药提取、中药材种植、销售；技术培训及信息咨询（不得从事非法集资、吸收公众资金等金融活动）。（依法须经批准的项目，经相关部门批准后方可开展经营活动） |
| 注册资本 | 壹仟肆佰柒拾壹万壹仟零玖拾叁元整 |
| 成立日期 | 2005年01月24日 |
| 住　　所 | 泸州市江阳区泸州轻工业园区拥军路一段52号1-2层 |

仅供存档
复印无效

5105025048836

登 记 机 关

泸州市江阳区市场监督管理局

2025 年 9 月 28 日

市场主体应当于每年1月1日至6月30日通过国

国家市场监督管理总局监制

# 药品生产许可证

企业名称：泸州百草堂中药饮片有限公司

许可证编号：川 20160183

社会信用代码：9151050277166493XY

分类码：Ay

注册地址：泸州市江阳区泸州轻工业园区拥军路一段 52 号 1-2 层

日常监督管理机构：四川省药品监督管理局第三检查分局

法定代表人：高代军

投诉举报电话：123[illegible]

企业负责人：高代军

质量负责人：梁英

发证机关：四川省药品监督管理局

生产地址和生产范围：

泸州轻工业园区：毒性饮片，中药饮片，直接口服饮片***

签发人：蒲素

2025 年 11 月 20 日

有效期至 2030 年 10 月 13 日

[illegible]

# 药品生产许可证

## （副本）

企业名称：泸州百草堂中药饮片有限公司

许可证编号：川20160183

社会信用代码：91510502771664 93XY

分类码：Ay

注册地址：泸州市江阳区泸州轻工业园区拥军路一段52号1-2层

法定代表人：高代军

企业负责人：高代军

质量负责人：梁英

质量受权人：梁英

生产负责人：陈万铭

有效期至 2030 年 10 月 13 日

**生产地址和生产范围：**

泸州轻工业园区：毒性饮片(净制、切制、炒制、醋炙、煮制)，中药饮片(净制、切制、炒制、煅制、制炭、蒸制、煮制、炖制、燀制、酒炙、醋炙、盐炙、姜炙、蜜炙、油炙、煨制)，直接口服饮片***

发证机关：四川省药品监督管理局

2025年1[illegible]月20 日

## 变更记录

**事项：**

同意该企业注册地址由“泸州轻工业园区”变更为“泸州市江阳区泸州轻工业园区拥军路一段52号1-2层”；法定代表人由“张显”变更为“高代军”；其他内容不变。

四川省药品监督管理局

（盖章）

2025 年 [illegible] 月 20 日

**事项：**

（盖章）

年 月 日

**事项：**

（盖章）

年 月 日

## 变更记录

**事项：**

泸州百草堂中药饮片有限公司

（盖章）

年 月 日

**事项：**

（盖章）

年 月 日

**事项：**

（盖章）

年 月 日

一、车间及生产线情况

| 生产地址 | 车间 | 生产线 | 范围 |
|---|---|---|---|
| 泸州轻工业园区 | 毒性饮片车间 | 毒性饮片生产线 | 毒性饮片(净制、切制、炒制、醋炙、煮制) |
| 泸州轻工业园区 | 中药饮片车间 | 中药饮片生产线 | 中药饮片(净制、切制、炒制、煅制、制炭、蒸制、煮制、炖制、燀制、酒炙、醋炙、盐炙、姜炙、蜜炙、油炙、煨制) |
| 泸州轻工业园区 | 直接口服饮片车间 | 直接口服饮片生产线 | 直接口服饮片 |

二、委托或受托情况

| 类型 | 企业名称 | 生产/注册地址 | 药品通用名称 | 药品批准文号 | 委托有效期 |
|---|---|---|---|---|---|
| | | | | | |

# 企业质量体系调查表

<table>
<tr><td>企业名称</td><td colspan="3">泸州百草堂中药饮片有限公司</td><td colspan="2">电话</td><td colspan="2">0830-3999886</td></tr>
<tr><td>地址</td><td colspan="3">泸州市江阳区泸州轻工业园区拥军路一段 52 号 1-2 层</td><td colspan="2">邮政编码</td><td colspan="2">646000</td></tr>
<tr><td>经营方式</td><td colspan="3">产、供、销</td><td colspan="2">生产许可证号</td><td colspan="2">川 20160183</td></tr>
<tr><td>生产范围</td><td colspan="7">毒性饮片、中药饮片、直接口服饮片</td></tr>
<tr><td rowspan="3">法定代表人</td><td>姓名</td><td colspan="2">高代军</td><td rowspan="3">销售员</td><td>姓名</td><td colspan="2">/</td></tr>
<tr><td>职称</td><td colspan="2">/</td><td>职务</td><td colspan="2">/</td></tr>
<tr><td>电话</td><td colspan="2">0830-3998470</td><td>电话</td><td colspan="2">/</td></tr>
<tr><td>质量负责人</td><td>姓名</td><td colspan="2">梁英</td><td>职称</td><td colspan="3">执业中药师</td></tr>
<tr><td>企业职工人数</td><td>130 人</td><td>质管人员数</td><td>24 人</td><td>企业固定资产</td><td>4965 万元</td><td>全年销售额</td><td>12873 万元</td></tr>
<tr><td>供货品种目录</td><td colspan="7">毒性饮片、中药饮片、直接口服饮片</td></tr>
<tr><td>企业质量管理情况简介</td><td colspan="7">建立质量管理体系，严把产品质量关，确保产品合格，人民用药安全有效。</td></tr>
</table>

# 合格供货方档案表

编号：　　　　　　　　　　　　　　　　　　　　　　建档时间：

| | | | | | | |
|---|---|---|---|---|---|---|
| 企业名称 | 泸州百草堂中药饮片有限公司 | | 地址 | 泸州市江阳区泸州轻工业园区拥军路一段 52 号 1-2 层 | | |
| 法定代表人 | 高代军 | | 联系电话 | 0830-3999886 | 邮编 | 646000 |
| 生产许可证号 | 川 20160183 | | 营业执照号 | 9151050277166493XY | | |
| 生产（经营）范围 | 中药饮片、毒性饮片、直接口服饮片 | | 经营方式 | 生产、销售 | | |
| 企业概况 | 年产值销售额 | 1.2873 亿 | 质量认证情况 | GMP 认证 | | |
| 主要产品 | 中药饮片、直接口服饮片 | | | | | |
| 质量管理机构 | 负责人姓名 | 梁英 | 人数 | 24 | 联系方式 | 18015720823 |
| 综合评价 | 审核以上资料符合规定，可以列入合格供货方档案 | | | | | |
| 备注 | | | | | | |

# 2024年度报告 0条修改记录

填报时间:2025年05月21日

企业年报信息由该企业提供，企业对其年报信息的真实性、合法性负责

## 基本信息

- **统一社会信用代码/注册号：** 9151050277166493XY
- **企业名称：** 泸州百草堂中药饮片有限公司
- **企业通信地址：** 泸州市江阳区拥军路一段52号
- **邮政编码：** 646000
- **企业联系电话：** 0830-3897691
- **企业电子邮箱：** 3597233510@qq.com
- **从业人数：** 企业选择不公示
- **其中女性从业人数：** 企业选择不公示
- **企业经营状态：** 开业
- **企业控股情况：** 企业选择不公示
- **是否有投资信息或购买其他公司股权：** 否
- **是否有网站或网店：** 是
- **是否有对外提供担保信息：** 否
- **有限责任公司本年度是否发生股东股权转让：** 否
- **企业主营业务活动：** 生产、销售：中药饮片（净制、切制、炒制、烫制、煅制、制炭、蒸制、煮制、炖制、燀制、酒制、醋制、盐制、姜汁炙、蜜炙、油炙、制霜、水飞、煨制），毒性饮片（净制、切制、炒制、炙制、蒸制、煮制），直接口服饮片；销售：化妆品、机械设备、化工产品（不含危险化学品）；植物药提取；中药材种植、销售；技术培训及信息咨询。

## 网站或网店信息

共计 1 条信息

泸州百草堂中药饮片有限公司

- 类型：网站
- 网址：http://www.lzbct.cn

## 股东及出资信息

| 序号 | 股东 | 认缴出资额（万元） | 认缴出资时间 | 认缴出资方式 | 实缴出资额（万元） | 实缴出资时间 | 实缴出资方式 |
|---|---|---|---|---|---|---|---|
| 1 | 范华静 | 12.32 | 2012年6月20日 | 货币 | 12.32 | 2012年6月20日 | 货币 |
| 2 | 泸州佳希实业有限公司 | 1168.0695 | 2016年3月21日 | 货币 | 1168.0695 | 2016年3月21日 | 货币 |
| 3 | 黄涛 | 71.3488 | 2012年9月5日 | 货币 | 71.3488 | 2012年9月5日 | 货币 |
| 4 | 上官子恒 | 25.8 | 2010年11月10日 | 货币 | 25.8 | 2010年11月10日 | 货币 |
| 5 | 李彬 | 8.6 | 2010年11月10日 | 货币 | 8.6 | 2010年11月10日 | 货币 |

共查询到 7 条记录 共 2 页

首页 上一页 1 2 下一页 末页

## 对外投资信息

暂无对外投资信息

## 企业资产状况信息

| | | | |
|---|---|---|---|
| 资产总额 | 企业选择不公示 | 所有者权益合计 | 企业选择不公示 |
| 营业总收入 | 企业选择不公示 | 利润总额 | 企业选择不公示 |
| 营业总收入中主营业务收入 | 企业选择不公示 | 净利润 | 企业选择不公示 |

| | | | |
|---|---|---|---|
| 纳税总额 | 企业选择不公示 | 负债总额 | 企业选择不公示 |

对外提供保证担保信息

| 序号 | 债权人 | 债务人 | 主债权种类 | 主债权数额 | 履行债务的期限 | 保证的期间 | 保证的方式 |
|---|---|---|---|---|---|---|---|
| 暂无对外提供保证担保信息 | | | | | | | |

共 查询到 0 条记录 共 0 页 首页 上一页 下一页 末页

股权变更信息

| 序号 | 股东 | 变更前股权比例 | 变更后股权比例 | 股权变更日期 |
|---|---|---|---|---|
| 暂无股权变更信息 | | | | |

共 查询到 0 条记录 共 0 页 首页 上一页 下一页 末页

社保信息

| | | | |
|---|---|---|---|
| 城镇职工基本养老保险 | 86人 | 失业保险 | 85人 |
| 职工基本医疗保险 | 86人 | 工伤保险 | 88人 |
| 生育保险 | 86人 | | |

| | | |
|---|---|---|
| 单位缴费基数 | 单位参加城镇职工基本养老保险缴费基数 | 企业选择不公示 |
| | 单位参加失业保险缴费基数 | 企业选择不公示 |
| | 单位参加职工基本医疗保险缴费基数 | 企业选择不公示 |
| | 单位参加生育保险缴费基数 | 企业选择不公示 |
| 本期实际缴费金额 | 参加城镇职工基本养老保险本期实际缴费基数 | 企业选择不公示 |
| | 参加失业保险本期实际缴费基数 | 企业选择不公示 |
| | 参加职工基本医疗保险本期实际缴费基数 | 企业选择不公示 |
| | 参加工伤保险本期实际缴费基数 | 企业选择不公示 |
| | 参加生育保险本期实际缴费基数 | 企业选择不公示 |
| 单位累计欠缴金额 | 单位参加城镇职工基本养老保险累计欠缴金额 | 企业选择不公示 |
| | 单位参加失业保险累计欠缴金额 | 企业选择不公示 |
| | 单位参加职工基本医疗保险累计欠缴金额 | 企业选择不公示 |
| | 单位参加工伤保险累计欠缴金额 | 企业选择不公示 |
| | 单位参加生育保险累计欠缴金额 | 企业选择不公示 |

修改信息

| 序号 | 修改事项 | 修改前 | 修改后 | 修改日期 |
|---|---|---|---|---|
| 暂无修改信息 | | | | |

共 查询到 0 条记录 共 0 页 首页 上一页 下一页 末页

宜宾百草堂中药饮片有限公司 5105025048835

# 药品生产许可证

企业名称：四川圣上大健康药业有限公司

社会信用代码：91511621MA62B0LJ9L

注册地址：四川省广安市岳池县九龙镇城南工业园

法定代表人：刘奇志

企业负责人：李建波

质量负责人：张海

许可证编号：川20160181

分类码：Ay

日常监督管理机构：四川省药品监管局第四检查分局

投诉举报电话：12345

发证机关：四川省药品监督管理局

签发人：曾[illegible]

**生产地址和生产范围：**

岳池县九龙镇城南工业园：毒性饮片，中药饮片***

有效期至 2025 年 12 月 29 日

2020 年 12 月 30 日

国家药品监督管理局监制

# 营业执照

（副 本）

统一社会信用代码 91511621MA62H0LJ9L

| | |
|---|---|
| 名　　称 | 四川圣上大健康药业有限公司 |
| 类　　型 | 其他有限责任公司 |
| 住　　所 | 四川省广安市岳池县九龙镇城北工业园 |
| 法定代表人 | 刘奇志 |
| 注册资本 | 贰仟万元整 |
| 成立日期 | 2013年07月25日 |
| 营业期限 | 2013年07月25日　至　长期 |
| 经营范围 | 生产中药饮片（净制、切制、炒制、炙制、煅制、制炭、蒸制、煮制、炖制、焯制、酒制、醋制、盐制、姜汁炙、蜜炙、煨制、油炙）、毒性饮片（净制、切制、炒制、炙制、蒸制、煮制、醋制）；销售自产产品（有效期限以药品生产许可证为准）；种植、收购、销售中药材（国家禁止的除外）；动、植物提取物研发及相关技术转让；药品、食品研发及相关技术转让（以上经营范围国家禁止或限制的除外）。（依法须经批准的项目，经相关部门批准后方可开展经营活动）*** |

请于每年1月1日至6月30日报送、
公司年报，股权变更，企业行政许可、
企业分支机构等信息应产生后
应在20个工作日内公示。

登记机关　岳池县工商质量监督局

2017 年 10 月 [illegible] 日

http://www.gsxt.gov.cn

企业信用信息公示系统网址

中华人民共和国国家工商行政管理总局监制
