# Supplementary material for: Association of postoperative modified Yaotong Tang with early recovery after unilateral biportal endoscopy for lumbar disc herniation: a retrospective comparative cohort study using propensity score weighting
Source: Front Pharmacol. 2026 Jul 9;17:1852732. doi: 10.3389/fphar.2026.1852732 (PMC13391915; doi:10.3389/fphar.2026.1852732)
Supplement: Supplementary file 5 [file Table2.docx]

# STROBE Checklist - Cohort Study (Submission Support)

This checklist maps major STROBE domains to the revised manuscript. This checklist is provided to support review of the revised retrospective cohort manuscript.

| STROBE domain | Item | Manuscript location | Status |
| --- | --- | --- | --- |
| Title/Abstract | Title and Abstract | Title page and Abstract | Addressed; title states retrospective comparative cohort and IPTW. |
| Introduction | Background/Rationale/Objectives | Introduction | Addressed. |
| Methods | Study design, setting, participants, variables, data sources, bias, study size, quantitative variables, statistical methods | Materials and Methods 2.1-2.10 | Addressed; see also Supplementary Tables S1-S8. |
| Results | Participants, descriptive data, outcome data, main results, other analyses | Results 3.1-3.6 | Addressed. |
| Discussion | Key results, limitations, interpretation, generalizability | Discussion 4.1-4.7 | Addressed; limitations include residual confounding and incomplete historical COA for non-aconite components. |
| Other information | Funding and ethics | Funding/Ethics/Data Availability/COI/Acknowledgments | Addressed; author should confirm system entries match manuscript. |
