# Supplementary material for: Association of postoperative modified Yaotong Tang with early recovery after unilateral biportal endoscopy for lumbar disc herniation: a retrospective comparative cohort study using propensity score weighting
Source: Front Pharmacol. 2026 Jul 9;17:1852732. doi: 10.3389/fphar.2026.1852732 (PMC13391915; doi:10.3389/fphar.2026.1852732)
Supplement: Supplementary file 6 [file DataSheet1.pdf]

Supplementary Tables S1-S11

All tables are presented in English for journal submission. Original Chinese source documents are indexed in the supplementary data sheets and should be used only as supporting evidence.

Supplementary Table S1. Pharmacopoeial identity, manufacturer, batch information, and quality-control documentation of MYT components.

Component	Botanical source / family	Medicinal part / processing	Daily dose	Batch No.	Manufacturer	COA / inspection report No.	Evidence page	Validity	Conclusion	Patient coverage
Dipsaci Radix	Dipsacus asper Wall. ex Henry [Caprifoliaceae]	Dried root; decoction piece	24 g	220112-1	Luzhou Baicaotang Chinese Herbal Decoction Pieces Co., Ltd.	C-22-011216	20	Valid through 2027	Compliant	MYT-001–MYT-062
Poria	Poria cocos (Schw.) Wolf [Polyporaceae]	Dried sclerotium; decoction piece	15 g	220117-1	Luzhou Baicaotang Chinese Herbal Decoction Pieces Co., Ltd.	C-22-011713	19	Valid through 2027	Compliant	MYT-001–MYT-062
Zingiberis Rhizoma	Zingiber officinale Roscoe [Zingiberaceae]	Dried rhizome; decoction piece	15 g	220310-1	Luzhou Baicaotang Chinese Herbal Decoction Pieces Co., Ltd.	C-22-031014	18	Valid through 2027	Compliant	MYT-001–MYT-062
Atractylodis Macrocephalae Rhizoma	Atractylodes macrocephala Koidz. [Asteraceae]	Dried rhizome; decoction piece	20 g	220124-2	Luzhou Baicaotang Chinese Herbal Decoction Pieces Co., Ltd.	C-22-012413	16	Valid through 2027	Compliant	MYT-001–MYT-062
Glycyrrhizae Radix et Rhizoma Praeparata cum Melle	Glycyrrhiza uralensis Fisch./G. inflata Bat./G. glabra L. [Fabaceae]	Root/rhizome; honey-processed decoction piece	12 g	220112-1	Luzhou Baicaotang Chinese Herbal Decoction Pieces Co., Ltd.	C-22-011216	14	Valid through 2027	Compliant	MYT-001–MYT-062
Citri Reticulatae Pericarpium	Citrus reticulata Blanco [Rutaceae]	Mature fruit peel; decoction piece	20 g	220119-1	Luzhou Baicaotang Chinese Herbal Decoction Pieces Co., Ltd.	C-22-011910	17	Valid through 2027	Compliant	MYT-001–MYT-062
Aconiti Radix Cocta	Aconitum carmichaelii Debeaux [Ranunculaceae]	Processed daughter root; pre-decocted separately	9 g	211101	Sichuan Shengshang Da Health Pharmaceutical Co., Ltd.	JBCA06708211101	12	2021/2022 source record; see report	Compliant / in accordance with Chinese Pharmacopoeia	MYT-001-MYT-030
Aconiti Radix Cocta	Aconitum carmichaelii Debeaux [Ranunculaceae]	Processed daughter root; pre-decocted separately	9 g	221026-1	Luzhou Baicaotang Chinese Herbal Decoction Pieces Co., Ltd.	C-22-102624	10	2022/2027 source record; see report	Compliant / in accordance with Chinese Pharmacopoeia	MYT-031-MYT-062

COA, certificate of analysis; MYT, Modified Yaotong Tang. Chinese source reports are provided in Supplementary Data Sheet 2.

Supplementary Table S1A. Patient-level matching summary of Aconiti Radix Cocta batches in the UBE+MYT cohort.

Patient range	First-administration window	Aconiti Radix Cocta batch No.	Manufacturer	COA / inspection report No.	Evidence page	Coverage status	Safety-specific traceability
MYT-001 to MYT-030	POD0 evening to POD2 morning according to patient records	Batch 211101	Sichuan Shengshang Da Health Pharmaceutical Co., Ltd.	JBCA06708211101	12	Covered	No documented cardiovascular or neurologic symptom suggestive of aconite-related toxicity
MYT-031 to MYT-062	POD0 evening to POD2 morning according to patient records	Batch 221026-1	Luzhou Baicaotang Chinese Herbal Decoction Pieces Co., Ltd.	C-22-102624	10	Covered	No documented cardiovascular or neurologic symptom suggestive of aconite-related toxicity

All 62 UBE+MYT patients were matched to documented Aconiti Radix Cocta batches. The detailed de-identified patient-level source records were retained by the study team.

Supplementary Table S1B. COA / inspection-report coverage index for clinically used MYT batches.

Component	Batch No.	Manufacturer	COA / inspection report No.	Evidence page	Validity	Inspection conclusion	Actual clinical batch	Patient coverage	Comments
Dipsaci Radix	220112-1	Luzhou Baicaotang Chinese Herbal Decoction Pieces Co., Ltd.	C-22-011216	20	Valid through 2027	Compliant	Yes	MYT-001–MYT-062	Covered by available COA/inspection report.
Poria	220117-1	Luzhou Baicaotang Chinese Herbal Decoction Pieces Co., Ltd.	C-22-011713	19	Valid through 2027	Compliant	Yes	MYT-001–MYT-062	Covered by available COA/inspection report.
Zingiberis Rhizoma	220310-1	Luzhou Baicaotang Chinese Herbal Decoction Pieces Co., Ltd.	C-22-031014	18	Valid through 2027	Compliant	Yes	MYT-001–MYT-062	Covered by available COA/inspection report.
Atractylodis Macrocephalae Rhizoma	220124-2	Luzhou Baicaotang Chinese Herbal Decoction Pieces Co., Ltd.	C-22-012413	16	Valid through 2027	Compliant	Yes	MYT-001–MYT-062	Covered by available COA/inspection report.
Glycyrrhizae Radix et Rhizoma Praeparata cum Melle	220112-1	Luzhou Baicaotang Chinese Herbal Decoction Pieces Co., Ltd.	C-22-011216	14	Valid through 2027	Compliant	Yes	MYT-001–MYT-062	Covered by available COA/inspection report.
Citri Reticulatae Pericarpium	220119-1	Luzhou Baicaotang Chinese Herbal Decoction Pieces Co., Ltd.	C-22-011910	17	Valid through 2027	Compliant	Yes	MYT-001–MYT-062	Covered by available COA/inspection report.
Aconiti Radix Cocta	211101	Sichuan Shengshang Da Health Pharmaceutical Co., Ltd.	JBCA06708211101	12	See COA report	Compliant	Yes	MYT-001-MYT-030	Covered by patient-level matching table.
Aconiti Radix Cocta	221026-1	Luzhou Baicaotang Chinese Herbal Decoction Pieces Co., Ltd.	C-22-102624	10	See COA report	Compliant	Yes	MYT-031-MYT-062	Covered by patient-level matching table.

Supplementary Table S1C. Aconiti Radix Cocta quality-control and safety-specific evidence closure.

Verification item	Evidence source	Status	Result / evidence location	Editorial interpretation
Identity confirmation	COA reports; pharmacopoeial identity table	Completed	Both clinically used batches were documented as Aconiti Radix Cocta / processed Zhi Chuan Wu, not raw Chuan Wu.	Supports botanical/pharmacopoeial identity and safety-specific reporting.
Patient-level batch coverage	Supplementary Table S1A	Completed	62/62 UBE+MYT patients matched to Aconiti Radix Cocta batches.	Supports statement of complete Aconiti patient-level traceability.
COA / inspection-report coverage	Supplementary Table S1B; Data Sheet 2	Completed	Batch 211101: report JBCA06708211101, p.12; batch 221026-1: report C-22-102624, p.10.	Supports ConPhyMP manufacturer/COA requirement for safety-critical component.
Toxic-decoction-piece qualification	Manufacturer license/qualification documents; Data Sheet 3	Completed	Manufacturer qualification documents collated; author should verify license number, validity, and production scope against original scans.	Supports regulatory-status reporting; final legal details require author confirmation.
Aconitine-type alkaloid / safety-related testing	COA reports; Data Sheet 2; Supplementary Table S11	Completed	Available COA reports include diester-type alkaloid testing and content determination; both clinically used batches met reported specifications.	Supports safety-related QC marker reporting; does not prove clinical safety.
Pre-decoction procedure	Institutional decoction SOP; Supplementary Table S2	Completed	Processed Zhi Chuan Wu was separately pre-decocted according to the institutional decoction procedure before combination with other herbs.	Supports reproducibility and safety transparency.
Aconiti-related adverse-event review	Safety verification tables; Supplementary Table S6	Completed	No clear cardiovascular or neurologic toxicity signal suggestive of aconite-related toxicity was documented in the UBE+MYT cohort.	Supports “no severe safety signal observed,” not proof of absolute safety.
Overall conclusion	S1A + S1B + S1C + COA reports	Completed	Patient-level Aconiti Radix Cocta traceability and inspection-report coverage were verified for all 62 UBE+MYT patients.	Use cautiously in manuscript; do not claim complete prospective pharmaceutical standardization.

This table addresses the safety-critical aconite-containing component. COA compliance does not establish clinical safety; clinical safety was evaluated separately through adverse-event review.

Supplementary Table S2. Institutional decoction and traceability procedure for MYT.

Procedure domain	Institutional procedure / verification item	Traceability source	Relevance to reviewer concern
Prescription review	Pharmacists verified patient information, herbal names, daily doses, processing status, batch information, expiry dates, and available quality-control documents before decoction.	Hospital pharmacy prescription review and dispensing records	Supports reproducibility and prescription-level traceability.
Aconiti Radix Cocta handling	Processed Zhi Chuan Wu was verified as Aconiti Radix Cocta and was not substituted by raw Chuan Wu; it was weighed and labelled separately.	Hospital decoction-room SOP and pharmacy records	Addresses safety-critical processing of aconite-containing component.
Pre-decoction	Aconiti Radix Cocta 9 g was pre-decocted separately for approximately 60 min before combination with the remaining herbs.	Hospital decoction-room SOP	Supports mitigation of aconite-related safety risk.
Combined decoction	The remaining six herbal components were soaked before decoction and then combined with the pre-decocted Aconiti Radix Cocta for routine two-step decoction.	Hospital decoction-room SOP	Supports repeatability of extraction/decoction procedure.
Concentration and packaging	Combined filtrates were concentrated to approximately 400 mL per daily dose and divided into two sealed portions of approximately 200 mL each.	Hospital decoction-room packaging records	Supports administration consistency.
Storage and dispensing	Sealed decoctions not taken immediately were stored according to pharmacy requirements; dispensing and administration records were retained by the hospital.	Pharmacy/decoction-room and nursing administration records	Supports clinical exposure verification.
Administration schedule	MYT was administered orally in two divided warm doses in the morning and evening for a planned 7-day postoperative course.	Electronic medical orders, nursing records, follow-up records	Clarifies treatment timing and duration.

Supplementary Table S3. MYT initiation timing and treatment-course adherence in the UBE+MYT cohort.

Item	Result	Comment
Number of UBE+MYT patients	62/62	All included patients with documented MYT exposure
Planned regimen	One daily decoction dose for 7 postoperative days, divided into morning and evening administrations	Planned total: 14 administrations
First administration: POD0 evening	38/62	Verified from medical, pharmacy/decoction-room, nursing, and follow-up records
First administration: POD1 morning	21/62	Verified
First administration: POD1 evening	2/62	Verified
First administration: POD2 morning	1/62	Verified
Completed 14/14 administrations	52/62	Full planned course
Completed 13/14 administrations	4/62	Minor omission documented
Completed 12/14 administrations	3/62	Minor omission documented
Completed 10-11/14 administrations	3/62	Shortened course documented
Aconiti Radix Cocta-specific symptoms	0/62	No palpitations, arrhythmia, chest tightness, hypotension, perioral numbness, limb numbness, or tremor documented

Supplementary Table S4. Standard perioperative pathway and co-interventions in the two UBE cohorts.

Domain	UBE+MYT cohort	UBE-alone cohort	Consistency
Surgical team	Same senior spinal surgery team	Same senior spinal surgery team	Yes
Anesthesia	General anesthesia	General anesthesia	Yes
Surgical procedure	UBE decompression	UBE decompression	Yes
Preoperative fasting	Institutional anesthesia protocol	Institutional anesthesia protocol	Yes
Routine analgesia	Institutional analgesic pathway	Institutional analgesic pathway	Yes
Rescue analgesia	Triggered by VAS and clinical need	Triggered by VAS and clinical need	Yes
Antibiotic prophylaxis	Institutional prophylactic antibiotic protocol	Institutional prophylactic antibiotic protocol	Yes
Systemic corticosteroids	Not routinely used postoperatively	Not routinely used postoperatively	Yes
Anticoagulant/hemostatic drugs	Not routinely used; risk-based if clinically indicated	Not routinely used; risk-based if clinically indicated	Yes
Rehabilitation	Standard early mobilization and brace-protected activity	Standard early mobilization and brace-protected activity	Yes
Laboratory monitoring	Preoperative, POD1, POD3, POD7	Preoperative, POD1, POD3, POD7	Yes
Discharge criteria	Unified institutional criteria	Unified institutional criteria	Yes
MYT exposure	MYT administered as adjunctive treatment	No MYT and no placebo	No; study exposure
Routine early analgesia	Parecoxib sodium; 40 mg intravenously every 12 h; POD0-POD2 or until oral transition	Parecoxib sodium; 40 mg intravenously every 12 h; POD0-POD2 or until oral transition	Yes
Oral transition analgesia	Celecoxib; 200 mg orally twice daily; From POD2 when tolerated, generally to POD5-POD7	Celecoxib; 200 mg orally twice daily; From POD2 when tolerated, generally to POD5-POD7	Yes
Gastroprotection	Proton-pump inhibitor; Institutional standard dose; During NSAID/COX-2 use as clinically indicated	Proton-pump inhibitor; Institutional standard dose; During NSAID/COX-2 use as clinically indicated	Yes
Rescue analgesia	Tramadol; 50 mg orally or intramuscularly every 8 h as needed; VAS >=4 or breakthrough pain	Tramadol; 50 mg orally or intramuscularly every 8 h as needed; VAS >=4 or breakthrough pain	Yes
Strong opioids	Not routinely used; None in the UBE analytic cohorts; Not applicable	Not routinely used; None in the UBE analytic cohorts; Not applicable	Yes
Antibiotic prophylaxis	Cefuroxime sodium; 1.5 g IV 30-60 min before incision; then every 12 h, total duration <=24 h; Used in both groups according to the same protocol	Cefuroxime sodium; 1.5 g IV 30-60 min before incision; then every 12 h, total duration <=24 h; Used in both groups according to the same protocol	Yes
Alternative for cephalosporin allergy	Clindamycin; 600 mg IV according to the institutional antimicrobial protocol; Used only when clinically indicated	Clindamycin; 600 mg IV according to the institutional antimicrobial protocol; Used only when clinically indicated	Yes
Systemic corticosteroids	Methylprednisolone/dexamethasone; Not routinely used as postoperative anti-inflammatory treatment; No routine postoperative systemic corticosteroid regimen	Methylprednisolone/dexamethasone; Not routinely used as postoperative anti-inflammatory treatment; No routine postoperative systemic corticosteroid regimen	Yes
Anticoagulants	Low-molecular-weight heparin; Not routinely used after UBE; used only after individual VTE-risk evaluation; No routine use in the analytic UBE cohorts	Low-molecular-weight heparin; Not routinely used after UBE; used only after individual VTE-risk evaluation; No routine use in the analytic UBE cohorts	Yes
Hemostatic agents	Tranexamic acid/hemostatic agents; Not routinely used after UBE; No routine use in the analytic UBE cohorts	Tranexamic acid/hemostatic agents; Not routinely used after UBE; No routine use in the analytic UBE cohorts	Yes
Mechanical VTE prevention	Ankle-pump exercise and early mobilization; Started after recovery from anesthesia; Used in both groups	Ankle-pump exercise and early mobilization; Started after recovery from anesthesia; Used in both groups	Yes
Other herbal formulas or Chinese patent medicines	None routinely co-administered; Not routinely combined during the perioperative observation window; None documented as routine co-intervention	None routinely co-administered; Not routinely combined during the perioperative observation window; None documented as routine co-intervention	Yes

Routine analgesic and concomitant-intervention criteria were the same across UBE cohorts; observed rescue analgesia use is summarized below.

Indicator	UBE+MYT (n=62)	UBE alone (n=60)
Routine COX-2-based analgesia	62/62 (100.0%)	60/60 (100.0%)
Rescue analgesia use	8/62 (12.9%)	17/60 (28.3%)
Strong opioid use	0/62 (0.0%)	0/60 (0.0%)

Analgesic discontinuation due to intolerance	0/62 (0.0%)	1/60 (1.7%)

Supplementary Table S5. Laboratory sampling schedule and completeness.

Time point	Sampling window	Tests	Notes
Preoperative baseline	Within 24 h before surgery, usually 06:00-08:00	IL-6, CRP, D-dimer, liver/renal function and routine perioperative labs	Baseline before surgical exposure
POD1	06:00-08:00 on postoperative day 1	IL-6, CRP, D-dimer	Morning sampling before oral MYT/analgesic administration when applicable
POD3	06:00-08:00 on postoperative day 3	IL-6, CRP, D-dimer	Same time window
POD7	06:00-08:00 on postoperative day 7	IL-6, CRP, D-dimer	Same time window; inpatient or scheduled review sampling
Indicator	UBE+MYT (n=62)	UBE alone (n=60)	Consistency
Preoperative baseline sampling complete	62/62	60/60	Yes
POD1 sampling complete	62/62	60/60	Yes
POD3 sampling complete	62/62	60/60	Yes
POD7 sampling complete	62/62	60/60	Yes
Morning sampling window	06:00-08:00	06:00-08:00	Yes

Supplementary Table S6. Adverse-event adjudication and safety summary.

Study ID	Group	Event	Onset	Grade	Management	Outcome	Readmission / reoperation	Relationship to MYT	Zhi Chuan Wu symptoms
MYT-AE01	UBE+MYT	Transient nausea/vomiting	POD1	Grade I	Temporary oral intake delay, symptomatic antiemetic treatment and observation	Resolved on POD2	No/No	Uncertain; anesthesia or analgesic medication more likely	No palpitations, arrhythmia, chest tightness, hypotension, perioral numbness, limb numbness, or tremor
MYT-AE02	UBE+MYT	Mild diarrhea	POD3	Grade I	Dietary adjustment, oral hydration/observation; no antibiotic escalation	Resolved on POD4	No/No	Possible	No palpitations, arrhythmia, chest tightness, hypotension, perioral numbness, limb numbness, or tremor
UBE-AE01	UBE alone	Postoperative nausea/vomiting	POD1	Grade I	Symptomatic antiemetic treatment and observation	Resolved on POD2	No/No	Not applicable	Not applicable
UBE-AE02	UBE alone	Dizziness/orthostatic discomfort	POD1	Grade I	Rest, hydration, and observation	Resolved on POD2	No/No	Not applicable	Not applicable
UBE-AE03	UBE alone	Mild wound exudation	POD2	Grade I	Enhanced wound dressing; no antibiotic escalation	Improved by POD4	No/No	Not applicable	Not applicable
UBE-AE04	UBE alone	Constipation	POD3	Grade I	Dietary guidance and laxative management	Resolved by POD5	No/No	Not applicable	Not applicable
UBE-AE05	UBE alone	Transient fever	POD2	Grade I	Physical cooling and observation; no infection focus identified	Resolved on POD3	No/No	Not applicable	Not applicable
UBE-AE06	UBE alone	Transient urinary retention	POD1	Grade I	Intermittent catheterization once and observation	Spontaneous urination recovered on POD2	No/No	Not applicable	Not applicable
UBE-AE07	UBE alone	Urinary tract infection	POD4	Grade II	Oral antibiotic therapy	Improved by POD7	No/No	Not applicable	Not applicable
UBE-AE08	UBE alone	Superficial wound infection	POD5	Grade II	Oral antibiotic therapy and enhanced wound care	Healed within 2 weeks	No/No	Not applicable	Not applicable
UBE-AE09	UBE alone	Moderate gastrointestinal reaction	POD2	Grade II	NSAID adjustment, gastroprotection and antiemetic medication	Resolved by POD4	No/No	Not applicable	Not applicable

Safety indicator	UBE+MYT (n=62)	UBE alone (n=60)
Any postoperative adverse event	2/62 (3.2%)	9/60 (15.0%)
Clavien-Dindo Grade I	2/62 (3.2%)	6/60 (10.0%)
Clavien-Dindo Grade II	0/62 (0.0%)	3/60 (5.0%)
Clavien-Dindo Grade >=III	0/62 (0.0%)	0/60 (0.0%)
Readmission	0/62 (0.0%)	0/60 (0.0%)
Reoperation	0/62 (0.0%)	0/60 (0.0%)
Severe hepatic or renal dysfunction	0/62 (0.0%)	0/60 (0.0%)
Allergic reaction	0/62 (0.0%)	0/60 (0.0%)
Cardiovascular/neurologic symptoms suggestive of Zhi Chuan Wu toxicity	0/62 (0.0%)	Not applicable

Supplementary Table S7. IPTW diagnostics, covariate balance, and effective sample size.

Covariate	Type	SMD before	SMD after IPTW	SMD after overlap weighting
Age	Continuous	0.061	0.002	0.000
Male sex	Binary	0.133	0.014	0.000
Preoperative VAS	Continuous	0.128	0.003	0.000
Preoperative JOA	Continuous	0.112	0.011	0.000
Preoperative TCM syndrome score	Continuous	0.089	0.005	0.000
Preoperative CRP (mg/L)	Continuous	0.113	0.007	0.000
Preoperative D-dimer (ug/mL)	Continuous	0.015	0.011	0.000
Preoperative IL-6 (pg/mL)	Continuous	0.005	0.004	0.000
Preoperative disc height (mm)	Continuous	0.315	0.008	0.000
Preoperative spinal canal volume	Continuous	0.158	0.014	0.000
Herniation level: Upper lumbar	Binary	0.068	0.006	0.000
Herniation level: L4/5	Binary	0.141	0.014	0.000
Herniation level: L5/S1	Binary	0.191	0.012	0.000

Diagnostic item	Value	Interpretation
n in UBE analytic cohort	122	62 UBE+MYT and 60 UBE-alone patients
Maximum SMD before IPTW	0.315	Largest baseline imbalance before weighting
Maximum SMD after IPTW	0.014	All covariates below 0.10 after weighting
Maximum stabilized weight	2.064	No problematic extreme weights observed
Weights > 10	0	No extreme weights above 10
Effective sample size overall	113.902	Preserved most of the original sample size
ESS UBE+MYT	57.981	Treatment group ESS
ESS UBE alone	55.921	Control group ESS
Matched pairs in PSM sensitivity analysis	43	Directionally consistent with primary IPTW

Supplementary Table S8. Sensitivity analyses for main early recovery outcomes.

Outcome	Method	Estimate	95% CI	P value	Direction consistent	Matched pairs
VAS POD3	Main stabilized IPTW	-0.792	-1.061 to -0.522	8.42e-09	Yes	NA
VAS POD3	Trimmed IPTW (1st/99th percentile)	-0.794	-1.063 to -0.525	7.08e-09	Yes	NA
VAS POD3	Overlap weighting	-0.813	-1.081 to -0.546	2.49e-09	Yes	NA
VAS POD3	1:1 propensity-score matching	-0.930	-1.248 to -0.612	5.41e-07	Yes	43
VAS POD3	Multivariable regression adjustment	-0.815	-1.106 to -0.525	3.85e-08	Yes	NA
IL-6 POD3	Main stabilized IPTW	-11.515	-13.121 to -9.909	7.16e-45	Yes	NA
IL-6 POD3	Trimmed IPTW (1st/99th percentile)	-11.533	-13.137 to -9.929	3.99e-45	Yes	NA
IL-6 POD3	Overlap weighting	-11.557	-13.176 to -9.939	1.68e-44	Yes	NA
IL-6 POD3	1:1 propensity-score matching	-11.309	-13.283 to -9.336	1.22e-14	Yes	43
IL-6 POD3	Multivariable regression adjustment	-11.552	-13.270 to -9.834	1.13e-39	Yes	NA
VAS POD7	Main stabilized IPTW	-0.882	-1.147 to -0.617	6.4e-11	Yes	NA
VAS POD7	Trimmed IPTW (1st/99th percentile)	-0.881	-1.146 to -0.616	6.89e-11	Yes	NA
VAS POD7	Overlap weighting	-0.891	-1.151 to -0.631	1.85e-11	Yes	NA
VAS POD7	1:1 propensity-score matching	-0.837	-1.105 to -0.569	1.45e-07	Yes	43
VAS POD7	Multivariable regression adjustment	-0.892	-1.169 to -0.616	2.38e-10	Yes	NA
IL-6 POD7	Main stabilized IPTW	-5.621	-6.413 to -4.828	6.77e-44	Yes	NA
IL-6 POD7	Trimmed IPTW (1st/99th percentile)	-5.623	-6.418 to -4.828	1e-43	Yes	NA
IL-6 POD7	Overlap weighting	-5.605	-6.401 to -4.808	2.87e-43	Yes	NA
IL-6 POD7	1:1 propensity-score matching	-5.442	-6.352 to -4.531	3.13e-15	Yes	43
IL-6 POD7	Multivariable regression adjustment	-5.599	-6.458 to -4.740	2.35e-37	Yes	NA
CRP POD3	Main stabilized IPTW	-5.331	-7.238 to -3.424	4.28e-08	Yes	NA
CRP POD3	Trimmed IPTW (1st/99th percentile)	-5.327	-7.236 to -3.418	4.51e-08	Yes	NA
CRP POD3	Overlap weighting	-5.395	-7.316 to -3.474	3.71e-08	Yes	NA
CRP POD3	1:1 propensity-score matching	-5.539	-7.906 to -3.171	2.62e-05	Yes	43
CRP POD3	Multivariable regression adjustment	-5.398	-7.375 to -3.421	8.74e-08	Yes	NA
D-dimer POD3	Main stabilized IPTW	-0.887	-1.109 to -0.665	5.29e-15	Yes	NA
D-dimer POD3	Trimmed IPTW (1st/99th percentile)	-0.887	-1.109 to -0.664	6.18e-15	Yes	NA
D-dimer POD3	Overlap weighting	-0.896	-1.126 to -0.667	1.79e-14	Yes	NA
D-dimer POD3	1:1 propensity-score matching	-0.904	-1.203 to -0.606	2.73e-07	Yes	43
D-dimer POD3	Multivariable regression adjustment	-0.897	-1.140 to -0.653	5.62e-13	Yes	NA
JOA 1 month	Main stabilized IPTW	1.598	1.248 to 1.947	3.52e-19	Yes	NA
JOA 1 month	Trimmed IPTW (1st/99th percentile)	1.601	1.251 to 1.950	2.88e-19	Yes	NA
JOA 1 month	Overlap weighting	1.616	1.270 to 1.961	5.38e-20	Yes	NA
JOA 1 month	1:1 propensity-score matching	1.651	1.278 to 2.024	2.96e-11	Yes	43
JOA 1 month	Multivariable regression adjustment	1.613	1.233 to 1.994	9.9e-17	Yes	NA

Supplementary Table S9. Concise summary of annotated constituents in the MYT sample.

A total of 187 constituents were annotated in the MYT sample by UHPLC-Q-Orbitrap HRMS. To maintain readability, this table provides a concise class-level summary and representative annotated constituents; the complete original annotation output is provided in Supplementary Data Sheet 5.

Chemical class			Number of annotated constituents		
Prenol lipids			60		
Flavonoids			52		
Organooxygen compounds			15		
Isoflavonoids			11		
Phenols			8		
Other annotated classes			41		
Representative constituent	Formula	Chemical class	Assigned source component(s)	Reference-substance assisted	Retention time (min)
Loganic acid	C16H24O10	Prenol lipids	Dipsaci Radix	Yes	6.86
Hesperidin	C28H34O15	Flavonoids	Citri Reticulatae Pericarpium,Dipsaci Radix,Atractylodis Macrocephalae Rhizoma	Yes	12.98
Glycyrrhizic acid	C42H62O16	Prenol lipids	Glycyrrhizae Radix et Rhizoma Praeparata cum Melle	Yes	18.99
Neoliquiritin	C21H22O9	Flavonoids	Citri Reticulatae Pericarpium	Unassigned	10.90
6-Gingerol	C17H26O4	Phenols	Zingiberis Rhizoma	Yes	19.51
Magnoflorine	C20H24NO4+	Aporphines	Aconiti Radix Cocta,Dipsaci Radix,Atractylodis Macrocephalae Rhizoma,Citri Reticulatae Pericarpium	Yes	8.80
Aconitine	C34H47NO11	Prenol lipids	Aconiti Radix Cocta	Yes	17.19
Hypaconitine	C33H45NO10	Prenol lipids	Aconiti Radix Cocta	Yes	17.19
Benzoylmesaconine	C31H43NO10	Prenol lipids	Aconiti Radix Cocta	Yes	13.43
Asperosaponin VI	C47H76O18	Prenol lipids	Dipsaci Radix,Citri Reticulatae Pericarpium	Yes	17.31
Nobiletin	C21H22O8	Flavonoids	Citri Reticulatae Pericarpium,Dipsaci Radix	Yes	19.56
Higenamine	C16H17NO3	Isoquinolines and derivatives	Aconiti Radix Cocta	Yes	6.07

Supplementary Table S10. Concise summary of putative MYT-related constituents and metabolites detected in medicated serum.

A total of 75 putative MYT-related serum features were detected, including 22 prototype constituents and 53 metabolites. Full annotation details are provided in Supplementary Data Sheet 5.

Type	Number of detected features
Prototype constituents	22
Metabolites	53

Assigned source component	Number of serum features
Citri Reticulatae Pericarpium	23
Glycyrrhizae Radix et Rhizoma Praeparata cum Melle	19
Aconiti Radix Cocta	15
Dipsaci Radix	14
Zingiberis Rhizoma	11
Atractylodis Macrocephalae Rhizoma	10
Poria	4
Formula-related or non-herb-specific feature	1

Representative prototype constituent	Formula	Detection mode	Retention time (min)	Assigned source component(s)
Higenamine	C16H17NO3	POS	6.04	Aconiti Radix Cocta
Loganic acid	C16H24O10	POS	7.17	Dipsaci Radix
tecomoside	C16H24O10	NEG	7.26	Dipsaci Radix
Magnoflorine	C20H24NO4+	POS	8.77	Aconiti Radix Cocta,Dipsaci Radix,Atractylodis Macrocephalae Rhizoma,Citri Reticulatae Pericarpium
Sweroside	C16H22O9	POS	8.98	Dipsaci Radix
Loganin	C17H26O10	POS	9.05	Dipsaci Radix
4-O-Feruloylquinic acid	C17H20O9	POS	9.62	Atractylodis Macrocephalae Rhizoma,Citri Reticulatae Pericarpium
Talatisamine	C24H39NO5	POS	9.67	Aconiti Radix Cocta
Vitexin	C21H20O10	POS	10.82	Citri Reticulatae Pericarpium
picrocrocinic acid	C16H26O8	NEG	11.15	Citri Reticulatae Pericarpium
Narcissoside	C28H32O16	POS	12.37	Citri Reticulatae Pericarpium
6,7,4'-Trihydroxyflavanone	C15H12O5	POS	12.57	Glycyrrhizae Radix et Rhizoma Praeparata cum Melle

Supplementary Table S11. Marker and safety-related quality-control quantification for Aconiti Radix Cocta.

Component	Batch No.	Manufacturer	Marker / QC item	Method/source	Reported result	Specification / limit	Conclusion	Evidence page	Interpretation
Aconiti Radix Cocta	221026-1	Luzhou Baicaotang Chinese Herbal Decoction Pieces Co., Ltd.	Diester-type alkaloids	Batch COA / Chinese Pharmacopoeia method	0.002%	NMT 0.040%	Compliant	10	Safety-related QC marker; author should verify numeric value against original COA.
Aconiti Radix Cocta	221026-1	Luzhou Baicaotang Chinese Herbal Decoction Pieces Co., Ltd.	Content determination item	Batch COA / Chinese Pharmacopoeia method	0.082%	0.070%-0.150%	Compliant	10	Quality-control marker; author should verify numeric value against original COA.
Aconiti Radix Cocta	211101	Sichuan Shengshang Da Health Pharmaceutical Co., Ltd.	Diester-type alkaloids	Batch COA / Chinese Pharmacopoeia method	0.007%	NMT 0.040%	Compliant	12	Safety-related QC marker; author should verify numeric value against original COA.
Aconiti Radix Cocta	211101	Sichuan Shengshang Da Health Pharmaceutical Co., Ltd.	Content determination item	Batch COA / Chinese Pharmacopoeia method	0.13%	0.070%-0.150%	Compliant	12	Quality-control marker; author should verify numeric value against original COA.

NMT, not more than. These values are safety-related and quality-control markers from batch COA reports; authors should verify numeric values against original COA pages before submission.
