# Supplementary material for: Association of postoperative modified Yaotong Tang with early recovery after unilateral biportal endoscopy for lumbar disc herniation: a retrospective comparative cohort study using propensity score weighting
Source: Front Pharmacol. 2026 Jul 9;17:1852732. doi: 10.3389/fphar.2026.1852732 (PMC13391915; doi:10.3389/fphar.2026.1852732)

Supplementary Data Sheet 5. Phytochemical Characterization and In Vivo Constituent Profiling

English index for the LC-HRMS phytochemical characterization package. The following pages support chemical profiling and putative exposure documentation only.

Evidence page	Material	Analysis	Output	Related tables	Interpretation
Following pages	MYT sample	UHPLC-Q-Orbitrap HRMS	Annotated constituents; positive/negative ion BPI figures	Supplementary Tables S9-S10	Chemical characterization, not proof of pharmacodynamic mechanism
Following pages	Medicated serum / blank serum	UHPLC-Q-Orbitrap HRMS	Putative in vivo constituents/metabolites; BPI figures	Supplementary Table S10	Putative exposure documentation

中药及其体内成分分析报告

Chemical Analysis Report of Traditional Chinese Medicine and Its In Vivo Components

项目编号： HX250901-6ZYFF

委 托 方： 西南医科大学附属中医医院-陈光友

受 托 方： 宁波慧均生物

目录

1 方案概述......1

2 药味信息......2

3 仪器与试剂......2

3.1 仪器......2

3.2 试剂......3

3.3 实验方法......3

3.3.1 样品制备......4

3.3.2 色谱方法......4

3.3.3 质谱方法......5

3.4 数据分析......6

4 实验结果......6

4.1 谱图......6

4.2 鉴定结果......8

5 参考资料......8

6 参考文献......8

1 方案概述

中医药是中华民族五千年文明的结晶，是浩瀚历史长河中人民生命健康的守护者。特别是，新冠疫情爆发以来，中医药发挥了独特的优势和积极作用。“传承精华、守正创新”是党和国家对我们中医药行业人员的殷切嘱托[1]，而“明晰药效物质基础”是传承好、发展好中医药事业的逻辑基础，也是推进中医药现代化、产业化的必由之路。

对于具体研究来说，中药化学组成是后续开展药效物质发现（结合移行成分分析[2]、网络药理学[3]、整合药理学[4]、方证代谢组学[5]等）、验证（结合 PK/PD 分析[6]、药效分析[7]）、中药质量标志物（Q-marker）遴选[8-10]、中药药效机制探索[11]的底层数据，是中药丰富科学内涵的物质基础。此外，明晰中药化学组成也成为 Phytomedicine[12]、Journal of Ethnopharmacology[13]等中药类期刊发表文章的常规要求。

中药作为多组分复杂体系，具有成分繁多、结构多样、含量差异大、异构体丰富等特点，定性分析面临着很大的挑战。传统色谱技术（薄层色谱[14]、液相色谱[15]等）的本质是利用各成分在流动相和固定相中的分配能力差异而表现出的不同分离特性（薄层色谱为比移值 Rf = 溶质移动的距离/溶液移动的距离，气相色谱和液相色谱为保留时间 Rt）。当色谱分离条件固定时，上述分离特性为固定值，因此可通过与对照品的 Rf 或 Rt 值进行比较，判断样品是否含有某一化合物。此类方法具有设备价格相对较低、操作简单、结果易读等特点，因而广泛用于中药材及饮片、中成药、中药提取物等的质量控制[14]。然而，上述方法需要通过与对照品进行比对才可对化合物进行定性，因此，对未知成分的定性能力很差，无法满足当前全面阐明中药化学组成的需求。

2020 年，国家药监局发布《关于促进中药传承创新发展的实施意见》，明确提出要“引入新工具、新方法、新技术”[16]，深入挖掘中医药宝库的科学内涵。液相色谱-高分辨质谱联用技术不仅兼顾了色谱分离特性，还增加了质谱维度信息（母离子质荷比、子离子质荷比、同位素分布）等，极大提高了鉴定结果的准确度，避免了常规色谱定性因保留时间相同导致的假阳性；此外，可通过母离子质荷比、子离子质荷比、同位素分布相似性等质谱维度信息对无对照品的化合物进行定性，因而极大拓展了常规色谱技术对未知成分的定性能力，定性结果往往可达到 100+[17]。

基于上述认知，本项目采用超高效液相色谱-四极杆-静电场轨道阱高分辨质谱（Ultra-high performance liquid chromatography coupled with hybrid quadrupole-orbitrap high resolution mass spectrometry, UHPLC-Q-Orbitrap HRMS）技术检测样品中的中药化学成分，并通过与对

照品数据库、理论数据库比对，结合人工核验，对成分进行定性，项目流程参见图 1。

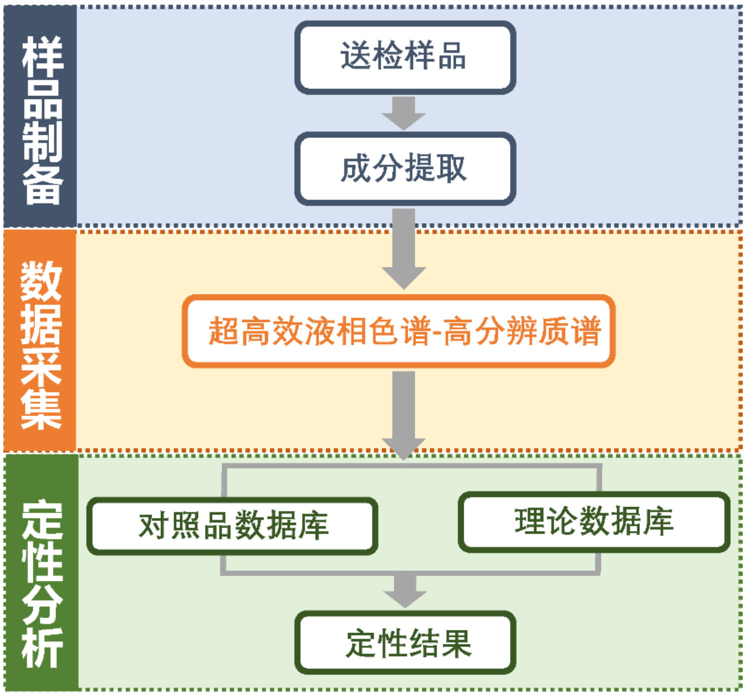

图 1 项目流程

2 药味信息

药味组成：茯苓、干姜、白术、炙甘草、川断、陈皮、制川乌

3 仪器与试剂

3 Instruments and reagents

3.1 仪器

表 1 仪器

名称	型号	生产商
超声波提取仪	KQ3200D	Kunshan Ultrasonic Instruments Co., Ltd.
高速冷冻离心机	Mikro 220R	Hettich Lab Technology
反相色谱柱	ACQUITY UPLC HSS T3 (2.1 mm × 100 mm, 1.8 μm)	Waters Corporation
超高效液相色谱	Vanquish Flex UHPLC	Thermo Fisher Scientific
四极杆轨道离子阱质谱	Q Exactive	Thermo Fisher Scientific

3.1 Instruments

Table 1 Instruments

Name	Model	Manufacturer
Ultrasonic extractor	KQ3200D	Kunshan Ultrasonic Instruments Co., Ltd.
High-speed refrigerated centrifuge	Mikro 220R	Hettich Lab Technology
Reversed-phase column	ACQUITY UPLC HSS T3 (1.8 μm, 2.1 mm × 100 mm)	Waters Corporation
Ultra-high performance liquid chromatograph	Vanquish Flex UHPLC	Thermo Fisher Scientific
Hybrid quadrupole orbitrap mass spectrometer	Q Exactive	Thermo Fisher Scientific

3.2 试剂

表 2 试剂

名称	规格	货号	生产商
水	LC-MS/4 L	W6-4	Thermo Fisher Scientific
甲醇	LC-MS/4 L	A456-4	Thermo Fisher Scientific
乙腈	LC-MS/4 L	A955-4	Thermo Fisher Scientific
甲酸	LC-MS/50 mL	A117-50	Thermo Fisher Scientific

3.2 Reagents

Table 2 Reagents

Name	Grade	Article No.	Manufacturer
Water	LC-MS/4 L	W6-4	Thermo Fisher Scientific
Methanol	LC-MS/4 L	A456-4	Thermo Fisher Scientific
Acetonitrile	LC-MS/4 L	A955-4	Thermo Fisher Scientific
Formic acid	LC-MS/50 mL	A117-50	Thermo Fisher Scientific

3.3 实验方法

3.3 Experiment

3.3.1 样品制备

取中药样品 1.0 g，置于 50 mL 离心管中，加入 20 mL 80%甲醇，超声 1 h。取 1 mL 溶液，置于 1.5 mL 离心管中，于 4℃、12000 rpm 条件下离心 10 min。取上清液 100 μL，加入 100 μL 超纯水，混匀后，置于进样瓶中，待测。

取 100 μL 血清样品，置于 1.5 mL 离心管中，加入 300 μL 甲醇，涡旋振摇 10 min。将离心管置于低温离心机中，4℃、12000 rpm 下离心 10 min。取上清液 270 μL，真空离心浓缩 4 h。加入 90 μL 50%甲醇水溶液，涡旋振摇 1 min。将离心管置于低温离心机中，4℃、12000 rpm 下离心 10 min。取上清液 80 μL，置于进样瓶中，待测。

3.3.1 Sample preparation

1.0 g of traditional Chinese medicine sample and 20 mL of 80% methanol were charged into a 50 mL centrifuge tube, and ultrasonicated for 1 h. After that, 1 mL suspension was transferred into a 1.5 mL tube and centrifuged at 4°C and 12000 rpm for 10 min. Finally, 100 μL of supernatant was mixed with 100 μL ultrapure water and then pipetted into an injection vial for detection.

100 μL of serum and 300 μL of methanol were transferred into a 1.5 mL centrifuge tube and vortexed for 10 min. The tube was then centrifuged at 4°C and 12000 rpm for 10 min. 270 μL of supernatant was charged into a 1.5 mL centrifuge tube and concentrated by vacuum centrifuge for 4 h. Subsequently, 90 μL 50% methanol water solution was added into the tube and vortexed for 1 min. The tube was then centrifuged at 4°C and 12000 rpm for 10 min. 80 μL of supernatant was transferred into an injection vial for analysis.

3.3.2 色谱方法

表 3 洗脱梯度表

时间（min）	流动相	
	A（v%）	B（v%）
0	98	2
1.0	98	2
14.0	70	30
25.0	0	100

28.0	0	100
28.1	98	2
30.0	98	2

采用配备 ACQUITY UPLC HSS T3 色谱柱（2.1 mm（内径）× 100 mm（长度），1.8 μm（粒径））（Waters Corp., Milford, MA, USA）的 Vanquish Flex UHPLC（Thermo Fisher Scientific Inc., Waltham, MA, USA）超高效液相色谱进行色谱分离。流动相由 A 相（水+0.1%甲酸）和 B 相（乙腈）组成，洗脱梯度参见表 3，流速为 0.3 mL/min。色谱柱温度为 40°C。进样量为 6.0 μL。

3.3.2 Chromatographic conditions

Table 3 Elution gradient

Time (min)	Mobile phase	
	A (v%)	B (v%)
0	98	2
1.0	98	2
14.0	70	30
25.0	0	100
28.0	0	100
28.1	98	2
30.0	98	2

A Vanquish Flex UHPLC chromatograph (Thermo Fisher Scientific, Waltham, MA, USA) equipped with an ACQUITY UPLC HSS T3 column (2.1 mm (inner diameter) ×100 mm (length), 1.7 μm (particle dimension)) (Waters Corp., Milford, MA, USA) was used for separation. The mobile phase was consisted of water (0.1% formic acid, phase A) and acetonitrile (phase B) with a flow rate of 0.3 mL/min and the column temperature was 40°C. The elution gradient was shown in Table 3 and the injection volume was 6.0 μL.

3.3.3 质谱方法

采用配备热电喷雾离子源的四极杆轨道离子阱质谱仪（Q Exactive, Thermo Fisher Scientific, Waltham, MA, USA）进行质谱分析。正、负离子的离子源电压分别为 3.7 kV 和 3.5 kV；毛细管加热温度为 320°C；鞘气压力为 30 psi，辅助气压力为 10 psi；溶剂加热蒸发温度为 300°C；鞘气和辅助气均为氮气；碰撞气为氮气，压力为 1.5 mTorr。采用 Full scan/dd-MS^2

模式采集数据，Full scan 参数：分辨率 70000，自动增益控制目标 1×10^{6}，最大隔离时间 50 ms，质荷比扫描范围 100 – 1500；dd-MS^{2} 参数：分辨率 17500，自动增益控制目标 1×10^{5}，最大隔离时间 50 ms，最多扫描 10 个离子的二级碎片（动态排除），质量分离窗口 2，碰撞能 10 V、30 V、60 V，强度限定 1×10^{5}。

3.3.3 MS conditions

The MS data was collected by a hybrid quadrupole orbitrap mass spectrometer (Q Exactive, Thermo Fisher Scientific, Waltham, MA, USA) equipped with a HESI-II spray probe. The parameters were set as follows: positive ion source voltage 3.7 kV and negative ion source voltage 3.5 kV, heated capillary temperature 320°C, sheath gas pressure 30 psi, auxiliary gas pressure 10 psi, desolvation temperature 300°C. Both the sheath gas and the auxiliary gas were nitrogen. The collision gas was also nitrogen with a pressure of 1.5 mTorr. The data was acquired in “Full scan/dd-MS^{2}” mode. The parameters of the full scan were set as follows: resolution 70000, auto gain control target 1×10^{6}, maximum isolation time 50 ms and m/z scan range 100 – 1500. The dd-MS^{2} data was collected with the parameters of resolution 17500, auto gain control target 1×10^{5}, maximum isolation time 50 ms, top n (n≤10) most intense parent ions selected for fragmentation coupled with dynamic exclusion mechanism, isolation window of m/z 2, collision energy 10 V, 30 V, 60 V and intensity threshold 1×10^{5}.

3.4 数据分析

采用 Progenesis QI 3.0 软件（Waters Corp., Milford, MA, USA）处理采集的质谱数据，步骤依次为导入原始数据、峰提取、去卷积。搜索对照品数据库（TCM Pro 2.0）和理论数据库（通过文献、公共数据库等构建），通过对照品保留时间误差、母离子质量误差、二级碎片匹配、同位素分布、峰强度等对鉴定结果进行多维综合判断分析，得到最终结果。

3.4 Data analysis

The MS data was processed by Progenesis QI 3.0 (Waters Corp., Milford, MA, USA) with the steps of raw data introduction, peak extraction and deconvolution. The identification was finally determined by in consideration of retention time error of reference substance, mass error of parent ion, match degree of daughter ions, isotope distribution and peak area after searching the reference substance database (TCM Pro 2.0) and theoretical database constructed by literature and public databases.

4 实验结果

4 Results

4.1 谱图

4.1 Chromatogram

高清图详见“附图”文件夹。

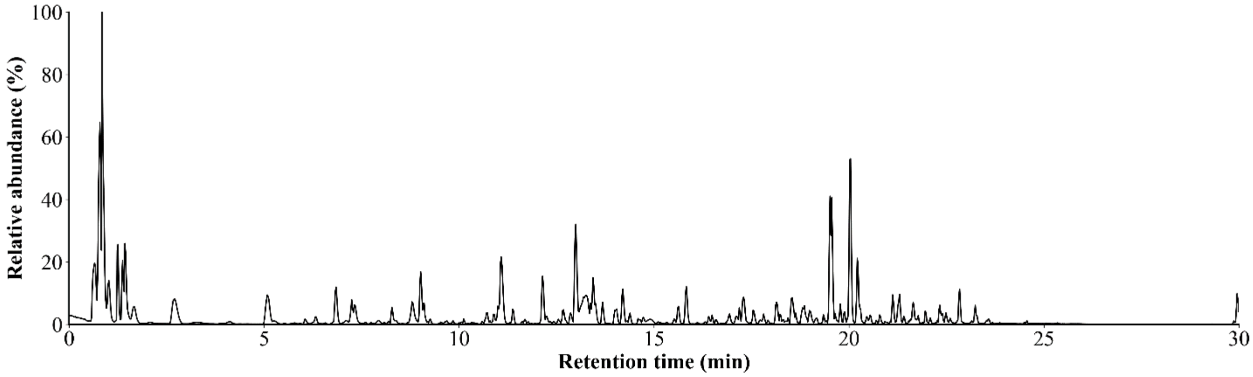

图 2 中药样品正离子模式检测基峰图

Fig.2 Base peak ions (BPI) chromatogram of traditional Chinese medicine detected in positive mode.

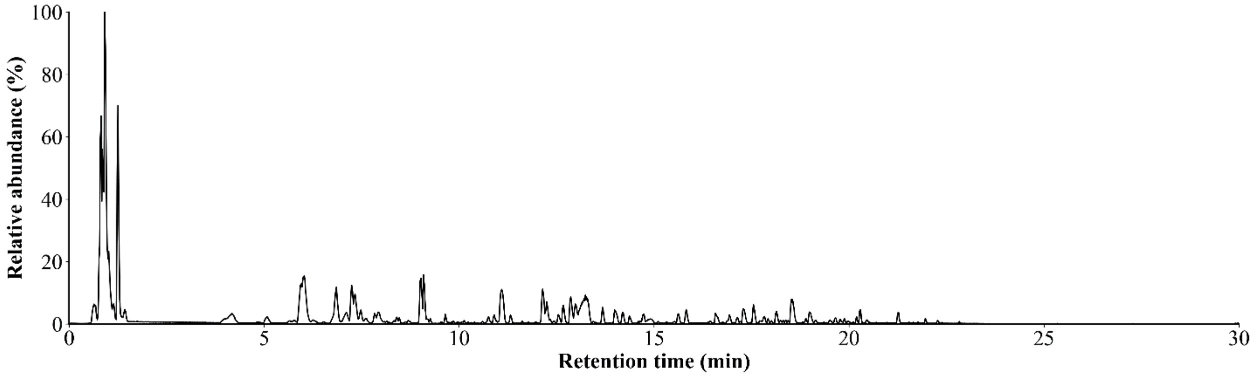

图 3 中药样品负离子模式检测基峰图

Fig.3 BPI chromatogram of traditional Chinese medicine detected in negative mode.

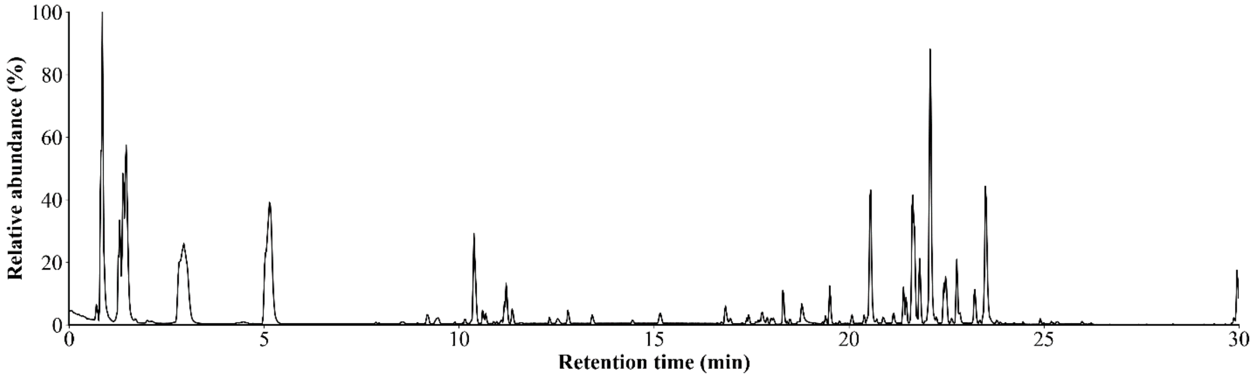

图 4 含药血清样品正离子模式检测基峰图

Fig.4 BPI chromatogram of TCM-containing serum detected in positive mode.

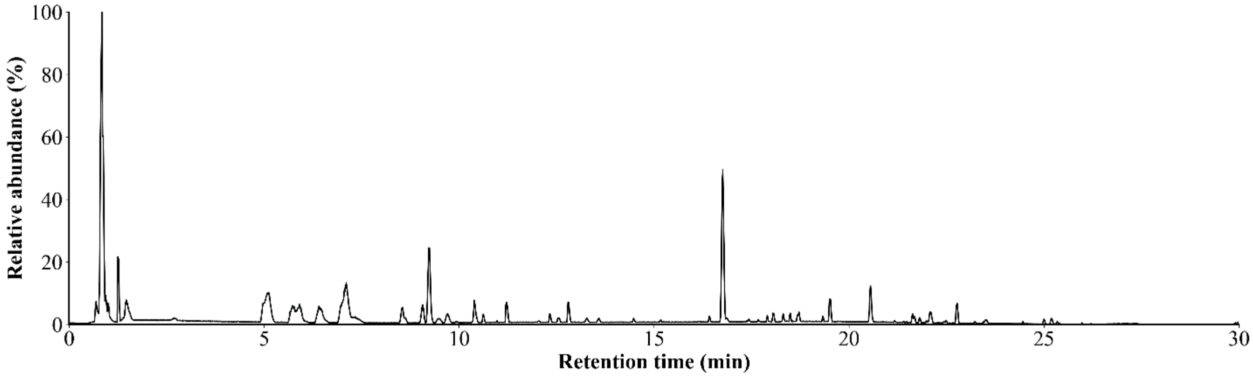

图 5 含药血清样品负离子模式检测基峰图

Fig.5 BPI chromatogram of TCM-containing serum detected in negative mode.

注：BPI 色谱图由每一时间点丰度最大的离子特征连续绘制而成；总离子流（Total ions chromatogram，TIC）色谱图则由每一时间点各离子特征丰度总和连续绘制而成。相比后者，前者具有基线低、丰度高、信号显著、更加美观等优点，故为许多中药研究相关文献所采用。如您需要 TIC 图，请联系销售人员获取。

4.2 鉴定结果

4.2 Identification results

鉴定结果详见“结果”文件夹>“中药鉴定结果”、“中药体内成分鉴定结果”。

5 参考资料

为便于您更方便、更清晰地理解、使用本项目的结果，我司提供了相关资料，详见“参考资料”文件夹。其中，**我司参考多篇文献报道及既往经验总结归纳得到常见的中药鉴定结果、中药体内成分鉴定结果的报道方式，详见 2_结果呈现示例文件**，供您参考。如有其他需求，可联系销售人员，我们将竭诚为您服务！

1_鉴定结果名词释义.pdf

2_结果呈现示例.pdf

6 参考文献

[1] https://www.gov.cn/zhengce/2019-10/26/content_5445377.htm

[2] 杨波, 韩莹, 张清宇, 董辉, 孙晖, 王喜军. 云南白药配伍环境中草乌活血化瘀作用的血中移行成分研究. 中国中药杂志, 2019, 44(15), 3349－3357.

[3] Yuan Chen, Han Li, Xinlian Zhang, Wei Wang, Marwan M.A. Rashed, Hong Duan, Lili Li, Kefeng Zhai. Exploring the anti-skin inflammation substances and mechanism of *Paeonia lactiflora* Pall. Flower *via* network pharmacology-HPLC integration. Phytomedicine, 2024, 129, 15565.

[4] Haiyu Xu, Yanqiong Zhang, Ping Wang, Junhong Zhang, Hong Chen, Luoqi Zhang, Xia Du, Chunhui Zhao, Dan Wu, Feng Liu, Hongjun Yang, Changxiao Liu. A comprehensive review of integrative pharmacology-based investigation: A paradigm shift in traditional Chinese medicine. Acta Pharmaceutica Sinica B, 2021, 11(6), 1379 − 1399.

[5] Ying Han, Hui Sun, Aihua Zhang, Guangli Yan, Xijun Wang. Chinmedomics, a new strategy for evaluating the therapeutic efficacy of herbal medicines. Pharmacology & Therapeutics, 2020, 216, 107680.

[6] Tianyang Wang, Song Lin, Hua Li, Ran Liu, Zihan Liu, Huarong Xu, Qing Li, Kaishun Bi, A stepwise integrated multi-system to screen quality markers of Chinese classic prescription Qingzao Jiufei decoction on the treatment of acute lung injury by combining 'network pharmacology-metabolomics-PK/PD modeling'. Phytomedicine, 2020, 78, 153313.

[7] Shi Dong, Peipei Wang, Liubo Zhang, Xiaotian Zhang, Xiaorui Li, Jiali Wang, Xinming Cui, Ting Lan, Can Gao, Yuanyuan Shi, Weijia Wang, Jianxun Wang, Miao Jiang. The Qi Yin San Liang San decoction enhances anti-CD19 CAR-T cell function in the treatment of B-cell lymphomas. Journal of Ethnopharmacology, 2024, 319, 117109.

[8] 刘昌孝, 陈士林, 肖小河, 张铁军, 侯文彬, 廖茂梁, 中药质量标志物（Q-Marker）：中药产品质量控制的新概念. 中草药, 2016, 47(9), 1443 − 1457.

[9] 张铁军, 白钢, 陈常青, 许浚, 韩彦琪, 龚苏晓, 张洪兵, 刘昌孝. 基于“五原则”的复方中药质量标志物（Q-marker）研究路径. 中草药, 2018, 49(1): 1 − 13.

[10] 张铁军, 白钢, 刘昌孝. 中药质量标志物的概念、核心理论与研究方法. 药学学报. 2019, 54(2), 187 − 196.

[11] Yaling Deng, Xianwen Ye, Yufan Chen, Hongmin Ren, Lanting Xia, Ying Liu, Minmin Liu, Haiping Liu, Huangang Zhang, Kairui Wang, Jinlian Zhang, Zhongwei Zhang. Chemical characteristics of *Platycodon grandiflorum* and its mechanism in lung cancer treatment. Frontiers in Pharmacology, 2021, 11, 609825.

[12] https://www.sciencedirect.com/journal/phytomedicine/publish/guide-for-authors

[13] https://www.sciencedirect.com/journal/journal-of-ethnopharmacology/publish/guide-

for-authors
[14] 邓哲, 荆文光, 刘安. 薄层色谱法在当前中药质量标准中的应用探讨. 中国实验方剂学杂志, 2019, 25(7), 201 − 206.
[15] 刘春丽, 刘满仓, 朱彭龄. 高效液相色谱法分析中药及植物药的进展. 2020, 28(5), 631-643.
[16] 国家药监局. 国家药监局关于促进中药传承创新发展的实施意见. 国药监药注[2020] 27 号.
[17] Yang Yu, Changliang Yao, De-an Guo. Insight into chemical basis of traditional Chinese medicine based on the state-of-the-art techniques of liquid chromatography-mass spectrometry. Acta Pharmaceutica Sinica B, 2021, 11(6), 1469 – 1492.

BPI chromatogram figures

#U4e2d#U836f#U6b63#U79bb#U5b50BPI#U9ad8#U6e05#U7ed8#U5236#U56fe.tif

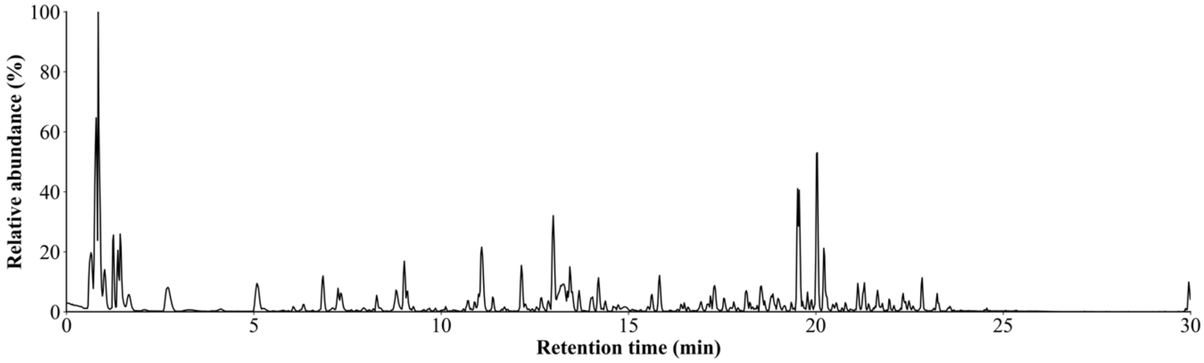

#U4e2d#U836f#U8d1f#U79bb#U5b50BPI#U9ad8#U6e05#U7ed8#U5236#U56fe.tif

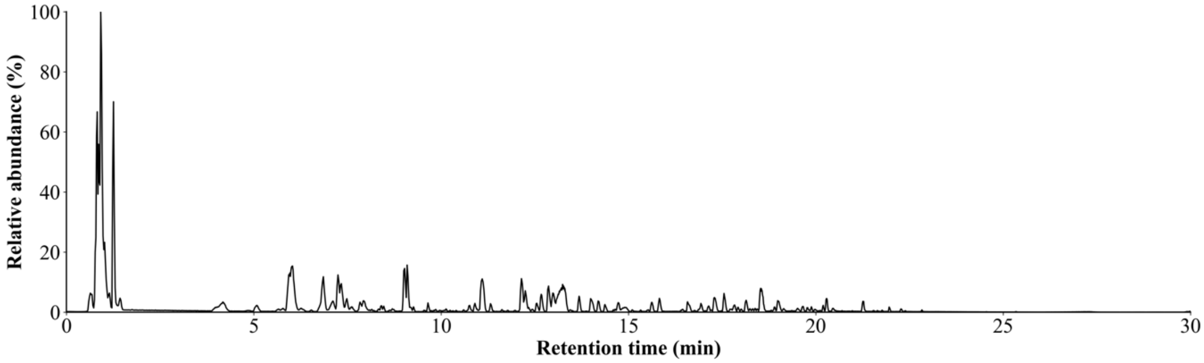

#U542b#U836f#U8840#U6b63#U79bb#U5b50BPI#U9ad8#U6e05#U7ed8#U5236#U56fe.tif

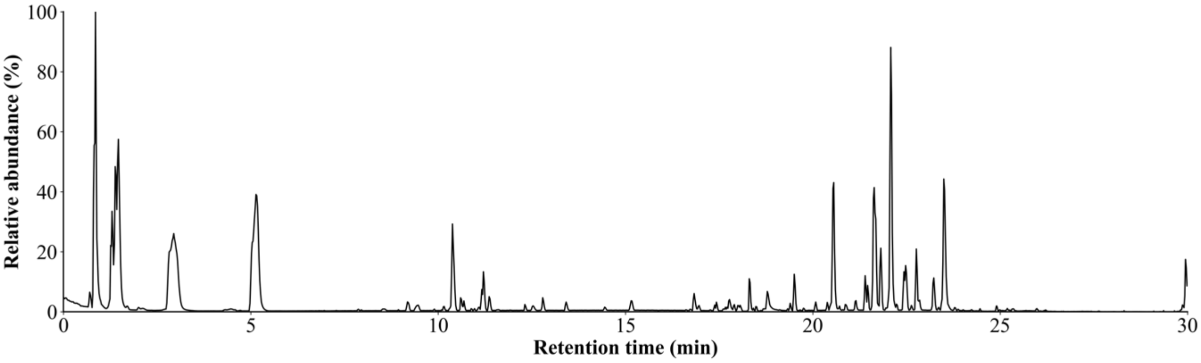

#U542b#U836f#U8840#U8d1f#U79bb#U5b50BPI#U9ad8#U6e05#U7ed8#U5236#U56fe.tif

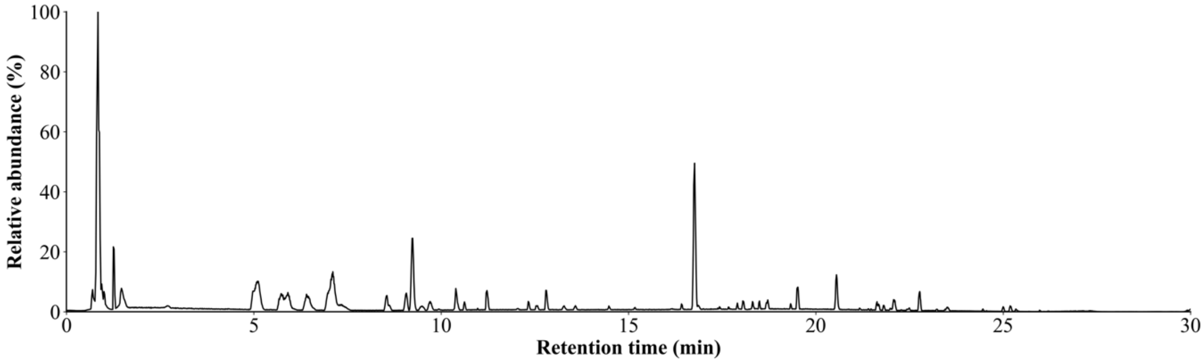

#U7a7a#U767d#U8840#U6b63#U79bb#U5b50BPI#U9ad8#U6e05#U7ed8#U5236#U56fe.tif

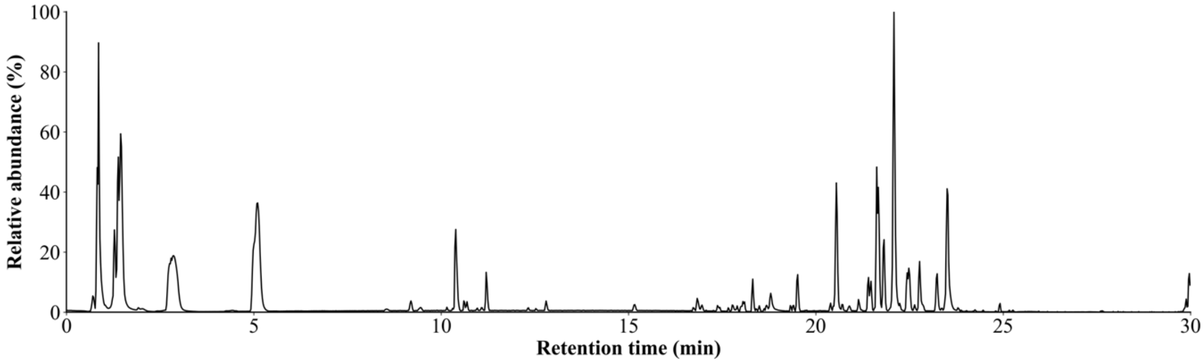

#U7a7a#U767d#U8840#U8d1f#U79bb#U5b50BPI#U9ad8#U6e05#U7ed8#U5236#U56fe.tif

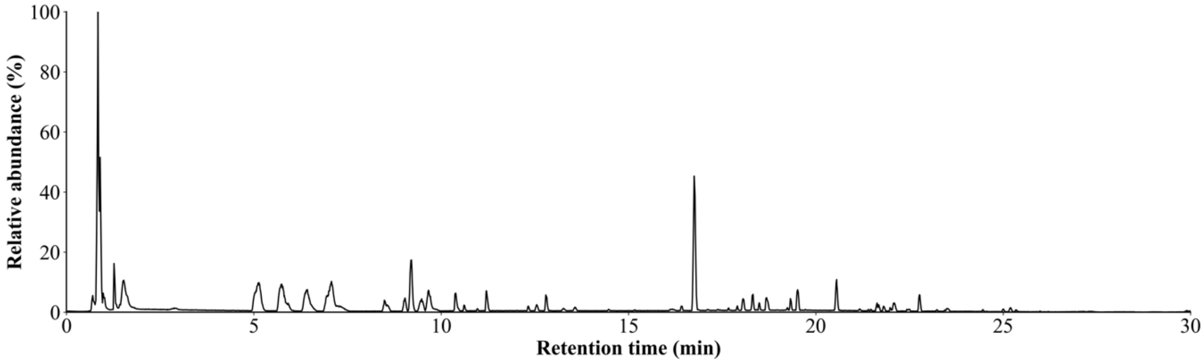

Supplement: Supplementary file 7 [file DataSheet5.pdf]
